# PhytoOracle: Scalable, modular phenomics data processing pipelines

**DOI:** 10.3389/fpls.2023.1112973

**Published:** 2023-03-06

**Authors:** Emmanuel M. Gonzalez, Ariyan Zarei, Nathanial Hendler, Travis Simmons, Arman Zarei, Jeffrey Demieville, Robert Strand, Bruno Rozzi, Sebastian Calleja, Holly Ellingson, Michele Cosi, Sean Davey, Dean O. Lavelle, Maria José Truco, Tyson L. Swetnam, Nirav Merchant, Richard W. Michelmore, Eric Lyons, Duke Pauli

**Affiliations:** ^1^ School of Plant Sciences, University of Arizona, Tucson, AZ, United States; ^2^ Department of Computer Science, University of Arizona, Tucson, AZ, United States; ^3^ Department of Computer Engineering, Sharif University of Technology, Tehran, Iran; ^4^ Data Science Institute, University of Arizona, Tucson, AZ, United States; ^5^ BIO5 Institute, University of Arizona, Tucson, AZ, United States; ^6^ Department of Cellular and Molecular Medicine, University of Arizona, Tucson, AZ, United States; ^7^ The Genome and Biomedical Sciences Facility, University of California, Davis, Davis, CA, United States; ^8^ School of Natural Resources and the Environment, University of Arizona, Tucson, AZ, United States; ^9^ Department of Plant Sciences, University of California, Davis, Davis, CA, United States

**Keywords:** phenomics, morphological phenotyping, physiological phenotyping, distributed computing, high performance computing, image analysis, point cloud analysis, data management

## Abstract

As phenomics data volume and dimensionality increase due to advancements in sensor technology, there is an urgent need to develop and implement scalable data processing pipelines. Current phenomics data processing pipelines lack modularity, extensibility, and processing distribution across sensor modalities and phenotyping platforms. To address these challenges, we developed PhytoOracle (PO), a suite of modular, scalable pipelines for processing large volumes of field phenomics RGB, thermal, PSII chlorophyll fluorescence 2D images, and 3D point clouds. PhytoOracle aims to (*i*) improve data processing efficiency; (*ii*) provide an extensible, reproducible computing framework; and (*iii*) enable data fusion of multi-modal phenomics data. PhytoOracle integrates open-source distributed computing frameworks for parallel processing on high-performance computing, cloud, and local computing environments. Each pipeline component is available as a standalone container, providing transferability, extensibility, and reproducibility. The PO pipeline extracts and associates individual plant traits across sensor modalities and collection time points, representing a unique multi-system approach to addressing the genotype-phenotype gap. To date, PO supports lettuce and sorghum phenotypic trait extraction, with a goal of widening the range of supported species in the future. At the maximum number of cores tested in this study (1,024 cores), PO processing times were: 235 minutes for 9,270 RGB images (140.7 GB), 235 minutes for 9,270 thermal images (5.4 GB), and 13 minutes for 39,678 PSII images (86.2 GB). These processing times represent end-to-end processing, from raw data to fully processed numerical phenotypic trait data. Repeatability values of 0.39-0.95 (bounding area), 0.81-0.95 (axis-aligned bounding volume), 0.79-0.94 (oriented bounding volume), 0.83-0.95 (plant height), and 0.81-0.95 (number of points) were observed in Field Scanalyzer data. We also show the ability of PO to process drone data with a repeatability of 0.55-0.95 (bounding area).

## 1 Introduction

The world population is expected to reach 10 billion people by 2050 with a projected 50% decrease in global freshwater resources (Searchinger et al., 2019; Gupta et al., 2020). Although existing crop improvement methods have maintained stable increases in crop yields, a continuation of these trends is not sustainable (Grassini et al., 2013). Crop improvement methods continue to rely on subjective, manually collected phenotype data. However, advances in sensor technology have contributed to the emergence of plant phenomics, the study of plant phenotypes, over the last decade (Andrade-Sanchez et al., 2014; Araus and Cairns, 2014; Pauli et al., 2016). Low-cost, user-friendly sensors now enable the collection of objective data at high throughput. The resulting data volumes are substantial and reveal bottlenecks in data processing, data management, and data storage. To date, a variety of phenomics bottlenecks related to data collection have been resolved, but computational bottlenecks related to data volume and velocity have been largely overlooked (Furbank and Tester, 2011). The volume and velocity of plant phenomics data collection makes it difficult to extract phenotypic trait data using existing software at the scale required for breeding programs and basic research. Therefore, addressing bottlenecks in computational throughput would enable the efficient processing of data and, as a result, the study of variation and plasticity of fine-scale traits at high temporal resolution. These high-resolution datasets may improve the elucidation of genetic components controlling agronomic and functional traits (Furbank and Tester, 2011).

Phenotyping, various marker technologies, and statistical methods have enabled the prediction of genotypic values and genetic mapping (Bernardo, 2020). The application of these methods allows for the dissection of the genetic and environmental components of phenotypic trait variance. Such studies require the measurement of quantitative traits that are often collected visually, in the case of observational data, and manually using handheld devices such as PAM fluorometers for chlorophyll fluorescence measurements, spectroradiometers for UV-VIS-NIR, protractors for leaf angle, rulers for plant height, and weight scales for yield. Visual and manual phenotyping are common due to having low initial investment costs, but these approaches lack throughput and reproducibility due to the labor required and subjectivity of measurements (Reynolds et al., 2019). Emerging technologies, such as automated high-throughput plant phenotyping platforms, often have higher initial investment costs compared to traditional phenotype collection, but this is quickly changing. High-throughput platforms are diverse, including robots, drones, phones, and carts (White and Conley, 2013; Bai et al., 2016; Thompson et al., 2018; Thorp et al., 2018; Yuan et al., 2018; Guo et al., 2020; Roth et al., 2020). Compared with traditional methods, these platforms improve data collection throughput, reduce subjectivity through varying levels of automation, and enable higher phenotyping resolution, referred to here as fine-scale phenotyping (Reynolds et al., 2019). The resolution provided by fine-scale phenotyping has enabled studies revealing genetic loci associated with drought resistance (Li et al., 2020), stomatal conductance (Prado et al., 2018), temporal salinity responses (Campbell et al., 2015), and panicle architecture (Rebolledo et al., 2016). Other studies have captured natural variation in photosynthetic efficiency (van Bezouw et al., 2019; Khan et al., 2020) as well as highlighted the feasibility of phenomics selection (Rincent et al., 2018; Parmley et al., 2019; Zhu et al., 2021) based on traits such as stay-green (Rebetzke et al., 2016) and spectral reflectance (Aguate et al., 2017; Lane et al., 2020).

The high temporal and spatial resolution of fine-scale phenotyping using automated plant phenotyping platforms provide new opportunities to study dynamic patterns in phenotype expression in response to varying conditions. For example, the phenotypic effects of induced variation can be assessed in mutant populations and natural variation in diversity panels (Khan et al., 2020), allowing for the detection of temporal fluctuations in trait expression and associations between morphological and physiological phenotypic traits. Future research and development in computational plant phenomics could help improve selection accuracy due, in part, to increasingly precise extraction of fine-scale phenotypes enabled by complementary analytical methods and algorithms. In plant phenomics, the level of extraction required to dissect agronomic and functional traits would involve processing large volumes of image, spectral, and point cloud raw data across thousands of plants and time points to identify unique, obscure patterns of morphophysiological responses to various environments. The integration of these fine scale phenomics datasets within and across projects would further expand our knowledge of traits and aid in hypothesis generation (Coppens et al., 2017).

The data volumes generated by biological sciences research outpace existing computing infrastructure (Chen et al., 2013; Qin et al., 2015; Stephens et al., 2015; Sivarajah et al., 2017). Additionally, data variety within biological sciences research is widening due to the emergence of phenomics, particularly in plant science research (Furbank and Tester, 2011; Furbank et al., 2019; Harfouche et al., 2019). The increasing availability and diversity of modular, high-quality sensors mounted on automated phenotyping platforms has led to the collection of large volumes of various data types, including morphological and physiological traits (Coppens et al., 2017). These expanding data volumes pose new challenges related to computation, data integration, and data management – a problem that is likely to be exacerbated by continued improvements and widespread use of sensor technology (Kim et al., 2017). In information science, it has long been recognized that existing computational techniques are inadequate in dealing with big data, primarily due to bottlenecks in the extraction of information from large volumes of data and the associated bottlenecks of scalability and data management. The bottleneck in information extraction is actively being addressed through the development of methods including machine learning (ML) and artificial intelligence (AI), while parallel processing is addressing scalability (Chen et al., 2013; Jukić et al., 2015; Sivarajah et al., 2017). Although these methods improve scalability and information extraction, they do not address data management. Parallel computing systems (PCSs) are characterized by the co-location of input data and processing code, representation of processing in terms of data flows and transformations, and scalability. Collectively, these characteristics facilitate the processing of datasets once considered intractable due to previous limitations in computing (Kale, 2020). The required computational resources in PCSs are commonly data-dependent, meaning that each dataset requires a different set of computational resources. To increase processing efficiency and reduce computing costs, PCSs could allow users to tailor CPU/GPU, high-memory/high-processor nodes, and other computational resources to specific datasets. This capability may become increasingly important as expanding data volumes pose a higher cost if computational resources are used inefficiently.

For phenomics data to provide actionable genome-phenome insights in combination with other -omics data, large scale phenomics data must be processed in a scalable and reproducible manner, stored in publicly accessible data stores, and be interoperable with other data types (Coppens et al., 2017; Kim et al., 2017). To address these requisites, a variety of established resources can be leveraged. For example, data management systems such as the CyVerse Data Store, a cloud-based data management system built on the Integrated Rule-Oriented Data System (iRODS), provides storage and cross-platform command line interface (CLI) access to data (Goff et al., 2011; Merchant et al., 2016). Container technologies, such as Docker and Singularity, serve as stand-alone environments with required dependencies pre-installed by software developers for increased extensibility (Kurtzer et al., 2017). High performance computers (HPCs) supply numerous processors, dual in-line memory modules (DIMMs), internal disk, and networking ports to scale up processing tasks. Container technology and data management systems coupled with HPCs provide reproducible and scalable environments, respectively (Devisetty et al., 2016; Kurtzer et al., 2017). Large volume datasets further require advanced PCSs capable of leveraging thousands of computers or cluster nodes for parallel processing on local, cloud, and/or HPC compute resources. A suite of computing tools for deploying scalable applications known as the Cooperative Computing Tools (CCTools) consists of Makeflow and Work Queue, a language and computational resource management framework for distributed computing, respectively (Albrecht et al., 2012). When coordinated, the above-mentioned computational resources can improve the processing and management of raw data and enable large scale analyses of extracted phenomics data.

Several image analysis pipelines exist for morphological and physiological phenotype trait extraction including: ImageHarvest (Knecht et al., 2016); Greenotyper (Tausen et al., 2020); and PlantCV (Fahlgren et al., 2015; Gehan et al., 2017). Most of these software were developed for automated phenotyping platforms in controlled greenhouse environments and would require significant modification for processing field phenomics data due to variations in image illumination and the lack of spacing between plants in field settings. Although some pipelines integrate multi-processing or distributed computing capabilities, there is currently no published pipeline that integrates data management systems, container technologies, PCSs, and multi-system deployment within a single framework. Importantly, many existing image analysis software were not designed to enable customization of computational resources, a critical component for efficiently processing phenomics’ expanding data volumes (Kale, 2020).

Here, we present PhytoOracle (PO), a suite of data processing pipelines for phenomics data processing. PhytoOracle combines data management systems, container technologies, distributed computing, and multi-system deployment into a single framework capable of processing phenomics data collected with RGB cameras (RGB), photosystem II chlorophyll fluorescence imagers (PSII), thermal cameras (thermal), structured-light laser scanners (3D). Each pipeline component is containerized and can be removed, replaced, rearranged, or deployed in isolation. PhytoOracle provides advanced PCS and automation capabilities for processing large phenomics datasets across HPC, cloud, and/or local computing environments. The PO suite organizes all processing tasks and computational resource specifications within a single YAML file, which enables customization of computational resources, processing modules, and data management systems. Users can target pipelines to the optimal computational resources whether that be high-memory, high-processor, and/or GPU nodes. The modularity and distributed computing capabilities of PO enable the efficient extraction of time series, individual plant phenotypic trait data from large, multi-modal phenomics datasets. The PCSs like PO improve data analysis and information processing, providing large scale data that can help answer questions that were previously intractable due to data volumes outpacing computing systems’ capacities.

## 2 Materials and methods

### 2.1 Plant material

For this study, a panel of 241 lettuce genotypes were evaluated at the University of Arizona’s Maricopa Agricultural Center (MAC) in Maricopa, Arizona (33°04’24.8” N 111°58’25.7” W). The soil type is a Casa Grande sandy loam (fine-loamy, mixed, superactive, hyperthermic Typic Natrargids). The panel consisted of two subpopulations of lettuce, a diversity panel (147 genotypes) that represented all major market classes of lettuce and a recombinant inbred line (RIL) mapping population (94 genotypes) developed from a cross of the cultivars “Iceberg” and “Grand Rapids.” The population was organized in a randomized incomplete block design with three replications of both lettuce panels per irrigation treatment level with common checks used throughout the field. The borders around each irrigation treatment were of the cultivar “Green Towers.” The three irrigation treatments were: well-watered (WW), level 1 drought (D1), and level 2 drought (D2) (**Supplementary Figure 1**). The WW treatment was defined as 24% volumetric soil water content (VSWC) which represents field capacity. To achieve the D1 and D2 conditions, 75% and 50% of the WW irrigation amounts were applied to the plots, respectively. Raised vegetable beds on 1.02 m row spacing were shaped to have a surface width of 0.56 m with two seed lines per bed spaced at 0.31 m; plots were 4.00 m in length. Experimental plots consisted of one of the individual seed lines per raised bed so that two genotypes were planted per raised bed.

The crop was established using sprinkler irrigation for the first 35 days before switching to subsurface drip irrigation. Buried within each bed, at a depth of 0.20 m, was pressure compensated drip tape (Model 06D63613.16-12, Netafim, Tel Aviv, Israel) supplying a constant 0.38 liters per hour of water. Soil moisture conditions were recorded using a neutron probe (Model 503, Campbell Pacific Nuclear, CPN, Martinez, CA, USA) with readings taken at depths of 10, 30, 50, 70, and 90 cm on a weekly basis. Neutron probe access tubes were distributed throughout the field to capture the VSWC across the different irrigation treatments over the growing period. Once plants were established and being irrigated with subsurface irrigation, plots were thinned to a density of 10 equidistant plants to facilitate individual plant phenotyping. After thinning, approximately 26,000 plants were present in the field, with each treatment containing approximately 9,000 plants. Standard cultivation practices and agronomic management for lettuce production in the Southwest were followed. A total of 1,472 plants, one from each plot within the WW and D2 treatments, were harvested and their fresh weights were recorded at the end of the growing period (2020-03-03).

### 2.2 Phenotyping platforms

**Figure 1:**
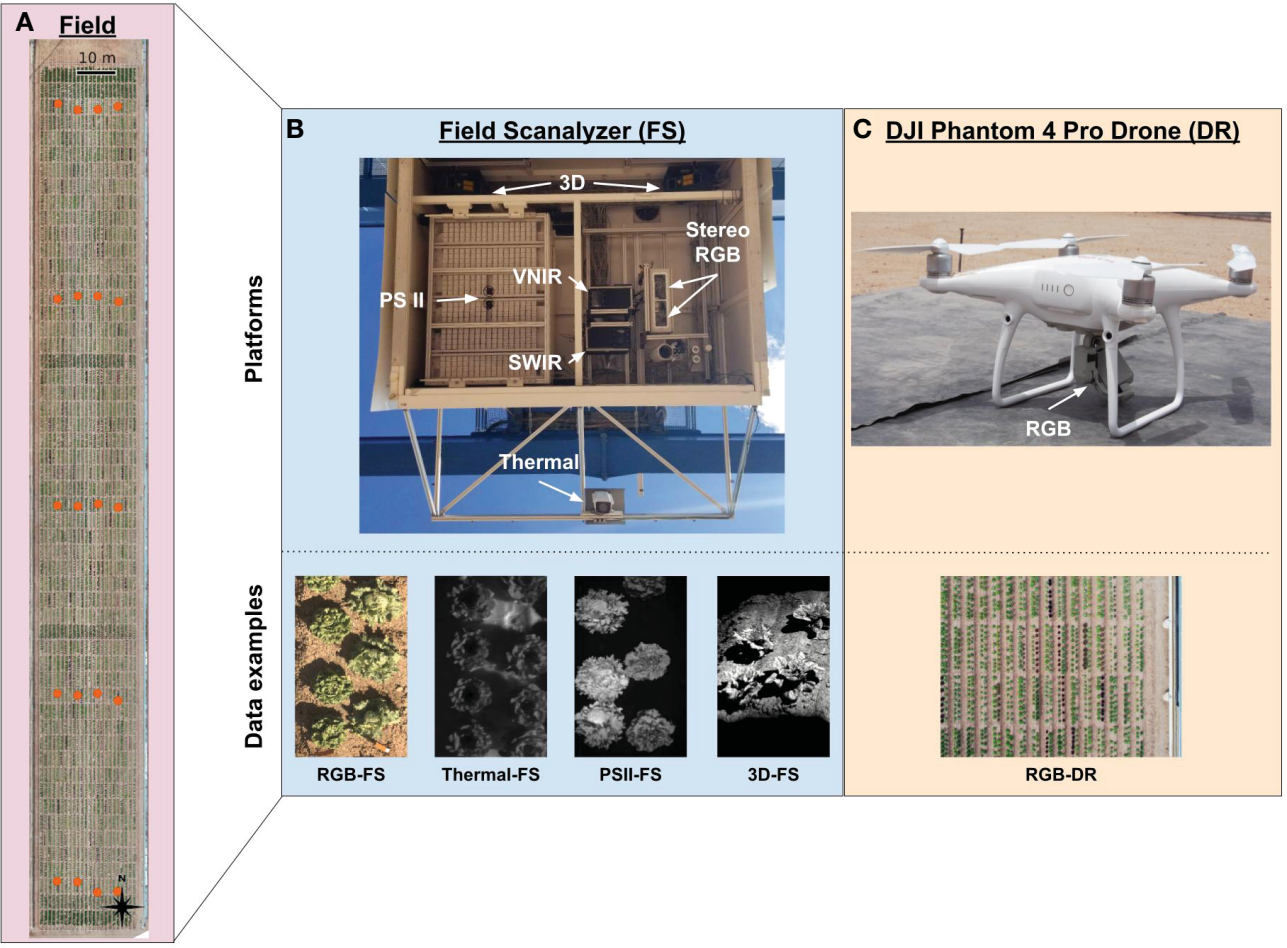
Overview of the Field Scanalyzer (FS) and DJI Phantom 4 Pro V2 drone (DR) phenotyping platforms, the components that make up each platform’s sensor array and resulting data types. **(A)** An aerial photograph showing the area scanned by the FS which totals 0.63 hectare. Orange dots indicate the ground control point (GCPs) configuration, consisting of five sets of four GCPs running east to west for a total of 20 GCPs. (Top **B**) The FS sensor box contains a photosystem II (PSII) chlorophyll fluorescence imager, stereo RGB cameras, a thermal camera, two pushbroom hyperspectral imagers (visible near-infrared [VNIR] and shortwave near-infrared [SWIR]), a pair of structured-light laser scanners, and environmental sensors. (Botton **B**) Collected data included RGB, thermal, and PSII 2D image data and 3D point cloud data. (Top **C**) The DJI Phantom 4 Pro V2 drone (DR) was equipped with a 20-megapixel RGB camera and flown with automated flight mapping software at an altitude of 15 meters. (Bottom **C**) Collected data included RGB 2D image data.

The Field Scanalyzer (FS) is a ground-based, automated phenotyping platform that moves along rails that are 394.1 m in length running North-South with 28 m separation between the rails; the area covered by the FS is approximately 1.11 hectares. This area is split into two fields with scannable areas of 0.37 hectare for the north field and 0.46 hectare for the south field; for the purposes of the present research, only the south field was used (**Figure 1A**). The FS is equipped with a ventilated sensor box that holds multiple imagers and cameras including the following: Allied Vision Prosilica GT3300C stereo RGB cameras (RGB), LemnaTec photosystem II chlorophyll fluorescence prototype imager (PSII), FLIR A615 thermal camera (Thermal), pair of Fraunhofer structured-light laser scanners (3D), and two Headwall HyperSpec Inspector pushbroom hyperspectral imagers (visible to near infrared [VNIR] and short-wave infrared [SWIR]) (**Figure 1B** and **Supplementary Table 1**). The sensor box can move vertically from 0.43 to 6.26 m above ground level to accommodate varying scanning distance requirements for each sensor and to maintain a consistent distance from the instrument to plant canopy throughout the growing season.

The FS scanning scheme is controlled by custom operating scripts that specify the scan area, pattern, and scheduling for data collection of each sensor. These operating scripts are set to collect data on specific regions of the field, agricultural plots, or the entire field by the FS operator. The RGB, thermal, and PSII sensors collect binary (BIN) format images, while the 3D laser scanners collect depth and reflectance imagery from which point clouds are generated using manufacturer-provided software (**Table 1**). Each data collection is accompanied by metadata files in JavaScript Object Notation (JSON) format containing FS variable position, sensor fixed position (location of sensors within sensor box), preset scanning area, and timestamps. Positioning information is collected by a series of barcodes along the rails (X and Y axes) and a string encoder (Z axis) using a right-handed coordinate system (+X South-to-North, +Y East-to-West, and +Z 0.76 cm above soil upwards). Additionally, environmental sensors collect and log information on downwelling irradiance, photosynthetically active radiation, air temperature, relative humidity, brightness, ambient air carbon dioxide concentration, precipitation, and wind velocity and direction all at 5-second intervals in JSON format.

**Table 1:** Data collection summary for Field Scanalyzer (FS) and drone (DR) phenotyping platforms of data types supported by PhytoOracle.

Data	Collection time	Concurrent scan	Scanning area	Data type	Benchmark data size	Total scans	Total size
RGB-FS	5	Thermal-FS	Full field	BIN	140	36	2.91
Thermal-FS	5	RGB-FS	Full field	BIN	5	36	0.10
PSII-FS	5	–	Paired-plot center	BIN	80	18	1.00
3D-FS	9	–	Full field	PLY	350	32	8.37
RGB-DR	0.5	–	Full field	JPEG	3	19	0.059

The scanning area listed as full field encompassed the south portion of the field (0.63 hectare). Benchmark data size, gigabytes; total size, terabytes.

Collection duration (hours) represents the time from first data capture to final data capture.

#### 2.2.1 Data collection and management

For this study, the FS scanned the south field during the day and night throughout a growing season, collecting high-resolution, time-series images and point cloud data. The total number of RGB, thermal, PSII, and 3D data collections were 36, 36, 13, and 46, respectively. The RGB, thermal, and 3D laser scanner data collections covered the entire field while PSII data collections covered the center of each bed within a single treatment (**Table 1**). The FS total raw data sizes for each sensor were as follows: 0.12 terabytes (TBs) for thermal, 1.19 TBs for PSII, 3.20 TBs for RGB, and 8.77 TBs for 3D. Altogether, the FS data collections resulted in 13.36 TBs of raw data for the lettuce trial (**Supplementary Figure 2**).

In addition to FS data, drone (DR) flights were conducted over the same 0.46-hectare south field on a weekly basis using a DJI Phantom 4 Pro V2 (DJI, Nanshan, Shenzhen, China) and DroneDeploy software (v. 4.2.1; DroneDeploy, San Francisco, CA, USA) installed on an Apple iPad Mini 4 (Model #MK9P2LL/A; Apple, Cupertino, CA, USA) (**Figure 1**). The flight mission settings were as follows: 15 m altitude, 80% front - 80% side overlaps, 0.41 cm/pixel ground sample distance, resulting in approximately 450 images per flight. In total, the DR collections resulted in 0.08 TBs of raw image data for the lettuce trial (**Supplementary Figure 2**). For a complete list of FS and DR data collection dates, refer to **Supplementary Table 2**.

#### 2.2.2 Data management

The FS data collections were temporarily stored on a platform-mounted server and transferred to a cache server located at MAC. After a three-day retention period, each data collection was programmatically archived, producing a single “.tar.gz” archive file per data collection (one sensor’s scan), and programmatically transferred to the CyVerse Data Store servers located in Tucson, AZ using Internet2. Each DR data collection was uploaded to the CyVerse Data Store manually. The DR and FS archives were placed in a publicly available location in the CyVerse Data Store for general use and CLI access during data processing (Goff et al., 2011) (see Data Availability Statement).

### 2.3 Parallel computing system

The PO pipelines require ML models for object detection and point cloud segmentation during data processing. Data must be annotated, models trained, and performance assessed before data processing can be performed. As such, a description of model training is presented before describing PO processing pipelines in detail. Together with a season-specific GeoJSON containing plot boundaries, a YAML file specifying processing tasks, and computational resources, PO can distribute tasks across processing nodes of an HPC.

#### 2.3.1 Training and assessing performance of machine learning models

##### 2.3.1.1 2D object detection

**Figure 2:**
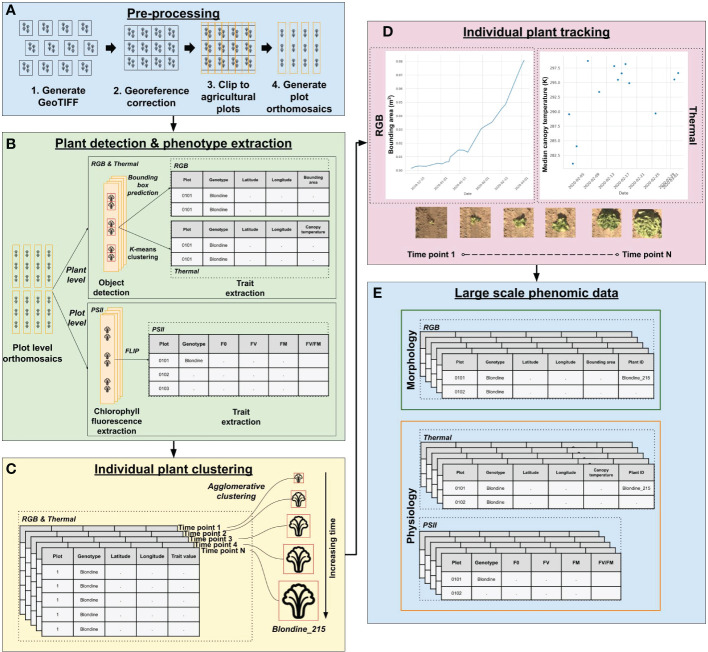
PhytoOracle two-dimensional (2D) image processing workflow. **(A)** The 2D pre-processing steps include the conversion of binary (BIN) files (RGB, thermal, PSII chlorophyll fluorescence) to GeoTIFF files, correction of georeferencing information within each GeoTIFF metadata using Megastitch for RGB and thermal data, clipping corrected GeoTIFF images to plots using a GeoJSON file with plot boundary information, and generation of plot level orthomosaics (Zarei et al., 2022). **(B)** RGB & thermal plot level orthomosaics are run through a Faster R-CNN detection model for plant detection and phenotype extraction; PSII images are run through FLIP for extraction of minimum (F_0_) and maximum (F_M_) fluorescence values, variable fluorescence (F_V_), and maximum yield of primary photochemical efficiency (F_V_/F_M_). **(C)** Upon completion of data processing for a single experiment, individual plant detections from RGB and thermal data are associated over time using agglomerative clustering. Agglomerative clustering uses longitude and latitude to associate multiple plant observations, giving them a shared, unique plant identifier. **(D)** The growth and temperature of individual plants can be tracked and visualized using the unique plant identifier. A merged, full season RGB and thermal data file can then be combined with PSII (plot level) and 3D laser phenotype data using the unique plant/plot identifiers. **(E)** The results of PhytoOracle are time series datasets with plant geographical coordinates of the bounding box predictions and plant centers; bounding area (BA); median and mean canopy temperatures (MEDT and MEAT, respectively); plant height (PH), axis-aligned and oriented bounding box volumes (AABV and OBV, respectively), and convex hull volume (CHV); and plot level F_0_, F_V_, F_M_, and F_V_/F_M_ for each detected plant.

To prepare image data for manual annotation, RGB and thermal data collections were processed up to the plot clip step to produce plot clipped orthomosaics (**Figure 2A**, Steps 1-4). Thermal and RGB plot clipped orthomosaics were converted from georeferenced Tag Image File Format (GeoTIFF) to PNG format (GeoTIFFs are not supported by annotation tools). Thermal image pixel values were normalized to the range of 0 to 255 to enhance visible features for manual annotation. Heat map images, with each pixel representing height, were generated from 3D point cloud data. The scripts for each of these steps is publicly accessible (see Code Availability Statement).

To train object detection ML models for RGB and thermal imagery, a total of 2,000 images per sensor type were randomly selected for developing training data (see Code Availability Statement). A total of 200 3D-derived heatmap images were randomly generated to train object detection ML models. The RGB, thermal, and 3D-derived heatmap image datasets were uploaded to Labelbox (http://labelbox.com; Labelbox, San Francisco, CA, USA) and manually labeled with a single bounding box around each plant. All images were manually reviewed to ensure label quality. A JSON file containing label bounding box coordinates for all images in a dataset was programmatically converted to XML files, resulting in one XML file per image (see Code Availability Statement). The RGB and thermal datasets were each randomly split into training, validation, and test sets (80%, 10%, and 10%, respectively). Transfer learning was employed to train a Faster R-CNN (region-based convolutional neural network) ResNet-50 FPN pre-trained model for RGB, thermal, and 3D-derived image datasets, separately, using the Detecto Python package (v. 1.2.1, http://github.com/alankbi/detecto) (Ren et al., 2017). The models for all data types were trained on a single label (“plant”). Training was performed on a HPC compute node with two AMD Zen2 48-core processors (AMD, Santa Clara, CA, USA), 512 GB of RAM, sixteen 32 GB memory DIMM, 2 TB SSD disk, and a V100S graphics processing unit (GPU) (NVIDIA, Santa Clara, CA, USA) with 32 GB memory. The selected setting of training parameters was 10 epochs, batch size of one, learning rate of 5 x 10^-3^, 5 x 10^-4^ weight decay (L2 regularization), and step size of three.

Model performance was assessed by calculating Intersection over Union (IoU), recall, precision, and F1 scores for RGB, thermal, and 3D-derived test datasets. To determine model performance more finely across the developmental stages of lettuce, we assessed IoU of randomly selected plots over the course of the season for RGB and thermal models. The IoU values were calculated as follows:


(1) $$IoU=\frac{\left|A\cap B\right|}{\left|A\cup B\right|}$$

where *A* is the area of the predicted bounding box, *B* is the area of the ground truth bounding box, and ∩ is the intersection and ∪ is the union of predicted and ground truth boxes. Detections with an IoU ≥ 0.5 were classified as true positives (TP, correctly detected plant), those with an IoU< 0.5 were classified as false positives (FP, plant is not present but detected), and detections with an IoU = 0 were classified as false negative (FN, plant is present but not detected). Recall, precision, and F1-score were calculated as follows:


(2) $$Recall\;=\;\frac{TP}{TP+FN}$$

(3) $$Precision\;=\;\frac{TP}{TP+FP}$$

(4) $$F1\;=\;2\cdot \;\frac{Precision\cdot Recall}{Precision+Recall}$$

##### 2.3.1.2 3D segmentation

To train segmentation ML models, a random sample of individual plant point clouds were collected and labeled using a model-assisted labeling (MAL) approach (Model-assisted labeling (MAL); Huxohl and Kummert, 2021). The MAL script fit a plane to each point cloud and resulted in the labeling of two classes: plant and soil (see Code Availability Statement). The results were visualized, and segmentation errors were manually corrected, resulting in a total of 160 annotated individual plant point clouds; plant point clouds were randomly split into train, validation, and test sets (80%, 10%, and 10%, respectively). A Dynamic Graph CNN (DGCNN) was trained on a server with four AMD EPYC 7702 64-Core processors (AMD, Santa Clara, CA, USA), 1 TB of RAM, and three NVIDIA Tesla T4 GPUs (NVIDIA, Santa Clara, CA, USA) (Wang et al., 2019). The following training parameters were selected: 30 epochs, learning rate of 0.01, 1 x 10^-4^, momentum of 0.9, and batch size of 32. The classes predicted by the DGCNN model for each point were compared with manually annotated data to collect TP, FP, TN, FN values, which were used to calculate the point-wise accuracy as follows:


(5) $$Point\;-\;wise\;accuracy\;=\;\frac{TP+TN}{TP+FP+TN+FN}$$

#### 2.3.2 Multimodal pipeline deployment

The processing instructions for PO data processing are defined in a Yet Another Markup Language (YAML) file (Ben-Kiki and Evans, 2001). The PO YAML template consists of four sections: “tags”, “modules”, “workload_manager”, and “paths”. The “tags” section allows users to define season-specific metadata for documentation purposes. The “modules” section is where users define their processing tasks by specifying the container to be used, the command to be run within the container, and the inputs and outputs. The user can select to run the workflow locally or remotely, that is using existing local cores or remote worker cores. The “workload_manager” key defines computational resource specifications required by pipeline worker nodes including the cores per worker, number of workers, and memory per core. The information provided within the “workload_manager” key is used to request jobs using the Slurm workload manager. Importantly, this allows users to customize the computing system to accommodate datasets of varying levels of processing scales and computational complexities. The “paths” section defines CyVerse Data Store paths for raw data download, including ML models to be used within the processing steps, and output data uploads. At the moment, only CyVerse Data Store paths are supported, but other storage providers can be supported with a few changes to the code. Users can specify their project-specific CyVerse Data Store paths or keep data locally without uploading it onto a data store. Users can select to use data transfer nodes, if running PO on HPC systems. Examples of YAML files for data processing of RGB, PSII, thermal, and 3D phenomics data of lettuce and sorghum are publicly available (see Code Availability Statement).

##### 2.3.2.1 RGB processing pipeline

The full field RGB-FS datasets each consisted of 9,270 BIN files. Each image capture collected two BIN files, one from each RGB camera, and an associated JSON metadata file. Due to the physical arrangement of the stereo RGB cameras and the resulting high image overlap, only one image of each capture was used in this study. The RGB pipeline consisted of four containerized components (**Supplementary Table 3** and **Supplementary Figure 3**). The first container converted BIN files to GeoTIFF images with approximate GPS bounding coordinates calculated from barcode positioning information contained within the JSON metadata file generated by the FS. The second container deployed MegaStitch, which is a software for efficient image stitching of large-scale image datasets (Zarei et al., 2022). Megastitch was run in a non-distributed manner as all images are required for the global optimization stitching method, which generated geometrically corrected GeoTIFFs. The third container clipped GeoTIFFs to plot boundaries using a GeoJSON file that delimits plots within the field. The fourth container deployed a Faster R-CNN model to detect individual plants within each plot-clipped orthomosaic, which output bounding box coordinates. Bounding box coordinates were converted from pixel coordinates to geographic coordinates using the geotransform information of each plot-clipped orthomosaic. All georeferencing was calculated in the World Geodetic System (WGS84) coordinate reference system (Lohmar, 1988). Longitude was calculated as follows:


(6) Longitude= a·x+b·y+a·0.5+b·0.5+c

where *c* is the upper left Easting coordinate of the image, *a* is the E-W pixel spacing, *c* is the rotation, and *x* and *y* are the bounding box image coordinates. Latitude was calculated as follows:


(7) Latitude = d·x+e·y+d·0.5+e·0.5+f

where *d* is the rotation, *e* is the N-S pixel spacing, *f* is the upper left Northing coordinate, and *x* and *y* are the bounding box image coordinates. The four geographical corner coordinates were converted to UTM coordinates and used to calculate plant bounding area (BA) as follows:


(8) $$\text{Plant bounding area }=\;(SE_{e}\;-\;NW_{e})\cdot (SE_{n}\;-\;NW_{n})$$

where *SE_e_* is the southeast corner Easting coordinate of the image, *NW_e_* is the northwest corner Easting coordinate, *SE_n_* is the southeast corner Northing coordinate, and *NW_n_* is the northwest corner Northing coordinate.

The RGB drone (RGB-DR) images from each data collection were processed using Pix4DMapper software (Pix4D S.A., Prilly, Switzerland). For each collection date, the “3D Maps” processing template was used, which generated an orthomosaic, point cloud, and depth maps. The “GCP/MTP Manager” interface was used to load GCP coordinates, co-align GCPs within images to known GCP coordinates, and confirm adequate placement of GCPs within the generated ray cloud. The resulting orthomosaics were processed using PO containers described above starting with the third container that clipped GeoTIFFs to plot boundaries.

##### 2.3.2.2 Thermal processing pipeline

The full field thermal-FS datasets each consisted of 9,270 BIN files. Each image capture collected one BIN file and an associated JSON metadata file. Each pixel within a thermal-FS image represents an uncalibrated digital number (DN), a dimensionless value corresponding to the output of the detector’s analog-digital conversion. The thermal pipeline consisted of four components (**Supplementary Table 3** and **Supplementary Figure 3**). The first container converted BIN files to GeoTIFFs with approximate GPS bounding coordinates calculated from barcode positioning information contained within the JSON metadata file. Thermal calibration measurements were applied to each pixel, converting the DN value to Celsius. The second container deployed MegaStitch (Zarei et al., 2022) in a non-distributed manner, which generated geometrically corrected GeoTIFFs. The third container clipped GeoTIFFs to plot boundaries specified within a GeoJSON file. The fourth container deployed a Faster R-CNN model to detect individual plants within each plot clipped GeoTIFF, which outputted bounding box coordinates. To collect individual plant canopy temperatures, each predicted bounding box, representing a single plant, was programmatically cropped from plot level GeoTIFF orthomosaics and *K*-means clustering was used with *K* = 3 (MacQueen, 1967; Poblete-Echeverría et al., 2017). The median and mean canopy temperatures (MEDT and MEAT, respectively) were collected from the plant pixel clusters for each plant along with corresponding distribution statistics. A 10x10 pixel region of interest (ROI) centered within each plant detection was analyzed for median temperature, referred to as the ROI temperature. The longitude and latitude for each plant detection were calculated using Equations 6, 7 respectively for subsequent plant tracking and multi-modal data association.

##### 2.3.2.3 PSII chlorophyll fluorescence processing pipeline

The PSII-FS datasets each consisted of 39,678 BIN files. Each data capture resulted in a 101-image stack over a 2-second interval along with an associated JSON metadata using a validated chlorophyll fluorescence imaging sensor (Herritt et al., 2020). Unlike RGB and thermal, these images captured the center of each plot instead of the full field. One image was captured shortly before LED light saturation, 50 images during the one-second saturating pulse of light, and 50 images after the pulse of light. The illuminating LED flash has a dominant wavelength in the range of 620-630 nm with an intensity of up to 7,000 μmol photosynthetically active radiation (PAR) at 70 cm from plant canopies. A modified version of the FLuorescence Imaging Pipeline (FLIP) software was used to extract plot level minimum fluorescence (*F_0_*), variable fluorescence (*F_V_*), maximum fluorescence (*F_M_*), and maximum yield of primary photochemical efficiency (*F_V_/F_M_*) (Herritt et al., 2021). Modifications included two containers that converted BIN files to GeoTIFF images and clipped GeoTIFF images to plot boundaries using a GeoJSON file. The modification facilitated multi-modal data merging by acquiring geographical coordinates instead of pixel coordinates and enabled the integration of the software into the distributed computing framework. The PSII chlorophyll fluorescence pipeline consists of four components (**Supplementary Table 3** and **Supplementary Figure 3**). The first container converted 101 BIN files to 101 GeoTIFFs with approximate GPS bounding coordinates calculated from barcode positioning information contained within the associated JSON metadata file. The second container clipped GeoTIFFs to plot boundaries specified within a GeoJSON file. The third container segmented each pixel within an image into one of five *F_M_* experimentally derived contribution thresholds (Herritt et al., 2021). The fourth container applied the contribution thresholds to extract *F_0_* and *F_M_* values for each image pixel, which were used to calculate *F_V_* and *F_V_/F_M_* for each stack of 101 images were calculated as follows:


(9) $$F_{V}\;=F_{M}-F_{0}$$

(10) $$F_{V}/F_{M}\;=\;\frac{\left(F_{M}-F_{0}\right)}{F_{M}}$$

##### 2.3.2.4 3D laser scanner processing pipeline

**Figure 3:**
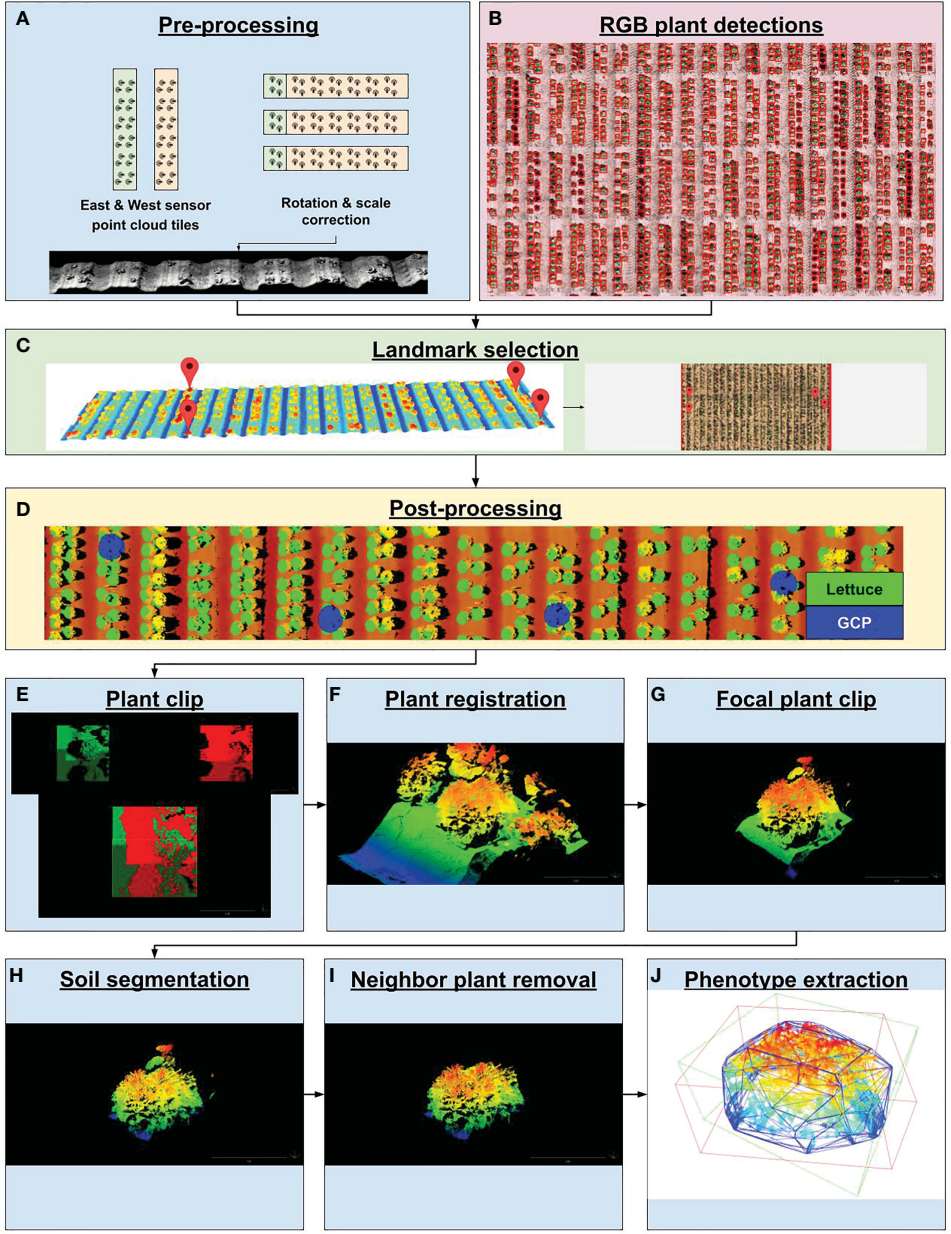
PhytoOracle 3D point cloud processing workflow. **(A)** Two raw point cloudscollected simultaneously were rotated, scaled, and georeferenced using positioning information from the Field Scanalyzer (FS) metadata file. **(B)** Time series plant detection from RGB data processing were coregistered with 3D point clouds by landmark selection. **(C)** Landmark selection involved selecting landmark features in point clouds and selecting the same landmark feature in RGB images. This step resulted in the co-registration of 3D point clouds with RGB data types. **(D)** Co-registered point clouds and plant detections were visualized by painting each plant detection with a green dot and ground control points (GCPs) with a blue dot. **(E)** Large point cloud tiles were clipped to known plant locations and **(F)** merged using the iterative closest point (ICP) algorithm. **(G)** Focal plants were further isolated by deploying a trained Faster R-CNN detection model to form a tight bounding box around the focal plant, eliminating neighboring plants. **(H)** Plant and soil points were segmented by deploying a trained DGCNN model on focal plant clips, resulting in a point cloud containing only plant points. **(I)** Residual neighbor plant points were removed by using the DBSCAN unsupervised clustering algorithm, resulting in a point cloud containing only focal plant points. **(J)** Focal plant point clouds were analyzed for morphometric phenotypes such as axis-aligned and oriented bounding box volumes (AABV and OBV, respectively) and convex hull volume (CHV), plant height (PH), and number of points (NP) as well as topological data analysis values calculated from persistence diagrams.


The 3D-FS datasets consist of 320 pairs of PLY files. A pair of structured-light laser scanners captured depth and reflectance imagery for preprocessing to point clouds, resulting in two PLY files per data capture (640 total PLY files). Pre-processing of image data to point clouds was performed by the manufacturer-provided software PlyWorker before the data was transmitted offsite. The pair of scanners captured the 3D structure of plants from east and west directions, thereby minimizing occlusions. Each pair of PLY files had an associated JSON metadata file. The 3D laser scanner pipeline, utilizing the output of the PlyWorker software as an input, consisted of six components (**Supplementary Table 3** and **Supplementary Figure 3**). The first container corrected the orientation and scale of the point cloud tiles and applied the RANSAC algorithm implemented in the Open3D Python package (v. 0.11.2) to find a simple translation (X and Y axes) to reduce misalignment (Fischler and Bolles, 1981; Choi et al., 2015; Zhou et al., 2018; Zhou et al., 2018) (**Figure 3A**). The second step co-aligned 3D point clouds to RGB-derived plant detections. A custom graphical user interface (GUI) was developed to download and visualize 3D point cloud data and RGB orthomosaic data on local computers after selecting a scan date to manually georeference (see Code Availability Statement). The purpose of this tool was to co-align 3D and RGB by identifying shared landmark features between 3D point clouds and RGB data. This co-alignment allows for individual plant clipping using RGB-derived plant detections (**Figure 3B**). Selected features included plot stakes, ground control point (GCP) lids, or distinguishable plants in the field. The GUI (*i*) shows the RGB orthomosaic region, (*ii*) prompts the user to select a landmark feature, (*iii)* displays the point cloud tile region that neighbors the selected landmark feature, (*iv*) prompts the user to select the corresponding landmark feature within the point cloud tile. This process is repeated until an adequate number of landmark features are selected (**Figure 3C**). After RGB and 3D data are co-registered by the user, an affine transformation is calculated from the correspondences between the selected landmark features. This transformation maps a point in the original space of the 3D point cloud into the space of the georeferenced RGB orthomosaic. This transformation was then saved to a JSON file. The third container applied the calculated transformation to the point cloud tiles, resulting in co-aligned, georeferenced point cloud tiles (**Figure 3D**). The fourth container used RGB-derived plant detections to clip individual plants from large point clouds tiles (**Figure 3E**). The fifth container merged multiple tiles containing the same plant using the iterative closest point (ICP) method implemented in the Open3D Python package (v. 0.11.2) (Besl and McKay, 1992; Zhou et al., 2018) (**Figure 3F**). The sixth container deployed a Faster R-CNN model to localize the focal plant on 3D-derived heat map images (**Figure 3G**). The seventh container segmented soil and plant points, which allowed for the isolation of plant points within each point cloud (**Figure 3H**). The eighth container removed any residual neighbor plant points using the DBSCAN clustering algorithm implemented in the Open3D Python package (v. 0.11.2) (Ester et al., 1996; Zhou et al., 2018) (**Figure 3I**). Lastly, the ninth container created persistence diagrams for a single plant point cloud using the Giotto-tda Python package (v. 0.5.1) (Tauzin et al., 2021), from which the following topological data analysis (TDA) values were collected: persistence entropy and amplitude (with distance functions of landscape, bottleneck, Wasserstein, Betti, silhouette, heat, and persistence image). Plant height (PH) was calculated as follows:

(11) $$\text{Plant height }=\;Z_{max}\;-\;Z_{min}$$

Where *Z_max_* is the maximum Z-axis plant point value and *Z_min_* is the minimum Z-axis plant point value. In addition, the oriented bounding box volume (OBV), axis-aligned bounding box volume (AABV), and number of points (NP) were calculated using the Open3D Python package (v. 0.11.2) (Zhou et al., 2018) (**Figure 3J**).

#### 2.3.3 Pipeline benchmarking

The RGB, thermal, and PSII pipelines were benchmarked using a single data collection for each sensor (**Table 2**). Benchmarking consisted of manager and worker compute nodes using CCTools Makeflow and Work Queue (Albrecht et al., 2012). A single HPC compute node equipped with two AMD Zen2 processors x 48 cores (94 total cores), 512 GB of RAM, sixteen 32 GB memory DIMM, and 2 TB SSD disk served as the manager node. Worker nodes, with the same computational resources mentioned above, were requested on which the command *work_queue_factory* (CCTools v. 7.1.12) was run to request one worker per core, resulting in a total of 94 Work Queue workers per node each with 5 GB of RAM. A Makeflow file containing information for each data input file was created programmatically using the PO automation script, which allowed for parallel distribution of tasks. In addition, this automation script provided a detailed workflow to each worker, specifying the processing step to be performed on each input file using Singularity v3.6 for running containers (Hunt and Larus, 2007; Kurtzer et al., 2017). A single task was performed per worker to allow for maximum distribution of tasks. Importantly, each pipeline differs in its definition of a single task input: RGB and thermal consist of one BIN file; 3D of two PLY files; and PSII of 101 BIN files, each with an associated metadata JSON file. Upon completion of assigned tasks, the manager compute node assigned additional tasks in queue to available workers. The benchmark dataset for RGB, thermal, and PSII sensors was processed over the following range of available workers: 1, 4, 8, 16, 32, 64, 128, 256, 512, and 1024. Each configuration was replicated three times, for a total of 30 benchmark data points per sensor. A log file with information on processing times and number of workers during processing was collected during processing.

**Table 2:** Information on each benchmarking dataset’s collection date, size, and number of images.

Sensor	Date	Start time	End time	Elapsed time	Total size	Image count
RGB	03/03/2020	08:45	13:27	04:42	140.7	9270
Thermal	03/03/2020	08:45	13:27	04:42	5.4	9270
PSII	02/27/2020	19:58	00:37	04:39	86.2	39678
3D laser	03/01/2020	18:59	03:54	08:55	308.5	640

Elapsed time, HH : MM; total size, gigabytes.

#### 2.3.4 Multi-modal data merging and association

To allow for identification of single plants throughout the growing period and across sensor modalities, individual plant detections from each collection date need to be grouped. Two phases were carried out to accomplish this: (*i*) data cleaning to remove any outliers and (*ii*) a series of sequential clustering steps to combine multi-modal datasets and enable individual plant tracking.

##### 2.3.4.1 Removal of outlier plants

The first phase involved the removal of overlapping plants, hereafter termed outliers, which were the result of two or more plants growing in proximity and merging into what appeared to be a single plant. These outliers resulted in a single plant detection for this pair of plants, leading to errors in subsequent analyses. To remove these outliers, the field was manually assessed at the end of the season for outliers, which were manually marked with spray paint for easy visual identification in imagery collected right before harvesting. A GeoJSON vector layer containing a point for each outlier was manually created on QGIS (www.qgis.org) and the end-of-season orthomosaic containing the marked outlier canopies, which were used to identify these outliers in the multi-modal dataset.

##### 2.3.4.2 Grouping plant phenotypes for individual plant tracking

The second phase involved the sequential clustering of phenotypic trait data from various sensor modalities. First, the full season RGB dataset was combined with the GeoJSON file containing manually marked outlier plant points generated in the first phase. Individual plant detections throughout the season were then clustered using agglomerative clustering, a form of hierarchical clustering algorithm implemented in the scikit-learn Python package v0.24.2 (Cox, 1957; Fisher, 1958; Ward, 1963; Pedregosa et al., 2011). Agglomerative clustering requires a threshold value, which was empirically derived based on having the lowest number of outliers grouped into a cluster and reduced fluctuations in growth curves. The optimal threshold value of 6 x 10^-7^ was used to maximize the number of clustered observations of a single plant and minimize the clustering of weeds and/or neighboring plants. The full season RGB plant detections were clustered using the empirically derived threshold value and results were assessed in QGIS. Each cluster, representing a single plant time series, was given a unique identifier denoting the plant’s genotype and the clustering number (“genotype”_”cluster number”). All clusters containing an outlier point were given the label ‘double’ for the identification and exclusion of these data points from subsequent analyses (**Supplementary Figure 4**). Second, the resulting grouped RGB dataset was then clustered with the full season thermal data. Full season RGB and thermal outputs were merged using the same technique used during clustering of the full season RGB data. This clustering step resulted in a single dataset containing RGB and thermal data with a shared unique plant identified. Third, the merged dataset, containing clustered RGB and thermal phenotypic trait data, was combined with PSII chlorophyll fluorescence and 3D laser full season files using plot numbers and unique plant identifiers, respectively. The final output was a time-series, multi-modal phenotypic trait dataset at the individual plant level for RGB, thermal, and 3D phenotype data and plot level for PSII chlorophyll fluorescence phenotype data.

#### 2.3.5 Analysis of extracted phenotypes

##### 2.3.5.1 Assessing accuracy of plant detection across growing period

To assess plant detection performance, the median IoU throughout various time points were quantified for RGB and thermal image data. Canopy temperature extraction performance was assessed by manually extracting median canopy temperature across all time points of a random sample of 200 selected plots, with each plot containing a minimum of five plants across 19 collection dates resulting in 1,481 data points. We examined the correlation between manually extracted canopy temperature and pipeline extracted MEDT over an entire season for these selected plots. The BA extraction performance was evaluated by assessing its Pearson correlation with harvested, fresh weight biomass for each plot in the field trial. The median individual plant BA was used for correlation assessments. A similar assessment of correlation was conducted for AABV extraction.

##### 2.3.5.2 Assessing grouping of plant phenotypes performance

The results from the proposed clustering association method were visualized across 200 plots as a vector layer overlaid on an end-of-season orthomosaic in which the outliers were marked. Each plot was imaged over 19 time points, resulting in a total of 3,800 images. If an identification was marked and the overlaid detection was identified as an outlier by the clustering algorithm, then the identification was classified as a true positive (TP). If the plant was marked and the overlaid detection was not determined to be an outlier by the clustering script, then the identification was classified as a false negative (FN). If the plant was not marked and the overlaid detection was determined to be an outlier by the clustering script, then the overlaid identification was classified as a false positive (FP). If the plant was not marked and the overlaid detection was determined to not be an outlier by the clustering script, then the overlaid identification was classified as a true negative (TN).

##### 2.3.5.3 Statistical analysis and data visualization

The BA, NP, OBV, AABV, and PH phenotype trait data were analyzed after first checking for residual normality and error variance homogeneity at each collection event. For each trait, collection time points were analyzed separately using the lme4 package (Bates et al., 2015) in the R programming language (R Core Team, 2022). Spatial effects were modeled on a row and column basis. The following linear mixed model was fitted to trait data for the estimation of variance components:


(12) $$y_{ijk}\;=\;\mu \;+\;g_{i}\;+\;irg_{j}+\;g\;\times \;irg_{ij}\;+\;rep{\left(irg\right)}_{kj}\;+\;row{\left(rep\times irg\right)}_{lkj}\;+\;col{\left(rep\times irr\right)}_{mkj}\;+\;\epsilon_{ijklm}$$

where *y_ijk_* is an individual phenotypic observation; *μ* is the overall mean; *g_i_* is the effect of the *i*-th genotype; *irg_j_* is the effect of the *j*-th irrigation treatment which was either WW, D1 or D2; *g* × *irg_ij_* is the interaction effect between the *i*-th genotype and the *j*-th irrigation treatment; *rep*(*irg*)*_kj_* is the effect of the *k*-th replication nested within the *j*-th irrigation treatment; *row*(*rep* × *irg*)*_lkj_* is the effect of the *l*-th plot grid row nested with *k*-th replication within the *j*-th irrigation treatment; *col*(*rep* × *irr*)*_mkj_* is the effect of the *m*-th plot grid column nested within the *k*-th replication within the *j*-th irrigation treatment; and *ε_ijklm_* is the residual effect. The variance component estimates from the full model were used to estimate repeatability (*r*) as follows:


(13) $$r\;=\;\frac{\sigma_{g}^{2}}{\sigma_{g}^{2}\;+\;\frac{\sigma_{gi}^{2}}{n_{irg}}\;+\;\frac{\sigma_{\in }^{2}}{n_{plot}}}$$

where $\sigma_{g}^{2}$ is the genotypic variance due to genotypes, $\sigma_{gi}^{2}$ is the estimated variance with the genotype-by-irrigation treatment variation, and $\sigma_{\in }^{2}$ and residual variances, respectively. The variable *n_irg_* is the number of irrigation treatments in which each genotype was observed and *n_plot_* is the number of plots in which the genotype was observed. 

All plots presented in this study were generated using the Seaborn, Matplotlib, and Plotly Python packages using Python v3.9 (Hunter, 2007; Hossain, 2019; Waskom, 2021). Pearson correlations presented in the plots were calculated using the SciPy Python package (v0.15.1) (Virtanen et al., 2020).

## 3 Results

### 3.1 Environmental conditions during growing period

Weather data mean values for the growing season between 2019-11-13 and 2020-03-03 were: 10.72 °C air temperature, 61.88% relative humidity, 0.62 kPa vapor pressure deficit, and 0.55 MJ/m^2^ solar radiation (**Supplementary Figure 5**). The irrigation treatments resulted in contrasting VSWC, with minimum values at 10 cm of 19.2, 14.7, and 12.8 in irrigation treatments WW, D1, and D2, respectively. At 30 cm, minimum values were 21.3, 21.5, and 17.2 for WW, D1, and D2, respectively (**Supplementary Figure 1**).

### 3.2 Model performance metrics

Faster R-CNN models were separately trained to identify single plants in RGB and thermal imagery, each trained and evaluated with 2,000 and 250 images, respectively. Performance was assessed without any prediction confidence threshold, resulting in 2,752 and 1,450 ‘plant’ class detections for RGB and thermal, respectively. The RGB detection model detected plants with a 0.98 recall, 0.93 precision, 0.96 F1-score, and 0.96 overall accuracy when tested on FS (RGB-FS) image data. The RGB detection model performance was further evaluated with a 400-image RGB-DR test dataset and resulted in 0.98 recall, 0.96 precision, 0.97 F1-score, and 0.97 overall accuracy. The thermal detection model performed better than the RGB detection model with a 0.98 recall, 0.99 precision, 0.98 F1-score, and 0.98 overall accuracy. A single DGCNN model was trained to segment points corresponding to plant and soil classes in point clouds containing a single plant. The model was trained and evaluated with 128 point clouds and 16 point clouds, respectively. The DGCNN model was assessed for point-wise accuracy using the test set, which was calculated at 0.98 (**Table 3**).

**Table 3:** Performance metrics for Faster R-CNN detection models for image processing of Field Scanalyzer RGB (RGB-FS), drone RGB (RGB-DR), and Field Scanalyzer thermal (Thermal-FS).

Model	Data	Type	Detections	TP	FP	FN	Recall	Precision	F1-score	Accuracy
A	RGB - DR	Detection	4356	4097	182	77	0.98	0.96	0.97	0.97
A	RGB - FS	Detection	2752	2519	178	54	0.98	0.93	0.96	0.96
B	Thermal - FS	Detection	1450	1404	10	36	0.98	0.99	0.98	0.98
C	3D - FS	Segmentation	–	–	–	–	–	–	–	0.98

FS, Field Scanalyzer; DR, drone; TP, true positive; FP, false positive; and FN, false negative. For the 3D-FS model, the accuracy reported is a point-wise accuracy collected across points within the test dataset, as such values for columns Total detections through F1-score are not presented.

**Figure 4:**
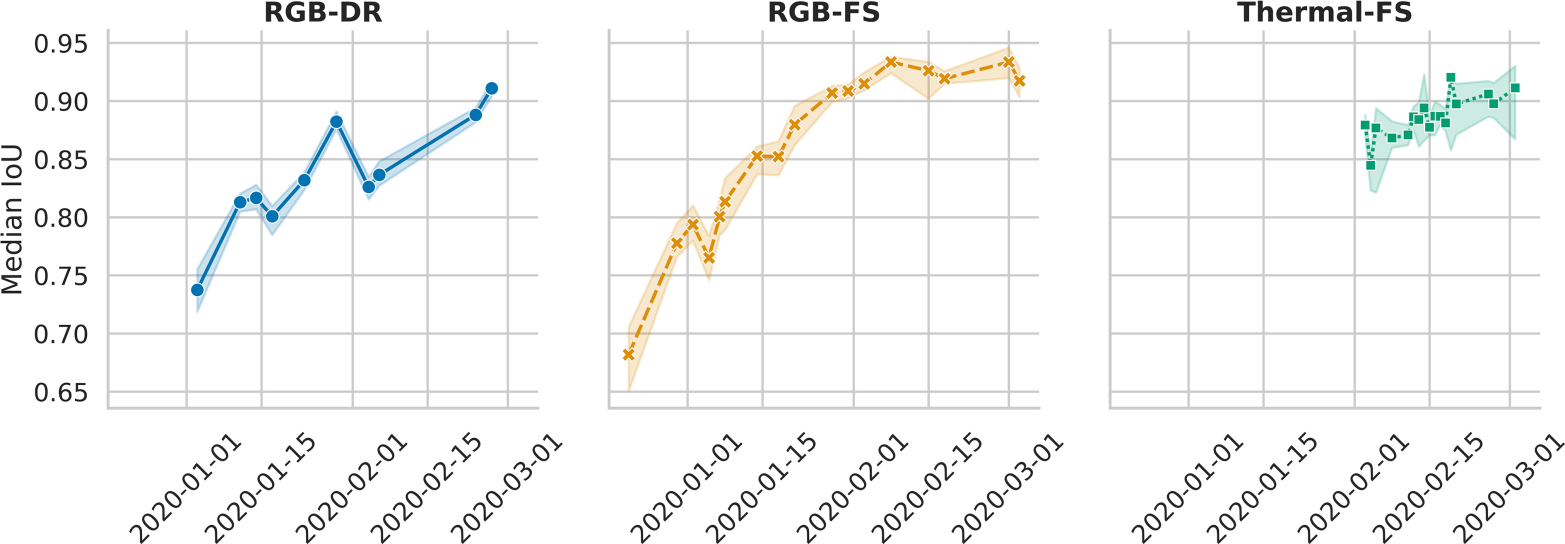
Change in median Intersection over Union (IoU) across the collection dates represented in RGB and thermal test data sets for the Field Scanalizer (FS) and Drone (DR) systems. Both RGB Field Scanalyzer scans (RGB-FS) and drone flights (RGB-DR), began earlier than thermal, allowing to capture the temporal effect of collection date, a proxy to plant size, on the median IoU. Error bands represent 95% confidence intervals around the median.

The median IoU was calculated separately for each distinct collection time point represented in a 250-image test set to assess temporal effects on bounding box accuracy. Overall, the median IoU was 0.84, 0.84, and 0.88 for RGB-FS, RGB-DR, and thermal-FS, respectively. The median IoU differed between dates, with an increasing trend as time progressed (**Figure 4**). This trend was stronger in the RGB-FS and RGB-DR data as these data were collected earlier in the season when plants were small with fewer distinguishable features as compared to thermal scans.

### 3.3 Validation of pipeline-extracted phenotypes and multimodal data association

**Figure 5:**
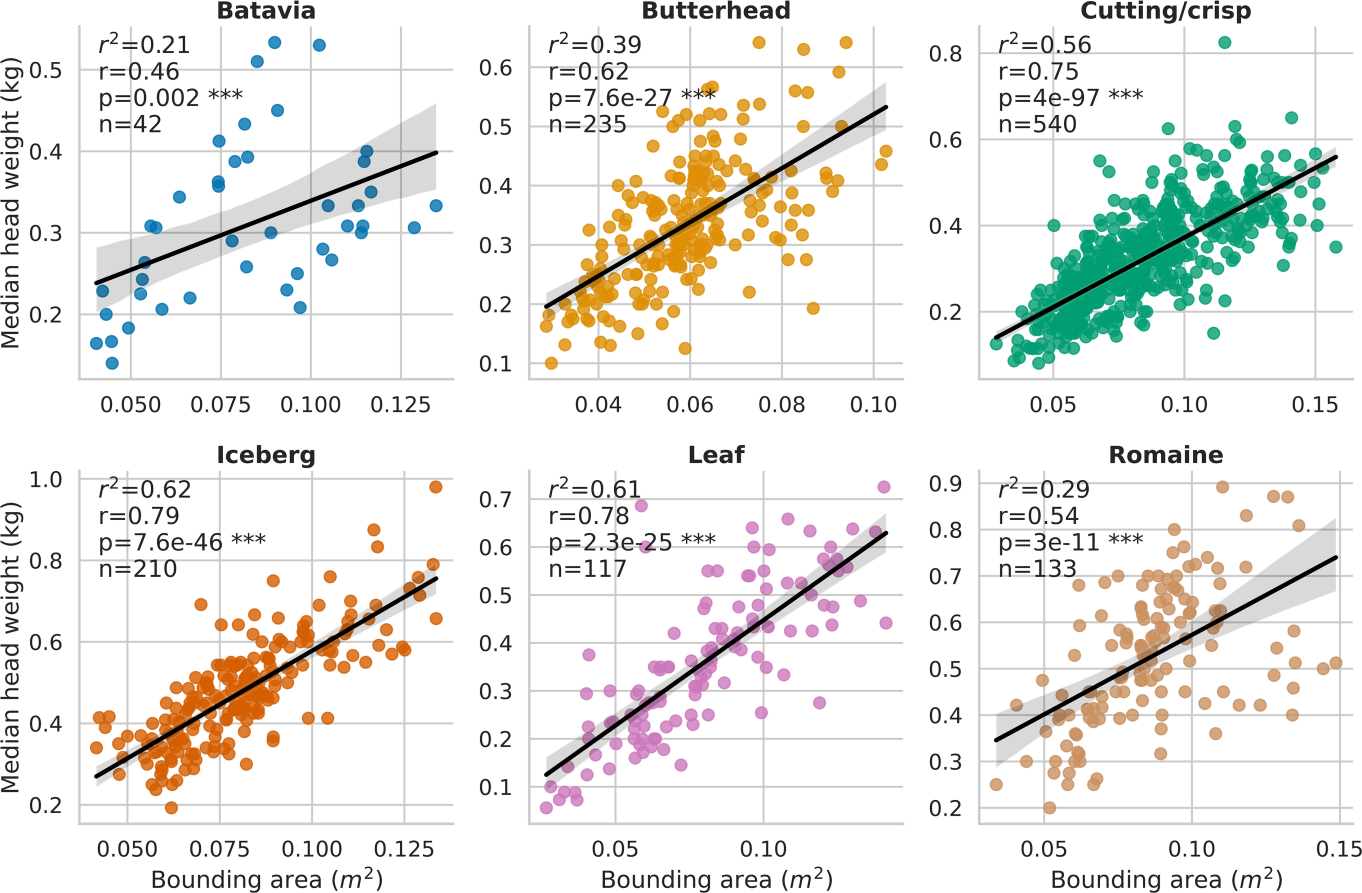
Correlation between individual plant fresh weight and pipeline-extracted bounding area (RGB-FS BA, m^2^) for all plots in the field trial. Genotypes were grouped by horticultural type, resulting in 6 groups which are Batavia, Butterhead, Cutting/crisp, Iceberg, Leaf, and Romaine. *** = P value ≤ 0.001.

**Figure 6:**
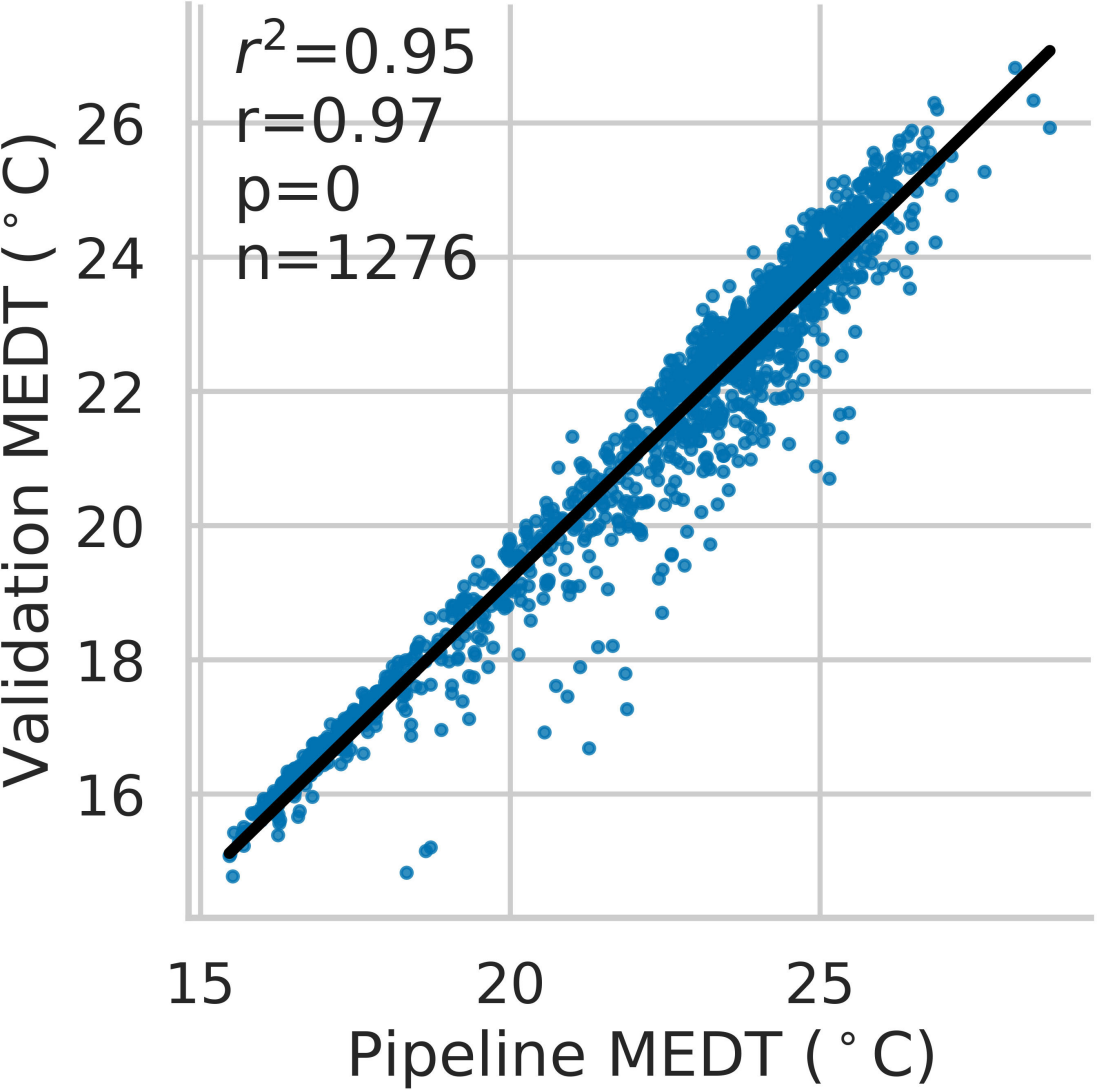
Correlation between validation and pipeline-extracted median canopy temperatures (MEDT). Each point represents an individual plant temperature collected at a single time point, with the complete dataset consisting of 12 distinct collection dates.

Across the entire time series clustering test set, the agglomerative clustering method grouped plant detections into individual plant, time-series data with 0.99 recall, 0.93 precision, 0.96 F1-score, and 0.96 overall accuracy. The observed coefficient of determination (*r* ^2^) between individual plant fresh weight collected at harvest and pipeline-extracted 3D-FS AABV were 0.29 for Batavia (p< 0.01), 0.36 for Butterhead (p< 0.0001), 0.55 for Cutting/Crisp (p< 0.0001), 0.59 for Iceberg (p< 0.0001), 0.61 for Leaf (p< 0.0001), and 0.48 for Romaine (p< 0.0001) (**Supplementary Figure 6**). The observed coefficient of determination (*r* ^2^) between individual plant fresh weight and pipeline-extracted RGB-FS BA were 0.21 for Batavia (p< 0.01), 0.39 for Butterhead (p< 0.0001), 0.56 for Cutting/Crisp (p< 0.0001), 0.62 for Iceberg (p< 0.0001), 0.61 for Leaf (p< 0.0001), and 0.29 for Romaine (p< 0.0001) (**Figure 5**). The observed range of *r* ^2^ values between manually extracted and pipeline-extracted median canopy temperatures (MEDT) over 12 unique collection dates was 0.43-0.94 (**Supplementary Figure 7**). The overall observed *r* ^2^ was 0.95 when considering all dates (p< 0.0001) (**Figure 6**).

### 3.4 Collection and processing benchmarks

#### 3.4.1 Field scanalyzer data collection

Benchmark datasets were collected using the FS, with varying operation times depending on the sensor. The file size of benchmark datasets ranged from 5.4 GB to 308.5 GB in size and consisted of 640 to 39,678 files. The data collection of RGB and thermal image data, which occurs simultaneously, took a total of 4 hours and 42 minutes to complete resulting in 9,270 raw images per sensor. The PSII data collection took 4 hours and 39 minutes, resulting in the largest raw file count (39, 678 images).

#### 3.4.2 PhytoOracle data processing

**Figure 7:**
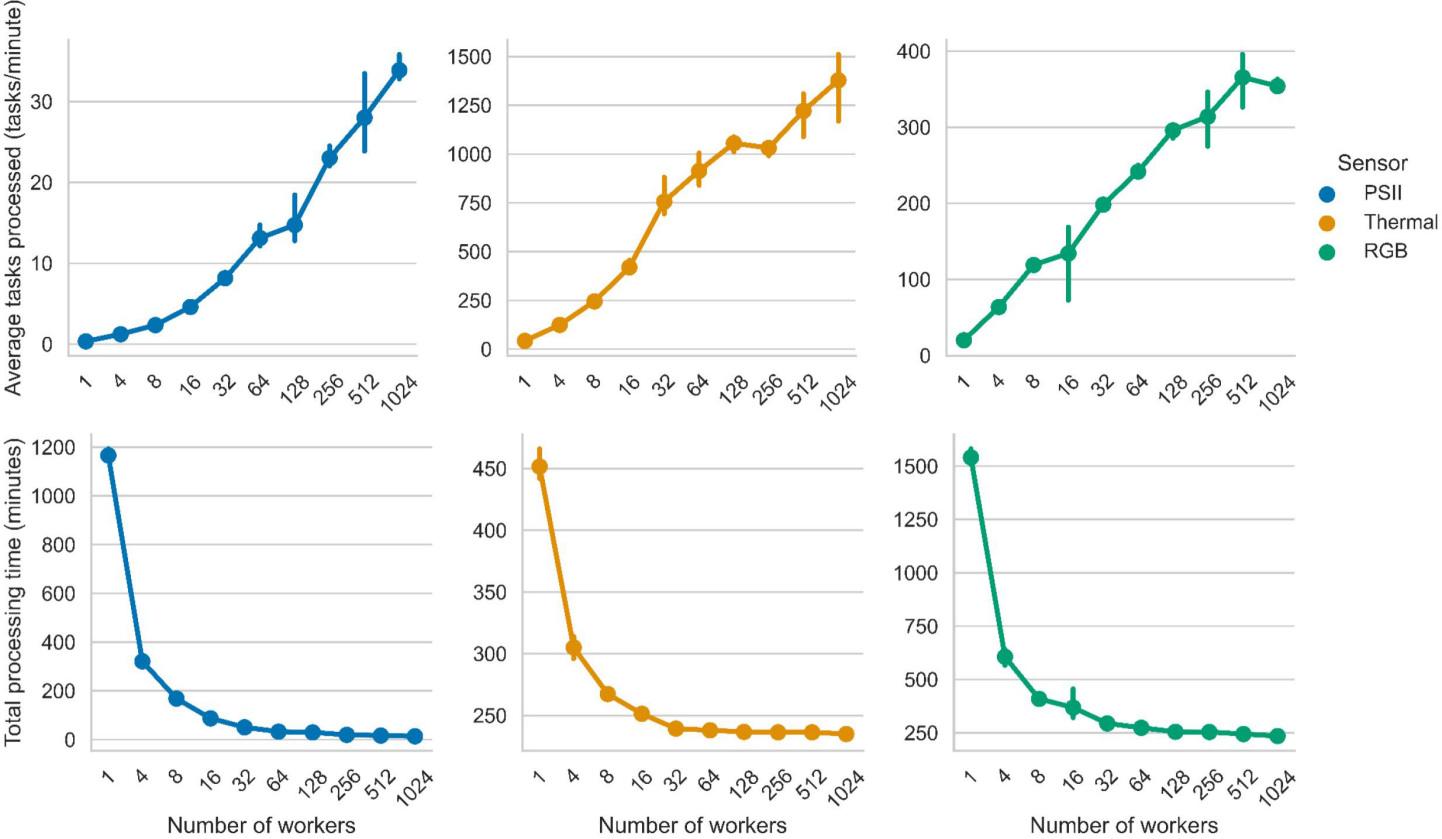
Average tasks process per minute and processing times for each PhytoOracle pipeline. (Top) Average tasks processed (tasks/minute) as a function of the number of worker cores. (Bottom) Total processing time (minutes) as a function of the number of workers (one CPU core per work). Available workers ranged from 1 to 1024 and the values represent the average of three runs with the same configuration. Error bars represent 95% confidence intervals.

The RGB and PSII processing times saw the largest reduction from computational parallelization, at 61% and 95% respectively, at the maximum number of 1024 workers. Thermal processing time saw the smallest reduction of 22% at the maximum number of 1024 workers. At the maximum number of workers tested in this study, RGB and thermal each processed in 235 minutes and PSII in 13 minutes (**Figure 7**).

### 3.5 Phenotypic repeatability estimates at individual sampling events

**Figure 8:**
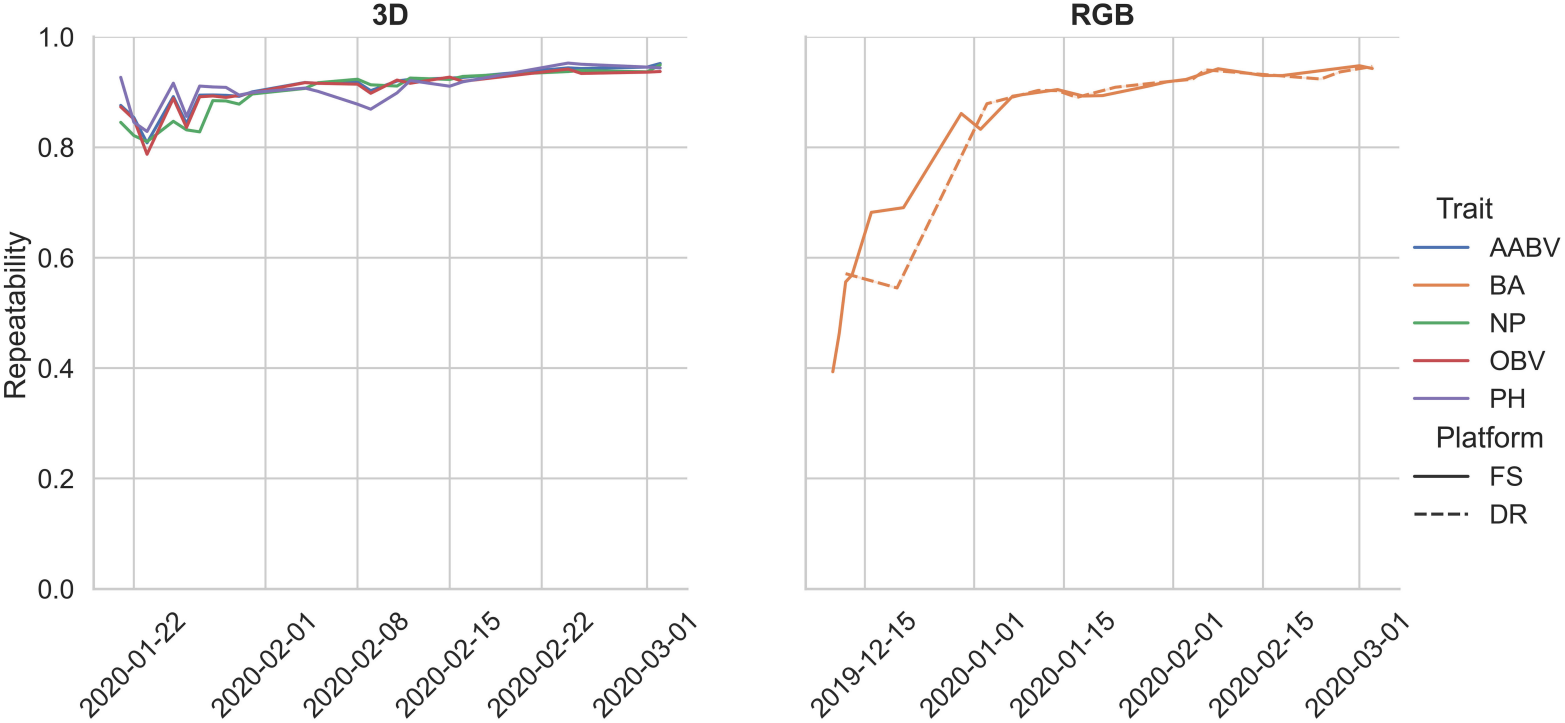
Repeatability estimates for pipeline-extracted phenotypes collected during a single year trial. Bounding area, BA; axis-aligned bounding box volume, AABV; number of points, NP; oriented bounding box volume, OBV; plant height, PH; Field Scanalyzer, FS; drone, DR.

The mean repeatability values for each pipeline are as follows: 0.86 (RGB-DR BA), 0.81 (RGB-FS BA), 0.90 (3D-FS AABV), 0.90 (3D-FS OBV), 0.90 (3D-FS PH), and 0.89 (3D-FS NP) (**Table 4**). In general, the repeatability of RGB and 3D phenotypic trait data had increasing trends over the growing season (**Figure 8**).

**Table 4:** Repeatability of pipeline extracted phenotypes collected from Field Scanalyzer (FS) and drone (DR) platforms.

Data	Trait	Min.	Mean	Max.
RGB-DR	Bounding area	0.55	0.86	0.95
RGB-FS	Bounding area	0.39	0.81	0.95
3D-FS	Axis-aligned bounding volume	0.81	0.90	0.95
3D-FS	Oriented bounding volume	0.79	0.90	0.94
3D-FS	Plant height	0.83	0.90	0.95
3D-FS	Number of points	0.81	0.89	0.95

Minimum, Min.; Max., Maximum.

## 4 Discussion

The proliferation of phenomics technology has led to large data volumes that need to be processed. Challenges related to computation of phenomics big data reduce its full application and efficacy in providing actionable genome-phenome insights into plant morphophysiological traits. Among the significant bottlenecks in plant phenomics, we address the lack of scalable, modular processing pipelines capable of processing expanding data volumes to extract morphological and physiological phenotypic trait data. Although other pipelines, such as Image Harvest and Greenotyper, have considered and implemented distributed computing systems, these capabilities have not been fully developed for general use on HPC clusters or multiple node deployment. Instead, it is left to the user to undertake that implementation (Knecht et al., 2016; Tausen et al., 2020). The PhytoOracle suite of scalable, modular data processing pipelines addresses critical bottlenecks within plant phenomics including data diversity, scalability, reproducibility, and extensibility. PhytoOracle accomplishes this by integrating distributed computing, container technology, data management systems, and machine learning into a single suite of phenomics data processing pipelines.

### 4.1 PhytoOracle addresses neglected bottlenecks in phenomics data processing

The PO suite can process data from multiple sensors including RGB, thermal, and PSII chlorophyll fluorescence 2D image data and 3D point cloud data. Except for PSII chlorophyll fluorescence, PO data processing pipelines result in individual plant phenotypic trait data that can be associated using our agglomerative clustering approach (**Figure 2** and **Supplementary Figure 5**). To date, the only other published pipeline capable of handling such diverse data types is PlantCV. However, PlantCV’s approach to individual plant phenotyping does not translate well to field phenomics data (Fahlgren et al., 2015; Gehan et al., 2017). In field phenomics data, plant spacing creates challenges for individual plant phenotype extraction. The threshold-based contour approach used by much software, including PlantCV, works well in controlled environments, however, most imaging approaches outside of controlled environments often capture multiple overlapping plants under highly variable lighting conditions. These variable conditions make threshold-based contour approaches difficult to implement in processing field phenomics data. For this reason, PO leverages ML models that are better able to handle overlapping plants and variable lighting conditions.

To resolve time series, multi-plant measurements to the individual plant level, PO leverages ML approaches, such as Faster R-CNN for object detection and DGCNN for point cloud segmentation. These ML models make PO robust and generalizable to other crops. For instance, if a user wants to process a new crop species, a model could be trained and deployed within PO, requiring little to no code development. Furthermore, the ML models presented here can be used by other researchers and/or new models can be trained using our labeled data and existing containers. PO also provides a general use solution to training of Faster R-CNN object detection models.

The PO suite provides scalability through a distributed computing framework leveraging the open-source CCTools’ Makeflow and Work Queue software (Albrecht et al., 2012), which provides the language and computational resource management necessary to scale tasks beyond traditional job arrays and local computing resources. Importantly, this enables users to leverage dataset-specific resources across multiple computing environments during data processing, providing a path to maximize and optimize computational resource use. For example, the manager can be launched on an HPC cluster to ensure adequate storage space while workers could be launched on a lab workstation. The benefit of this approach is that computational resources beyond one computer or even one cluster can be leveraged to process thousands of tasks in parallel. Data processing on a single computer or server constrains users to locally available memory and processors, preventing scalability. On the other hand, distributed computing systems allow users to access processors and memory on remote nodes, allowing the system to, in theory, linearly scale the processing task at hand. The PO benchmarking focused on HPC nodes instead of local nodes and cloud-native options, such as XSEDE, due to those resources not having the storage space required to store raw and intermediate data. This is important, as it highlights that computational resources must consider not only CPU/GPU availability but also storage space capabilities as large-scale phenomics data processing results in many intermediate outputs that must be temporarily stored to serve as input to subsequent steps. In the end, these intermediate data can be deleted, but they must be able to be temporarily stored during data processing.

As data volumes increase, scalability will become a higher priority within research fields aimed at extracting relevant insights from big data (Chen et al., 2013; Sivarajah et al., 2017). However, this is likely to exacerbate existing network IO bottlenecks, which prevent linear scaling (Zhang et al., 2020). For example, the presented benchmarking information shows that although the average number of tasks completed continued to increase, the total processing time remained relatively stable after 32 workers. These results highlight limitations in scaling likely associated with network and data transfer bottlenecks. Improving the utilization of local, cloud, or HPC systems is a major concern and area of active research (Tanash et al., 2019). Generally, there are seemingly two options for further improvements to computational throughput: (*i*) identifying the optimal worker configurations per pipeline and/or (*ii*) moving pipelines closer to where the data are collected. An analysis of big data environments using Docker containers found that adding nodes (workers) beyond a certain threshold decreased performance due to an increase in the time for a network request to be sent and received (round trip time), which is similar to the results presented here (China Venkanna Varma et al., 2016). Moving pipelines closer to the data seems more feasible than finding optimal worker configurations as there may not be an optimal worker configuration to mitigate scaling plateaus until network bottlenecks are resolved. Network bandwidth is commonly associated with a lack of linear scaling; oftentimes, the processing phase is efficient and would theoretically allow for linear scaling, but the communication phase creates a bottleneck preventing linear scaling (Zhang et al., 2020). In our case, raw data is stored on the CyVerse Data Store due to its volume, velocity, and variety–making it intractable to keep these data on local servers for processing. This results in data being located “far” (CyVerse Data Store servers) from the processing pipeline (HPC), resulting in significant network requests that negatively impact data processing throughput. In the future, improvements to network capabilities may help to further improve processing efficiency.

The PO suite leverages container technology to ensure consistent, immutable data processing. Each PO processing step is containerized using Docker and deployable on HPC, cloud, and local computers on which either Docker or Singularity is installed. As opposed to running non-containerized processing code, containers ensure that each processing step is reproducible by controlling code versions and processing environments. Instead of users having to install over 40 Python packages to run PO, we provide containers that contain these libraries, significantly reducing the barrier to entry (**Supplementary Table 4**). Additionally, the PO automation script automatically downloads and configures CCTools, and requires no additional third-party Python packages. The only requirements for running PO are Singularity or Docker, iRODS, and Python. These tools are generally found on HPC clusters, except for iRODS which can be installed by system administrators.

The PO suite provides a general use framework through our automation script. Together with our suite of processing containers, this automation script automates the complexity of developing a PCSs, allowing users with little computer programming experience to leverage PO for processing their own phenomics data. The PO suite has four existing YAML files that can be customized by other researchers to process their own data. Users with advanced programming and command line experience can develop their own containers for data processing and integrate them into PO by including each container as a module within the YAML file, specifying the location of raw data on the CyVerse Data Store or local storage, and outlining the expected output files. The use of a generalizable automation script and a customizable YAML file makes it possible for users to run PO on various datasets, allowing researchers to spend more time on analysis than software development and data processing.

### 4.2 PhytoOracle extracts repeatable phenotypes from distinct platforms

The phenotypic trait data extracted from the FS and DR platforms align with values reported in the literature. Morphological trait repeatability values collected by the 3D-FS sensor align with the range of values reported in wheat (Deery et al., 2019; Walter et al., 2019; Deery et al., 2020). Similar values for 3D-FS phenotypes are reported here: 0.81-0.95 (AABV), 0.79-0.94 (OBV), 0.83-0.95 (PH), and 0.81-0.95 (NP). These values highlight the usefulness and applicability of PO for phenotype extraction, particularly morphological phenotypes. Additionally, similar trends of repeatability values were found across two distinct datasets: 0.55-0.95 and 0.39-0.95 for RGB-DR and RGB-FS platforms, respectively. These overlapping repeatability values demonstrate the applicability of PO to multiple platforms. The lower limit for repeatability for bounding area is an artifact of varying data collection start dates: 2019-12-10 for RGB-FS, 2019-12-12 for RGB-DR, and 2020-01-21 for 3D-FS. These earlier dates had a greater number of plants per plot, lowering the ability to accurately extract individual plant phenotypes due to overlap between plants. The number of plants per plot was reduced to approximately ten on 2020-01-16. Notably, all 3D-FS scans were collected after this date, resulting in a narrower range of repeatability values due to all scans being collected on well-spaced, lower overlap conditions.

Repeatability is dependent on data and algorithms, meaning that any system could result in similar repeatability values as PO. However, an important difference is the ease at which these other systems handle and process large volumes of data to extract those repeatable phenotypic trait values. The PO system addresses this issue by allowing the extraction of highly repeatable traits in a few hours. Furthermore, the PO system also provides extensibility. Each module within PO collectively results in highly repeatable phenotypic traits across sensor data types. Even in cases where scalability is not necessary, such as small volumes of drone data, these repeatability values across sensors and phenotyping platforms highlight PO’s wide range of applications. The PO system, therefore, accelerates data processing of diverse data types from across phenotyping platforms, enabling the extraction of highly repeatable phenotypic traits that would otherwise have to be extracted using various, disparate systems or software that make it difficult to analyze, interpret, or combine resulting outputs.

### 4.3 PO enables deployment of future algorithms across species

The PO suite addresses challenges in scalability and modularity to improve plant phenomics data processing. This was accomplished by leveraging existing and emerging technologies to process large volumes of phenomics data in a scalable, modular manner. Existing technologies include container technology, distributed computing frameworks, and data management systems, while emerging technologies include ML models for trait extraction. By coordinating this combination of technologies, PO processes data in an automated, efficient manner across platforms and sensors. The PO suite serves as a tool for others in plant phenomics to leverage within their research groups. This is made possible by the diverse availability of processing containers which can be deployed on any system on which Docker, Singularity, iRODS, and CCTools are installed. The phenotypic data processed by PO show high repeatability values across platforms, indicating PO’s utility within plant science and plant breeding programs. Importantly, the PO suite provides large volumes of phenotypic trait data that can be combined with other -omics data for applications in selection, dissection of functional and adaptive traits, and characterization of temporal patterns in trait expression (**Supplementary Figure 8**).

As ML methods mature, new models can be implemented within PO due to its customizable YAML configuration file. For example, models for leaf segmentation and extraction of traits such as leaf curling at scale, are the next steps of PO development. Furthermore, the training of these models is possible due to the large volume of intermediate data generated by pipelines like PO, which can serve as (*i*) training data for these next-generation models and (*ii*) as samples for model-generated data to further increase training data sizes. Containers that deploy these next-generation ML models could then be added to existing PO pipelines to provide organ-level phenotypic trait data that complements existing whole plant phenotypic trait data. This volume and diversity of phenomics data would enable fine-scale phenotyping at scale, which may uncover details on the temporal patterns in trait expression.

PhytoOracle addresses many phenomics bottlenecks, but there are outstanding bottlenecks such as enviromic capabilities and multi-species support. Enviromic capabilities are limited within PO, which are important to account for the environmental noise encountered in field phenomics data. In the future, PO pipelines will be further developed to output environmental data directly from the Field Scanalyzer and neighboring weather stations alongside phenotypic trait data. As this would be difficult to generalize across users, we decided not to provide this capability at present. However, the authors understand that these complementing data would enhance interpretability and interoperability of processed phenotypic trait data, therefore, we plan to support these capabilities in the future. Although the present study focuses on lettuce, PO has been refactored to process sorghum phenomics data with the same containers used to process lettuce phenomics data (**Supplementary Figure 9**). Further research and development will lead to the extraction of species-specific traits, and it is our goal to publish updates on these added functionalities.

## 5 Conclusion

The scalable, modular PhytoOracle data processing pipelines enable the extraction of large, time-series phenotypic trait data in an automated and reproducible manner, key factors required to process projected data volumes. The resulting traits extracted by PO from both FS and DR platforms show high repeatability, highlighting the usefulness of PO across phenotyping platforms. The intermediate processed data, such as individual plant point clouds, extracted by PO opens new opportunities to extract fine-scale phenotypes at multiple resolutions (plot, plant, and organ levels). Importantly, the PO pipelines can be refactored to process phenomics data from other crops species, as discussed here with sorghum phenomics data. In the future, these time-series datasets may provide biological insight into morphological and physiological responses to drought conditions at the individual plant level across multiple crop species. This information could enable new species-specific targets for genetic improvement based on time-series, fine-scale phenotypic trait data.

## Code availability statement

The Python scripts used to prepare RGB training data can be accessed here: http://github.com/phytooracle/automation/blob/main/ml/collect_rgb_data.py. The Python script used to prepare thermal training data can be accessed here: http://github.com/phytooracle/automation/blob/main/ml/collect_flir_data.py. The Python script used to prepare 3D-derived images can be found here: http://github.com/phytooracle/3d_heat_map/blob/main/3d_heat_map.py. The code used to train object detection models can be found here: http://github.com/phytooracle/ezobde. Examples of YAML files used for data processing can be accessed here: http://github.com/phytooracle/automation/tree/main/yaml_files. The automation script and data processing repositories can be accessed at: http://github.com/phytooracle. Each PhytoOracle container built from data processing repositories can be accessed at: http://hub.docker.com/orgs/phytooracle. For a detailed description of each data processing3repository and associated container, refer to the **Supplementary Material**.

## Data availability statement

The datasets presented in this study can be found in online repositories. The names of the repository/repositories and accession number(s) can be found below: https://datacommons.cyverse.org/browse/iplant/home/shared/phytooracle/season_10_lettuce_yr_2020.

## Author contributions

EMG conceptualized and developed processing code, analyzed data, and wrote the manuscript. AriZ, NH, TS conceptualized and developed processing code, and contributed to manuscript preparation. ArmZ, MC conceptualized and developed processing code. JD conceptualized and developed code related to data acquisition, oversaw the Field Scanalyzer operation, and contributed to manuscript preparation. HE contributed to the development of processing code and oversaw data labeling. SD conceptualized and developed processing code related to data transfer from Field Scanalyzer to the CyVerse Data Store. DL and MT contributed to the field design, harvesting, and manual collection of ground truth data. RM, TLS, NM, and EL contributed to project conceptualization, overseeing processing code development, and manuscript preparation. DP conceptualized, designed, and oversaw all aspects of the project including acquisition of funds and manuscript preparation. All authors contributed to the review of the manuscript and all authors have read the manuscript.
