# Supplementary material for: PhytoOracle: Scalable, modular phenomics data processing pipelines
Source: Front Plant Sci. 2023 Mar 6;14:1112973. doi: 10.3389/fpls.2023.1112973 (PMC10025408; doi:10.3389/fpls.2023.1112973)
Supplement: Supplementary file 1 [file DataSheet_1.docx]

***Supplementary Material***

# Supplementary Figures and Tables

## Supplementary Figures


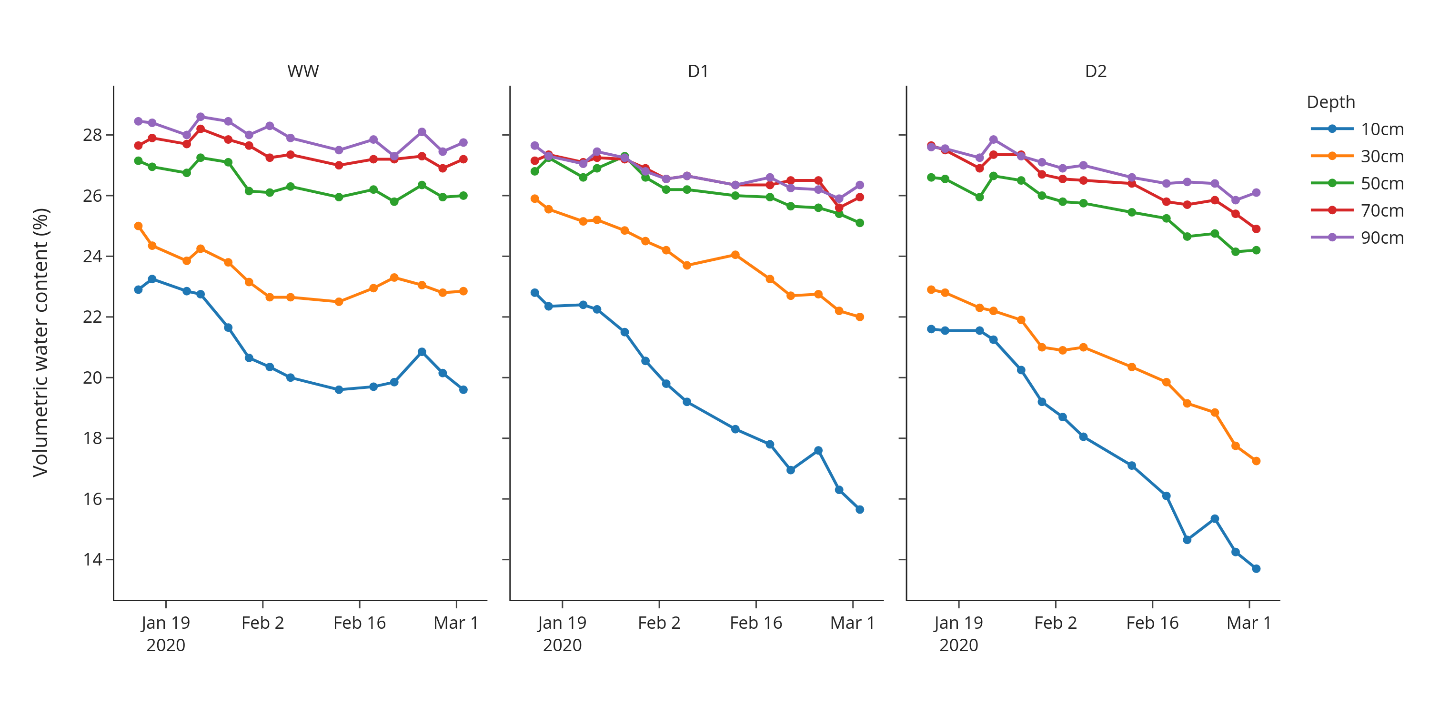


[**Supplementary**](https://docs.google.com/document/d/19unckO2ft1iM9kvz3rwsQ40vIb8asShd/edit#bookmark=id.gjdgxs) **Figure 1.** Neutron tube readings of volumetric soil water content (%) throughout the season 10 lettuce growing period. Readings were collected at 10, 30, 50, 70, and 90 cm depths. WW=well watered, D1=level 1 drought (75% of WW), D2=level 2 drought (50% of WW).


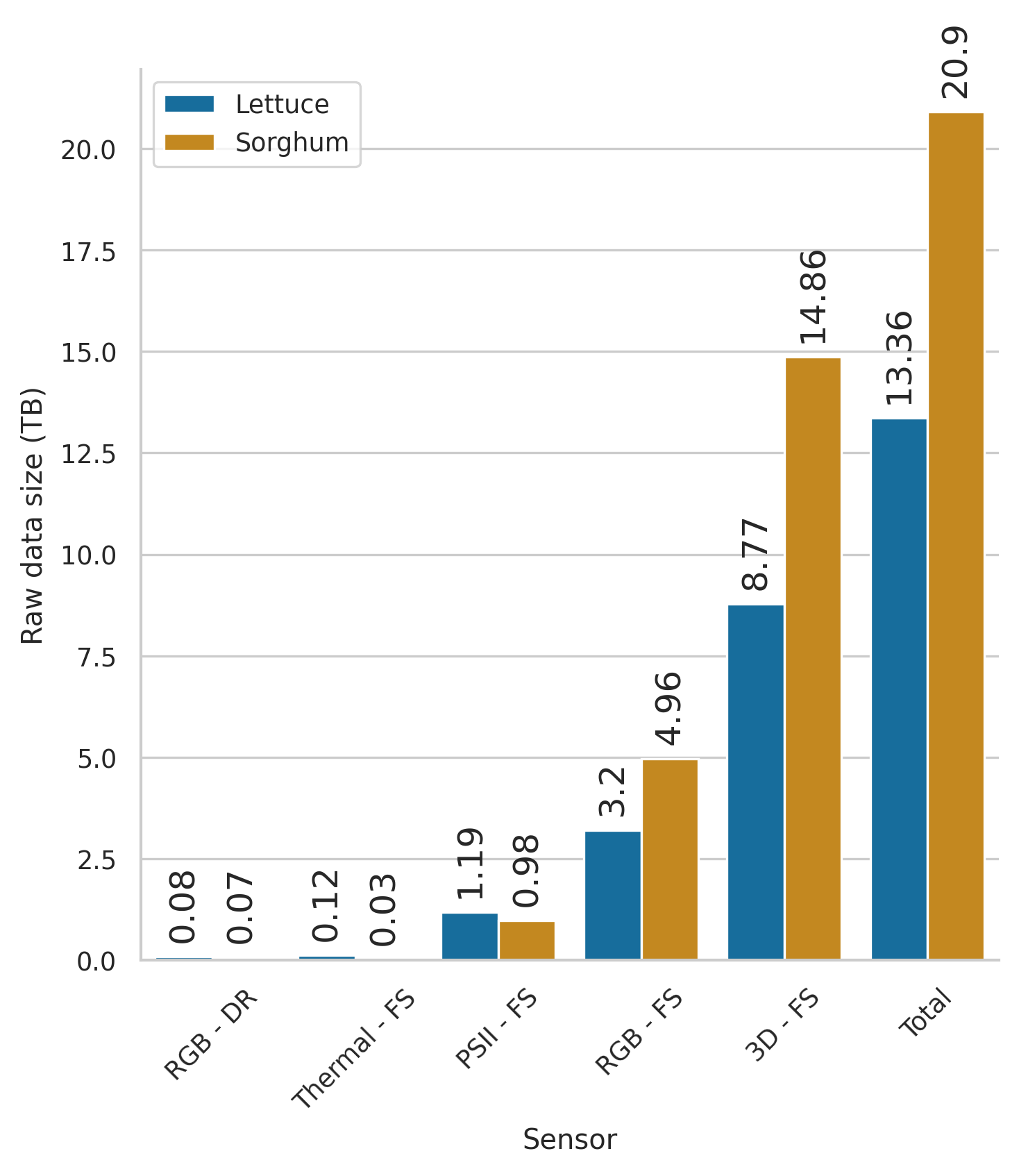


[**Supplementary Figure 2**](https://docs.google.com/document/d/19unckO2ft1iM9kvz3rwsQ40vIb8asShd/edit#bookmark=id.30j0zll)**.** Field Scanalyzer (FS) and drone (DR) raw data sizes in terabytes (TBs) for lettuce and sorghum seasons. Blue bars represent lettuce, gold represent sorghum. The rightmost bar is the total for each species/season.


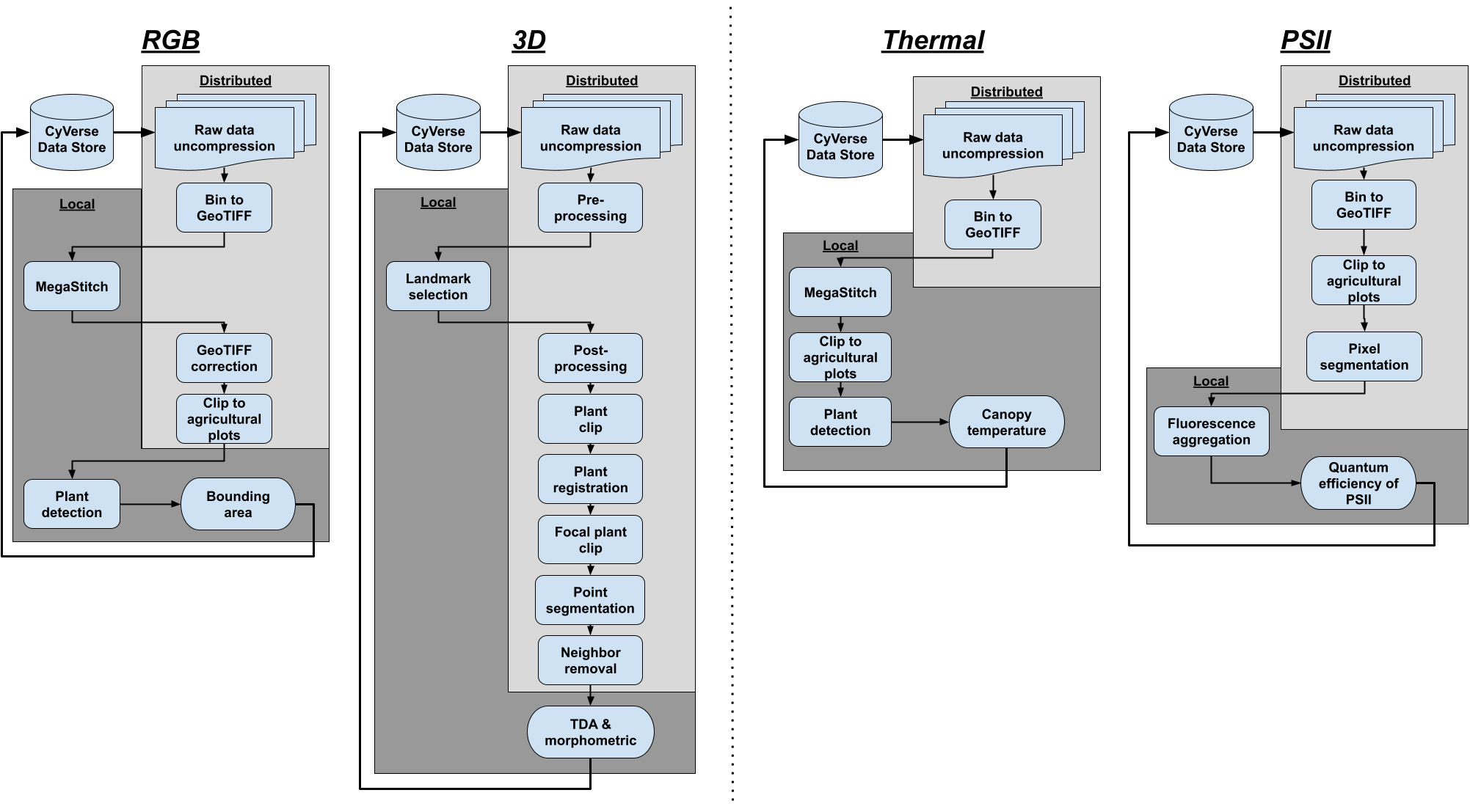


[**Supplementary Figure 3**](https://docs.google.com/document/d/19unckO2ft1iM9kvz3rwsQ40vIb8asShd/edit#bookmark=id.1fob9te)**.** PhytoOracle processing steps for RGB and 3D morphometric pipelines and thermal and PSII physiological pipelines.


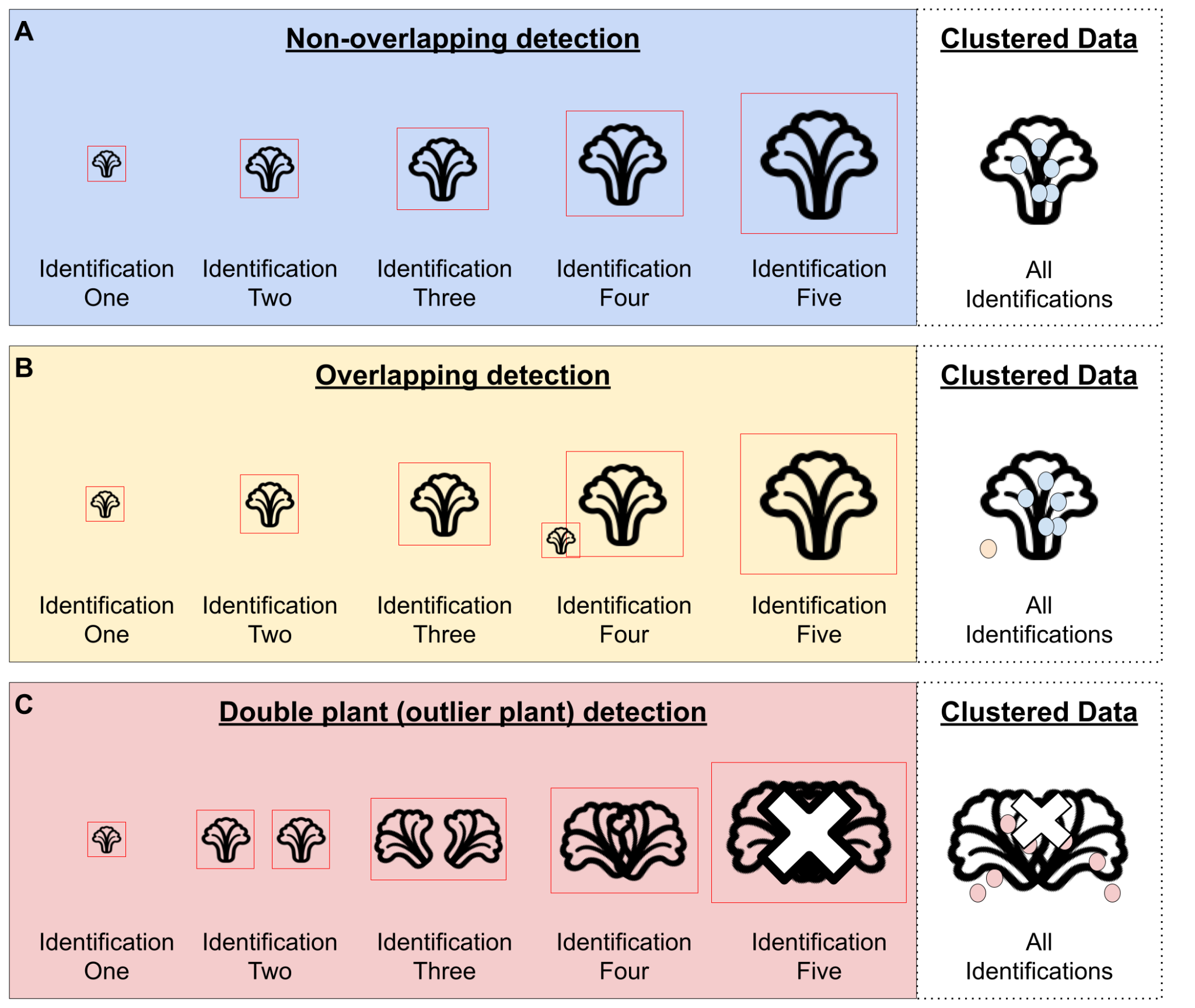


[**Supplementary Figure 4**](https://docs.google.com/document/d/19unckO2ft1iM9kvz3rwsQ40vIb8asShd/edit#bookmark=id.3znysh7)**.** Agglomerative clustering of time series data for single plant detections across RGB and Thermal sensor data. (A) Non-overlapping plant detections were clustered and associated with a given plant name. (B) When plant identifications overlapped, clustering resulted in the exclusion of the overlapping, non-focal plant identification with a different plant name. (C) Outlier plants were removed using a GeoJSON file with coordinates for each outlier point collected at the end of the season.


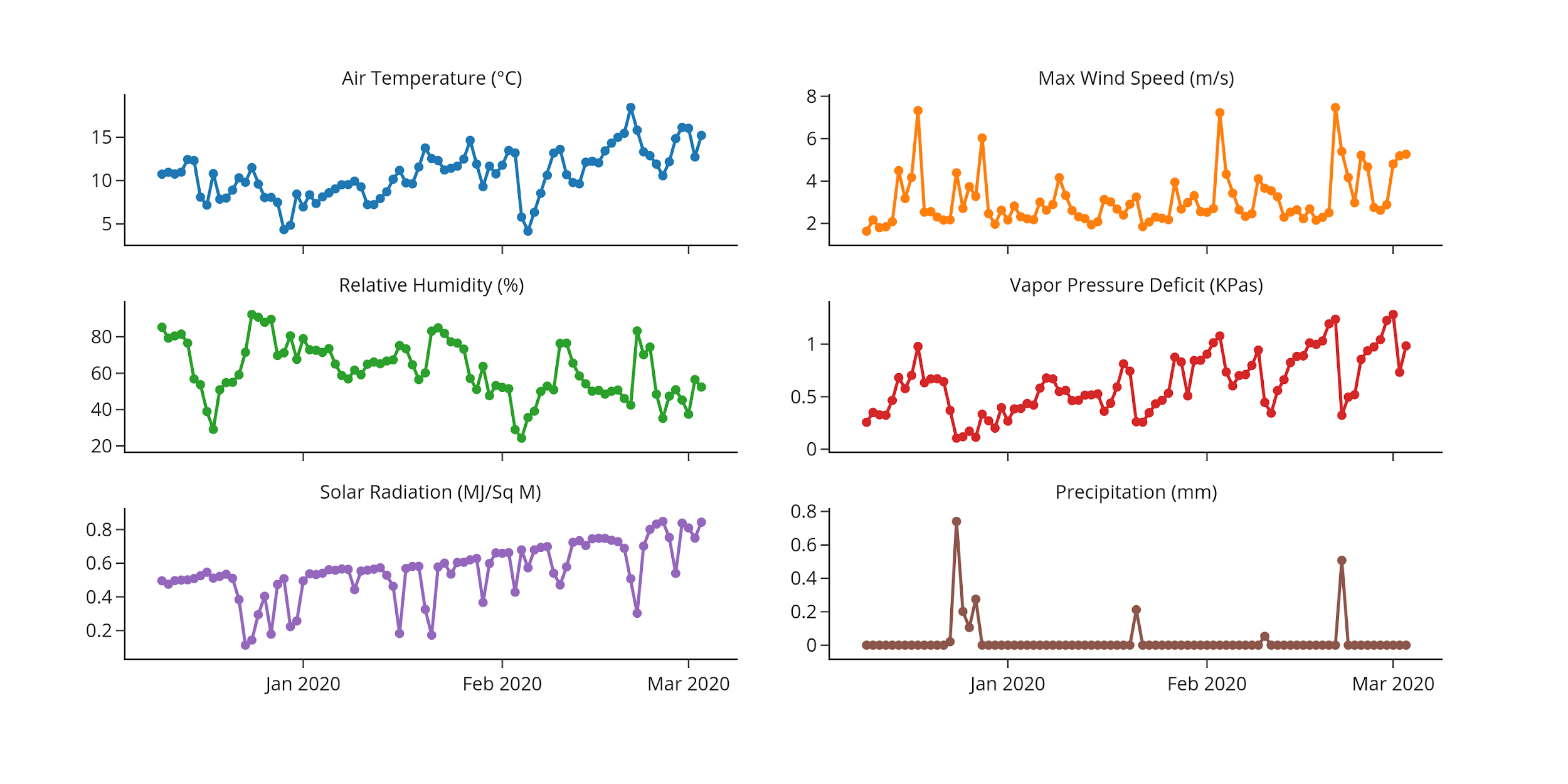


[**Supplementary**](https://docs.google.com/document/d/19unckO2ft1iM9kvz3rwsQ40vIb8asShd/edit#bookmark=id.2et92p0) **Figure 5.** Weather data throughout the lettuce growing season. Data was collected at the AZMET (Brown 1989) weather station in Maricopa, AZ (33.068941, -111.972244).

**
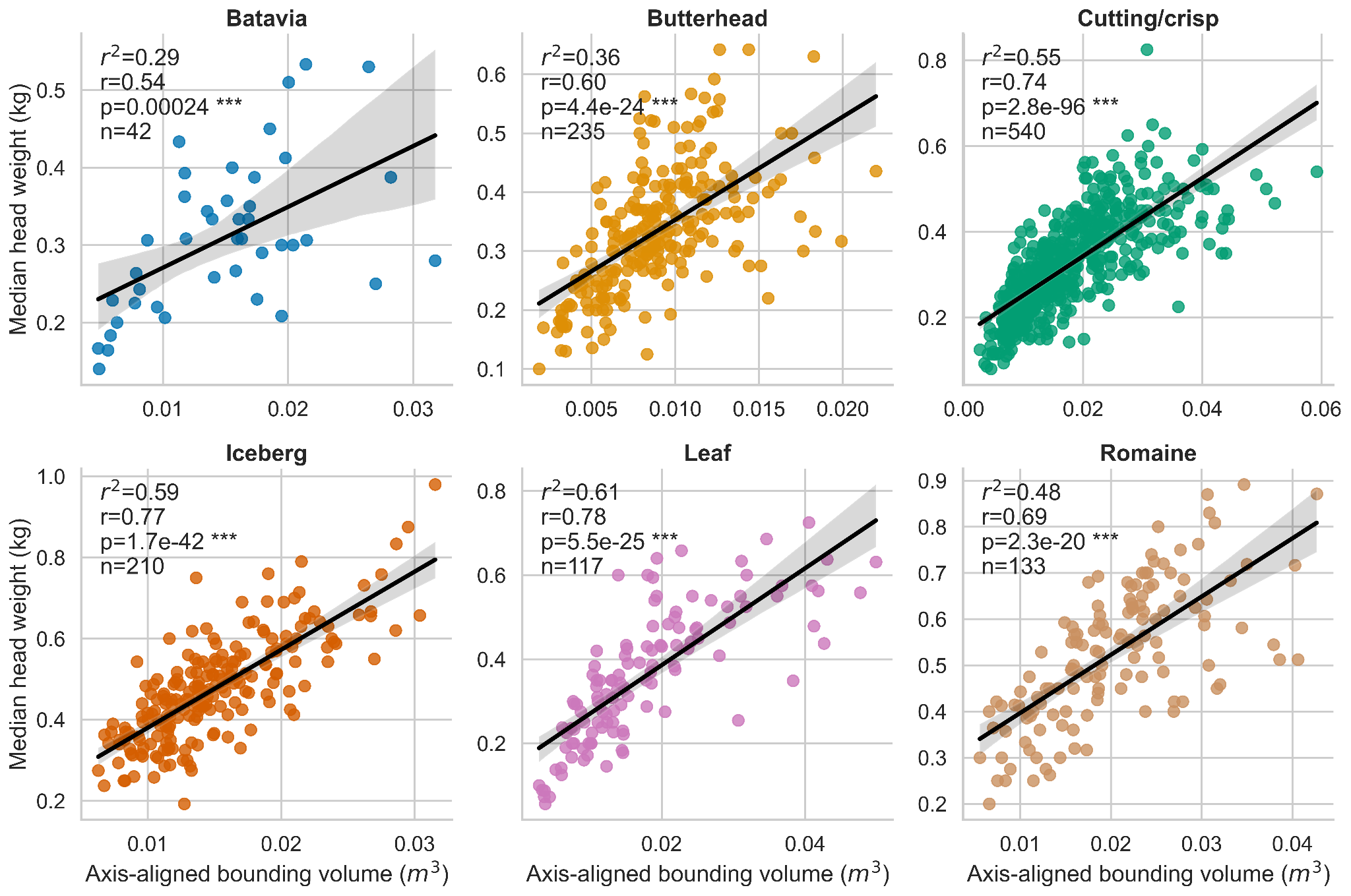
**

**Supplementary Figure 6.** Correlation between individual plant fresh weight and pipeline-extracted axis-aligned bounding volume (3D-FS AABV, $m^{3}$) for all plots in the field trial. Genotypes were grouped by horticultural type, resulting in six groups: Batavia, Butterhead, Cutting/Crisp, Iceberg, Leaf, and Romaine.


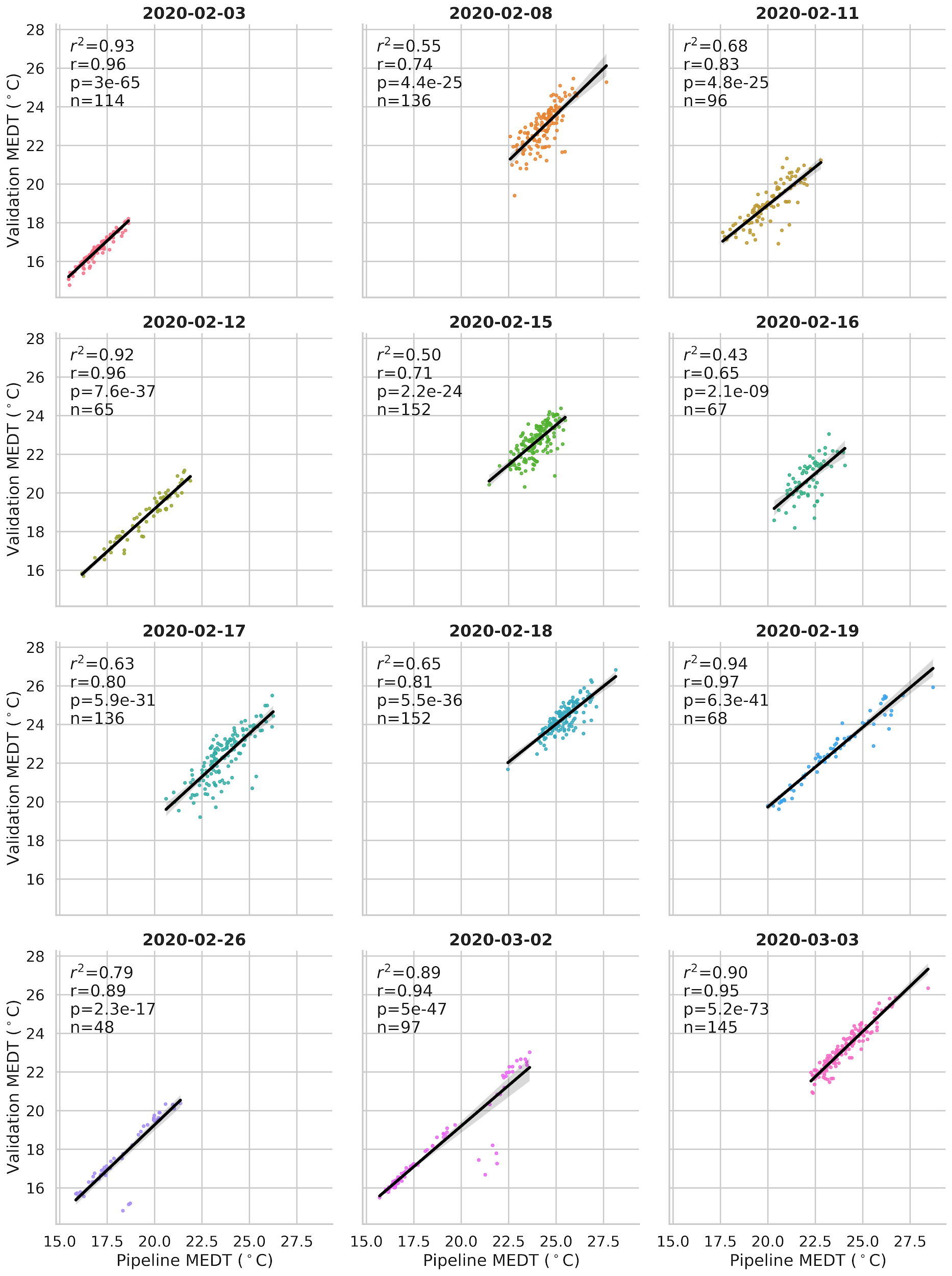


[**Supplementary**](https://docs.google.com/document/d/19unckO2ft1iM9kvz3rwsQ40vIb8asShd/edit#bookmark=id.tyjcwt) **Figure 7.** Correlation between manually extracted validation median canopy temperature and pipeline-extracted median canopy temperatures (MEDT) across 12 collection dates. Each point represents an individual plant temperature collected at a single time point.


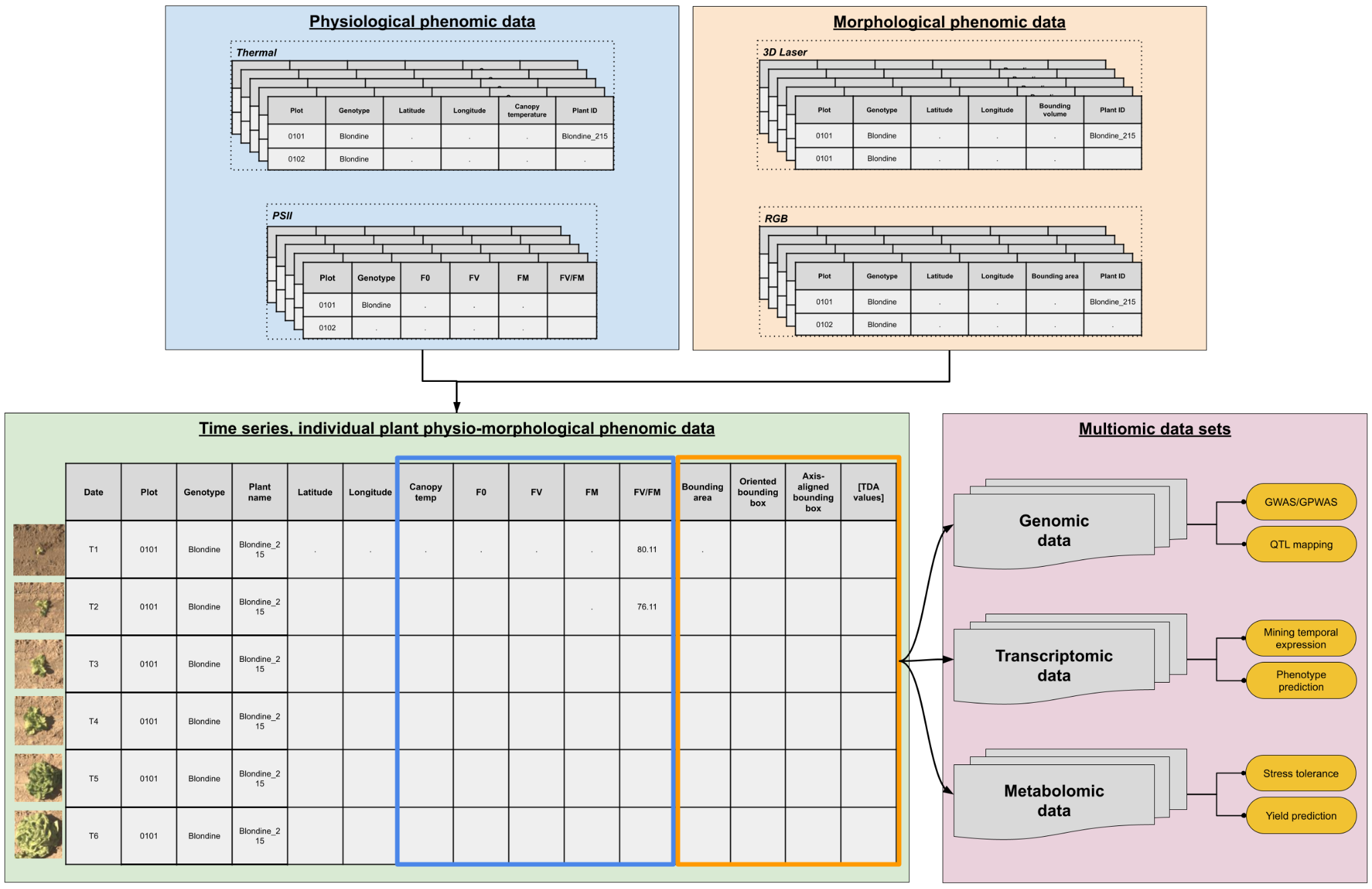


[**Supplementary Figure 8**](https://docs.google.com/document/d/19unckO2ft1iM9kvz3rwsQ40vIb8asShd/edit#bookmark=id.3dy6vkm)**.** PhytoOracle’s framework can be used as a starting framework to develop analysis pipelines for other omics data in addition to phenomic data. When processed efficiently, these multiomic data sets can be used for selection, identification of stress-adaptive traits, and capturing the temporal patterns of trait expression.


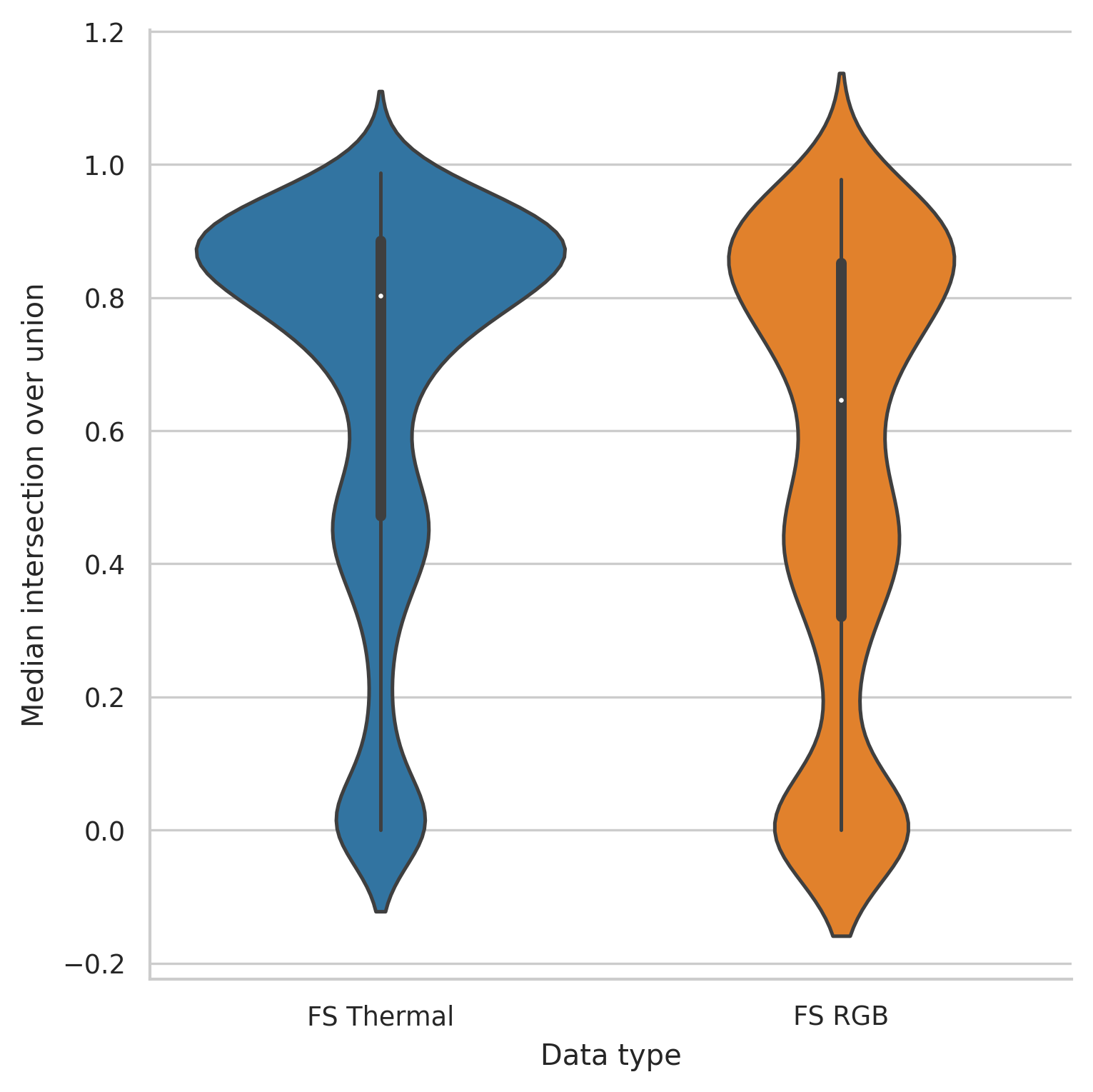


[**Supplementary Figure 9**](https://docs.google.com/document/d/19unckO2ft1iM9kvz3rwsQ40vIb8asShd/edit#bookmark=id.1t3h5sf)**.** Median intersection over union (IoU) for season 11 sorghum data. FS, Field Scanalyzer.

## Supplementary Tables

[**Supplementary Table 1**](https://docs.google.com/document/d/1b-jKFdFwnQ65CANT0oN1dD-hPOgK-ls4/edit#bookmark=id.gjdgxs)**.** Overview of Field Scanalyzer (FS) sensors supported by PhytoOracle. Width of image in N-S direction is provided for a 2 m working distance. Field of view and operating distance in units meters. PAS, point-and-shoot; Line, Line scanning; Y, yes; N, no.

| **Platform** | **Sensor Name** | **Model** | **Raw Format** | **Raw Type** | **Resolution** | **Field of View** | **Operating Distance** | **Output Type** | **PhytoOracle Support** |
| --- | --- | --- | --- | --- | --- | --- | --- | --- | --- |
| FS | Stereo RGB | Allied Vision Prosilica GT3300C | BIN | Image | 3296 x 2472 | 1.3 | 2 | Plot clipped & full field orthomosaics | Y |
| FS | Thermal | FLIR A615 | BIN | Image | 640 x 480 | 1.5 | 2 | Plot clipped & full field orthomosaics | Y |
| FS | PS II | LemnaTec PS II Fluorescence Prototype | BIN | Image | 1936 x 1216 | 1.1 | 0.8 | Plot clipped orthomosaics | Y |
| FS | 3D laser | Custom Fraunhofer laser scanner | PLY | Point Cloud | - | 0.85 | 3.5 | Individual plant point clouds | Y |
| FS | Visual-Near IR (VNIR) | Headwall HyperSpec Inspector | ENVI | Hyperspectral data cube | - | 1 | 2 | - | N |
| FS | Shortwave IR (SWIR) | Headwall HyperSpec Inspector | ENVI | Hyperspectral data cube | - | 0.75 | 2 | - | N |
| DR | RGB | DJI Phantom 4 Pro | JPEG | Image | 4096 × 2160 | - | 15 | Plot clipped & full field orthomosaics | Y |

[**Supplementary Table 2**](https://docs.google.com/document/d/1b-jKFdFwnQ65CANT0oN1dD-hPOgK-ls4/edit#bookmark=id.30j0zll)**.** Field Scanalyzer (FS) and drone (DR) data collection log for lettuce and sorghum seasons. For the lettuce season, RGB was listed as “Std (StVis/IR/NDVI/PRI/CO2/CC)” under the Sensor column as many sensors collected data concurrently. For the sorghum season, RGB was listed as StVis as the other sensors did not collect data concurrently.

| **Start time** | **Platform** | **Season** | **Sensor** | **Notes** | **Plant height** | **Scan height** | **Range lower bound** | **Range upper bound** | **End time** | **Scan duration** |
| --- | --- | --- | --- | --- | --- | --- | --- | --- | --- | --- |
| 2019-11-26 10:56:00 | DR | Season 10 Lettuce | DJI Phantom 4 Pro |  |  | 10 | 1 | 54 | 2019-11-26 11:18:00 | 0 days 00:22:00 |
| 2019-12-04 13:28:05 | FS | Season 10 Lettuce | Std (StVis/IR/NDVI/PRI/CO2/CC) | resumption of previous scan | 0 | 2 | 2 | 53 | 2019-12-04 14:04:52 | 0 days 00:36:47 |
| 2019-12-05 14:16:25 | FS | Season 10 Lettuce | Std (StVis/IR/NDVI/PRI/CO2/CC) | resumption of previous scan | 0 | 2 | 2 | 53 | 2019-12-05 14:27:18 | 0 days 00:10:53 |
| 2019-12-05 18:46:48 | FS | Season 10 Lettuce | 3D | south-to-north fullfield 3d | 0 | 3.5 | 2 | 53 | 2019-12-06 3:41:27 | 0 days 08:54:39 |
| 2019-12-06 19:15:34 | FS | Season 10 Lettuce | 3D | south-to-north fullfield 3d | 0 | 3.5 | 2 | 53 | 2019-12-07 4:08:05 | 0 days 08:52:31 |
| 2019-12-10 9:03:42 | FS | Season 10 Lettuce | Std (StVis/IR/NDVI/PRI/CO2/CC) | north-to-south fullfield standard/dense morning fog | 0 | 2 | 2 | 53 | 2019-12-10 13:44:11 | 0 days 04:40:29 |
| 2019-12-10 18:31:45 | FS | Season 10 Lettuce | 3D | south-to-north fullfield 3d | 0 | 3.5 | 2 | 53 | 2019-12-11 3:24:38 | 0 days 08:52:53 |
| 2019-12-11 9:00:59 | FS | Season 10 Lettuce | Std (StVis/IR/NDVI/PRI/CO2/CC) | north-to-south fullfield standard | 0 | 2 | 2 | 53 | 2019-12-11 13:41:08 | 0 days 04:40:09 |
| 2019-12-11 18:42:15 | FS | Season 10 Lettuce | 3D | south-to-north fullfield 3d | 0 | 3.5 | 2 | 53 | 2019-12-12 3:34:49 | 0 days 08:52:34 |
| 2019-12-12 8:51:11 | FS | Season 10 Lettuce | Std (StVis/IR/NDVI/PRI/CO2/CC) | north-to-south fullfield standard | 0 | 2 | 2 | 53 | 2019-12-12 13:37:15 | 0 days 04:46:04 |
| 2019-12-12 13:53:00 | DR | Season 10 Lettuce | DJI Phantom 4 Pro |  |  | 15 | 1 | 54 | 2019-12-12 14:06:00 | 0 days 00:13:00 |
| 2019-12-12 19:00:18 | FS | Season 10 Lettuce | 3D | south-to-north fullfield 3d | 0 | 3.5 | 2 | 53 | 2019-12-13 3:56:57 | 0 days 08:56:39 |
| 2019-12-13 9:01:54 | FS | Season 10 Lettuce | Std (StVis/IR/NDVI/PRI/CO2/CC) | north-to-south fullfield standard | 0 | 2 | 2 | 53 | 2019-12-13 13:42:11 | 0 days 04:40:17 |
| 2019-12-13 18:42:28 | FS | Season 10 Lettuce | 3D | south-to-north fullfield 3d | 0 | 3.5 | 2 | 53 | 2019-12-14 3:37:23 | 0 days 08:54:55 |
| 2019-12-16 9:20:46 | FS | Season 10 Lettuce | Std (StVis/IR/NDVI/PRI/CO2/CC) | north-to-south fullfield standard/FLIR dropped out approx 13:25 | 0 | 2 | 2 | 53 | 2019-12-16 14:00:55 | 0 days 04:40:09 |
| 2019-12-16 18:56:05 | FS | Season 10 Lettuce | 3D | south-to-north fullfield 3d | 0 | 3.5 | 2 | 53 | 2019-12-17 3:52:21 | 0 days 08:56:16 |
| 2019-12-18 18:36:01 | FS | Season 10 Lettuce | 3D | south-to-north fullfield 3d | 0 | 3.5 | 2 | 53 | 2019-12-19 3:37:32 | 0 days 09:01:31 |
| 2019-12-19 9:44:10 | FS | Season 10 Lettuce | Std (StVis/IR/NDVI/PRI/CO2/CC) | north-to-south 3ppr standard/startrange40 | 0 | 2 |  |  | 2019-12-19 13:25:29 | 0 days 03:41:19 |
| 2019-12-19 18:38:03 | FS | Season 10 Lettuce | 3D | south-to-north fullfield 3d | 0 | 3.5 | 2 | 53 | 2019-12-20 3:31:57 | 0 days 08:53:54 |
| 2019-12-20 11:46:00 | DR | Season 10 Lettuce | DJI Phantom 4 Pro |  |  | 15 | 1 | 54 | 2019-12-20 11:59:00 | 0 days 00:13:00 |
| 2019-12-20 12:06:00 | DR | Season 10 Lettuce | DJI Phantom 4 Pro |  |  | 10 | 1 | 54 | 2019-12-20 12:25:00 | 0 days 00:19:00 |
| 2019-12-20 19:09:07 | FS | Season 10 Lettuce | 3D | south-to-north fullfield 3d | 0 | 3.5 | 2 | 53 | 2019-12-21 4:03:20 | 0 days 08:54:13 |
| 2019-12-21 8:55:55 | FS | Season 10 Lettuce | Std (StVis/IR/NDVI/PRI/CO2/CC) | north-to-south fullfield standard | 0 | 2 | 2 | 53 | 2019-12-21 13:38:51 | 0 days 04:42:56 |
| 2019-12-21 19:08:39 | FS | Season 10 Lettuce | 3D | south-to-north fullfield 3d | 0 | 3.5 | 2 | 53 | 2019-12-22 4:03:19 | 0 days 08:54:40 |
| 2019-12-23 9:41:32 | FS | Season 10 Lettuce | Std (StVis/IR/NDVI/PRI/CO2/CC) | 3ppr standard north-to-south/startrange31 | 0 | 2 |  |  | 2019-12-23 13:28:00 | 0 days 03:46:28 |
| 2019-12-23 18:35:33 | FS | Season 10 Lettuce | 3D | fullfield 3D south-to-north | 0 | 3.5 | 2 | 53 | 2019-12-24 3:30:41 | 0 days 08:55:08 |
| 2019-12-24 13:32:24 | FS | Season 10 Lettuce | Std (StVis/IR/NDVI/PRI/CO2/CC) | resumption of previous scan | 0 | 2 |  |  | 2019-12-24 13:36:19 | 0 days 00:03:55 |
| 2019-12-25 18:11:01 | FS | Season 10 Lettuce | 3D | south-to-north fullfield 3d | 0 | 3.5 | 2 | 53 | 2019-12-26 3:05:50 | 0 days 08:54:49 |
| 2019-12-26 9:17:06 | FS | Season 10 Lettuce | Std (StVis/IR/NDVI/PRI/CO2/CC) | north-to-south 3ppr standard | 0 | 2 |  |  | 2019-12-26 12:57:51 | 0 days 03:40:45 |
| 2019-12-27 18:26:56 | FS | Season 10 Lettuce | 3D | south-to-north fullfield 3D | 0.05 | 3.5 | 2 | 53 | 2019-12-28 3:22:09 | 0 days 08:55:13 |
| 2019-12-30 10:03:24 | FS | Season 10 Lettuce | Std (StVis/IR/NDVI/PRI/CO2/CC) | fullfield north-to-south std | 0.05 | 2 | 2 | 53 | 2019-12-30 14:51:22 | 0 days 04:47:58 |
| 2019-12-30 19:00:48 | FS | Season 10 Lettuce | 3D | south-to-north fullfield 3D | 0.05 | 3.5 | 2 | 53 | 2019-12-31 3:58:37 | 0 days 08:57:49 |
| 2020-01-02 10:38:58 | FS | Season 10 Lettuce | Std (StVis/IR/NDVI/PRI/CO2/CC) | south-to-north fullfield std | 0.05 | 2 | 2 | 53 | 2020-01-02 15:46:12 | 0 days 05:07:14 |
| 2020-01-02 19:00:08 | FS | Season 10 Lettuce | 3D | south-to-north fullfield 3D | 0.05 | 3.5 | 2 | 53 | 2020-01-03 4:59:33 | 0 days 09:59:25 |
| 2020-01-03 9:32:51 | FS | Season 10 Lettuce | Std (StVis/IR/NDVI/PRI/CO2/CC) | south-to-north fullfield std - paused for drone flight | 0.05 | 2 | 2 | 53 | 2020-01-03 15:46:49 | 0 days 06:13:58 |
| 2020-01-03 12:46:00 | DR | Season 10 Lettuce | DJI Phantom 4 Pro |  |  | 15 | 1 | 54 | 2020-01-03 12:59:00 | 0 days 00:13:00 |
| 2020-01-05 10:38:36 | FS | Season 10 Lettuce | Std (StVis/IR/NDVI/PRI/CO2/CC) | south-to-north fullfield std | 0.05 | 2 | 2 | 53 | 2020-01-05 15:29:39 | 0 days 04:51:03 |
| 2020-01-07 14:38:09 | FS | Season 10 Lettuce | Std (StVis/IR/NDVI/PRI/CO2/CC) | resumption of previous scan | 0.05 | 2 | 2 | 53 | 2020-01-07 15:12:22 | 0 days 00:34:13 |
| 2020-01-07 18:46:46 | FS | Season 10 Lettuce | 3D | south-to-north fullfield 3D | 0.05 | 3.5 | 2 | 53 | 2020-01-08 3:45:11 | 0 days 08:58:25 |
| 2020-01-08 9:56:53 | FS | Season 10 Lettuce | Std (StVis/IR/NDVI/PRI/CO2/CC) | fullfield north-to-south std | 0.05 | 2 | 2 | 53 | 2020-01-08 14:40:12 | 0 days 04:43:19 |
| 2020-01-08 19:01:54 | FS | Season 10 Lettuce | 3D | fullfield 3D south-to-north | 0.05 | 3.5 | 2 | 53 | 2020-01-09 3:55:56 | 0 days 08:54:02 |
| 2020-01-09 13:30:00 | DR | Season 10 Lettuce | DJI Phantom 4 Pro |  |  | 15 | 1 | 54 | 2020-01-09 13:43:00 | 0 days 00:13:00 |
| 2020-01-10 10:02:59 | FS | Season 10 Lettuce | Std (StVis/IR/NDVI/PRI/CO2/CC) | 3ppr standard north-to-south | 0.05 | 2 |  |  | 2020-01-10 13:40:38 | 0 days 03:37:39 |
| 2020-01-11 11:58:00 | DR | Season 10 Lettuce | DJI Phantom 4 Pro |  |  | 15 | 1 | 54 | 2020-01-11 12:11:00 | 0 days 00:13:00 |
| 2020-01-11 20:04:44 | FS | Season 10 Lettuce | 3D | resumption of prior scan/south-to-north fullfield 3d | 0.05 | 3.5 | 2 | 53 | 2020-01-12 5:00:24 | 0 days 08:55:40 |
| 2020-01-13 11:32:08 | FS | Season 10 Lettuce | Std (StVis/IR/NDVI/PRI/CO2/CC) | south-to-north 2ppr standard | 0.05 | 2 |  |  | 2020-01-13 14:00:20 | 0 days 02:28:12 |
| 2020-01-13 19:34:11 | FS | Season 10 Lettuce | 3D | south-to-north fullfield 3d | 0.05 | 3.5 | 2 | 53 | 2020-01-14 4:26:49 | 0 days 08:52:38 |
| 2020-01-14 9:39:51 | FS | Season 10 Lettuce | Std (StVis/IR/NDVI/PRI/CO2/CC) | north-to-south fullfield standard | 0.05 | 2 | 2 | 53 | 2020-01-14 14:25:05 | 0 days 04:45:14 |
| 2020-01-14 15:18:00 | DR | Season 10 Lettuce | DJI Phantom 4 Pro |  |  | 15 | 1 | 54 | 2020-01-14 15:31:00 | 0 days 00:13:00 |
| 2020-01-14 18:59:55 | FS | Season 10 Lettuce | 3D | south-to-north fullfield 3d | 0.05 | 3.5 | 2 | 53 | 2020-01-15 3:52:00 | 0 days 08:52:05 |
| 2020-01-17 12:31:00 | DR | Season 10 Lettuce | DJI Phantom 4 Pro |  |  | 15 | 1 | 54 | 2020-01-17 12:44:00 | 0 days 00:13:00 |
| 2020-01-17 18:50:58 | FS | Season 10 Lettuce | 3D | south-to-north fullfield 3d | 0.1 | 3.5 | 2 | 53 | 2020-01-18 3:44:00 | 0 days 08:53:02 |
| 2020-01-18 9:32:00 | FS | Season 10 Lettuce | Std (StVis/IR/NDVI/PRI/CO2/CC) | north-to-south fullfield standard | 0.1 | 2 | 2 | 53 | 2020-01-18 14:16:40 | 0 days 04:44:40 |
| 2020-01-18 19:27:20 | FS | Season 10 Lettuce | 3D | south-to-north fullfield 3d | 0.1 | 3.5 | 2 | 53 | 2020-01-19 4:50:06 | 0 days 09:22:46 |
| 2020-01-20 9:14:10 | FS | Season 10 Lettuce | Std (StVis/IR/NDVI/PRI/CO2/CC) | north-to-south fullfield standard | 0.1 | 2 | 2 | 53 | 2020-01-20 13:58:11 | 0 days 04:44:01 |
| 2020-01-21 9:38:11 | FS | Season 10 Lettuce | Std (StVis/IR/NDVI/PRI/CO2/CC) | north-to-south fullfield standard | 0.1 | 2 | 2 | 53 | 2020-01-21 14:23:28 | 0 days 04:45:17 |
| 2020-01-22 20:06:05 | FS | Season 10 Lettuce | PS2 | North-to-South PS2 geometric scan on replicate 1 | 0.1 | N/A - at or approaching 0.8m as possible |  |  | 2020-01-23 0:40:40 | 0 days 04:34:35 |
| 2020-01-23 0:43:19 | FS | Season 10 Lettuce | 3D | south-to-north fullfield 3d/ran through sunrise into morning fog/may be worth checking for attenuation due to environment | 0.1 | 3.5 | 2 | 53 | 2020-01-23 9:37:41 | 0 days 08:54:22 |
| 2020-01-23 14:20:00 | DR | Season 10 Lettuce | DJI Phantom 4 Pro |  |  | 15 | 1 | 54 | 2020-01-23 14:33:00 | 0 days 00:13:00 |
| 2020-01-23 19:57:23 | FS | Season 10 Lettuce | PS2 | North-to-South PS2 geometric scan on replicate 2/<notes> | 0.1 | N/A - at or approaching 0.8m as possible |  |  | 2020-01-24 0:33:13 | 0 days 04:35:50 |
| 2020-01-24 19:57:44 | FS | Season 10 Lettuce | PS2 | North-to-South PS2 geometric scan on replicate 3/<notes> | 0.1 | N/A - at or approaching 0.8m as possible |  |  | 2020-01-25 0:31:16 | 0 days 04:33:32 |
| 2020-01-25 9:42:05 | FS | Season 10 Lettuce | Std (StVis/IR/NDVI/PRI/CO2/CC) | fullfield north-to-south standard | 0.1 | 2 | 2 | 53 | 2020-01-25 14:25:07 | 0 days 04:43:02 |
| 2020-01-25 19:05:02 | FS | Season 10 Lettuce | 3D | fullfield south-to-north 3d | 0.1 | 3.5 | 2 | 53 | 2020-01-26 5:32:43 | 0 days 10:27:41 |
| 2020-01-26 16:30:48 | FS | Season 10 Lettuce | Std (StVis/IR/NDVI/PRI/CO2/CC) | Scheduler Test | 0.1 | 2 |  |  | 2020-01-26 16:31:35 | 0 days 00:00:47 |
| 2020-01-26 17:30:44 | FS | Season 10 Lettuce | Std (StVis/IR/NDVI/PRI/CO2/CC) | Scheduler Test | 0.1 | 2 |  |  | 2020-01-26 17:31:21 | 0 days 00:00:37 |
| 2020-01-26 18:09:10 | FS | Season 10 Lettuce | 3D | test scan | 0.1 | 3.5 |  |  | 2020-01-26 18:10:50 | 0 days 00:01:40 |
| 2020-01-26 21:18:43 | FS | Season 10 Lettuce | 3D | test scan | 0.1 | 3.5 |  |  | 2020-01-26 21:19:41 | 0 days 00:00:58 |
| 2020-01-27 19:59:59 | FS | Season 10 Lettuce | 3D | fullfield south-to-north 3d | 0.1 | 3.5 | 2 | 53 | 2020-01-28 5:05:45 | 0 days 09:05:46 |
| 2020-01-28 8:41:42 | FS | Season 10 Lettuce | 3D | Metadata test | 0.1 | 3.5 |  |  | 2020-01-28 8:42:03 | 0 days 00:00:21 |
| 2020-01-28 9:17:08 | FS | Season 10 Lettuce | 3D | Metadata test | 0.1 | 3.5 |  |  | 2020-01-28 9:18:49 | 0 days 00:01:41 |
| 2020-01-28 9:52:52 | FS | Season 10 Lettuce | Std (StVis/IR/NDVI/PRI/CO2/CC) | fullfield north-to-south standard | 0.1 | 2 | 2 | 53 | 2020-01-28 14:36:06 | 0 days 04:43:14 |
| 2020-01-28 19:20:08 | FS | Season 10 Lettuce | 3D | fullfield south-to-north 3d | 0.1 | 3.5 | 2 | 53 | 2020-01-29 4:29:43 | 0 days 09:09:35 |
| 2020-01-29 9:25:09 | FS | Season 10 Lettuce | Std (StVis/IR/NDVI/PRI/CO2/CC) | fullfield north-to-south standard | 0.15 | 2 | 2 | 53 | 2020-01-29 14:08:05 | 0 days 04:42:56 |
| 2020-01-29 15:00:44 | FS | Season 10 Lettuce | StVis | series of 50 images collected of a checkerboard (2.25 inch squares) at varying positions at 2m distance between camera and table |  | 2 |  |  | 2020-01-29 15:22:46 | 0 days 00:22:02 |
| 2020-01-29 15:07:00 | DR | Season 10 Lettuce | DJI Phantom 4 Pro |  |  | 15 | 1 | 54 | 2020-01-29 15:20:00 | 0 days 00:13:00 |
| 2020-01-29 15:35:51 | FS | Season 10 Lettuce | StVis | series of 25 images collected of a checkerboard (2.25 inch squares) at varying positions at 1m distance between camera and table |  | 1 |  |  | 2020-01-29 15:46:56 | 0 days 00:11:05 |
| 2020-01-29 15:48:49 | FS | Season 10 Lettuce | StVis | series of 25 images collected of a checkerboard (2.25 inch squares) at varying positions at 3m distance between camera and table |  | 3 |  |  | 2020-01-29 16:00:11 | 0 days 00:11:22 |
| 2020-01-29 19:11:09 | FS | Season 10 Lettuce | 3D | south-to-north fullfield 3d | 0.15 | 3.5 | 2 | 53 | 2020-01-30 4:05:55 | 0 days 08:54:46 |
| 2020-01-30 9:30:44 | FS | Season 10 Lettuce | Std (StVis/IR/NDVI/PRI/CO2/CC) | south-to-north 2ppr standard | 0.15 | 2 |  |  | 2020-01-30 11:58:42 | 0 days 02:27:58 |
| 2020-01-30 12:10:44 | FS | Season 10 Lettuce | Std (StVis/IR/NDVI/PRI/CO2/CC) | north-to-south 1ppr standard | 0.15 | 2 |  |  | 2020-01-30 13:29:00 | 0 days 01:18:16 |
| 2020-01-30 19:04:04 | FS | Season 10 Lettuce | 3D | south-to-north fullfield 3d | 0.15 | 3.5 | 2 | 53 | 2020-01-31 3:59:36 | 0 days 08:55:32 |
| 2020-01-31 9:27:31 | FS | Season 10 Lettuce | Std (StVis/IR/NDVI/PRI/CO2/CC) | north-to-south fullfield standard | 0.15 | 2 | 2 | 53 | 2020-01-31 14:10:44 | 0 days 04:43:13 |
| 2020-01-31 19:15:39 | FS | Season 10 Lettuce | 3D | south-to-north fullfield 3d | 0.15 | 3.5 | 2 | 53 | 2020-02-01 4:10:02 | 0 days 08:54:23 |
| 2020-02-03 9:32:43 | FS | Season 10 Lettuce | Std (StVis/IR/NDVI/PRI/CO2/CC) | north-to-south fullfield standard/windy | 0.15 | 2 | 2 | 53 | 2020-02-03 14:16:42 | 0 days 04:43:59 |
| 2020-02-04 9:30:45 | FS | Season 10 Lettuce | Std (StVis/IR/NDVI/PRI/CO2/CC) | south-to-north 2ppr standard | 0.15 | 2 |  |  | 2020-02-04 11:58:58 | 0 days 02:28:13 |
| 2020-02-04 12:10:46 | FS | Season 10 Lettuce | Std (StVis/IR/NDVI/PRI/CO2/CC) | north-to-south 1ppr standard | 0.15 | 2 |  |  | 2020-02-04 13:28:23 | 0 days 01:17:37 |
| 2020-02-04 15:15:00 | DR | Season 10 Lettuce | DJI Phantom 4 Pro |  |  | 15 | 1 | 54 | 2020-02-04 15:28:00 | 0 days 00:13:00 |
| 2020-02-04 19:39:22 | FS | Season 10 Lettuce | 3D | south-to-north fullfield 3d | 0.15 | 3.5 | 2 | 53 | 2020-02-05 4:33:45 | 0 days 08:54:23 |
| 2020-02-05 19:55:54 | FS | Season 10 Lettuce | PS2 | North-to-South PS2 geometric scan on replicate 1/<notes> | 0.15 | N/A - at or approaching 0.8m as possible |  |  | 2020-02-06 0:30:10 | 0 days 04:34:16 |
| 2020-02-06 14:58:00 | DR | Season 10 Lettuce | DJI Phantom 4 Pro |  |  | 15 | 1 | 54 | 2020-02-06 15:11:00 | 0 days 00:13:00 |
| 2020-02-06 20:02:45 | FS | Season 10 Lettuce | PS2 | North-to-South PS2 geometric scan on replicate 2/<notes> | 0.15 | N/A - at or approaching 0.8m as possible |  |  | 2020-02-07 0:38:28 | 0 days 04:35:43 |
| 2020-02-07 20:07:14 | FS | Season 10 Lettuce | PS2 | North-to-South PS2 geometric scan on replicate 3/<notes> | 0.15 | N/A - at or approaching 0.8m as possible |  |  | 2020-02-08 0:41:00 | 0 days 04:33:46 |
| 2020-02-08 9:30:44 | FS | Season 10 Lettuce | Std (StVis/IR/NDVI/PRI/CO2/CC) | north-to-south fullfield standard | 0.15 | 2 | 2 | 53 | 2020-02-08 14:13:09 | 0 days 04:42:25 |
| 2020-02-08 19:00:46 | FS | Season 10 Lettuce | 3D | south-to-north fullfield 3d | 0.15 | 3.5 | 2 | 53 | 2020-02-09 4:59:13 | 0 days 09:58:27 |
| 2020-02-11 10:00:04 | FS | Season 10 Lettuce | Std (StVis/IR/NDVI/PRI/CO2/CC) | north-to-south 2ppr standard | 0.15 | 2 |  |  | 2020-02-11 12:27:04 | 0 days 02:27:00 |
| 2020-02-11 12:43:00 | DR | Season 10 Lettuce | DJI Phantom 4 Pro |  |  | 15 | 1 | 54 | 2020-02-11 12:56:00 | 0 days 00:13:00 |
| 2020-02-11 19:36:42 | FS | Season 10 Lettuce | 3D | south-to-north fullfield 3d | 0.15 | 3.5 | 2 | 53 | 2020-02-12 6:00:47 | 0 days 10:24:05 |
| 2020-02-12 14:19:06 | FS | Season 10 Lettuce | Std (StVis/IR/NDVI/PRI/CO2/CC) | north-to-south 1ppr standard | 0.15 | 2 |  |  | 2020-02-12 15:41:13 | 0 days 01:22:07 |
| 2020-02-12 19:56:52 | FS | Season 10 Lettuce | PS2 | North-to-South PS2 geometric scan on replicate 1/<notes> | 0.15 | N/A - at or approaching 0.8m as possible |  |  | 2020-02-13 0:33:05 | 0 days 04:36:13 |
| 2020-02-13 19:59:11 | FS | Season 10 Lettuce | PS2 | North-to-South PS2 geometric scan on replicate 2/<notes> | 0.15 | N/A - at or approaching 0.8m as possible |  |  | 2020-02-14 0:33:49 | 0 days 04:34:38 |
| 2020-02-14 19:52:50 | FS | Season 10 Lettuce | PS2 | North-to-South PS2 geometric scan on replicate 3/<notes> | 0.15 | N/A - at or approaching 0.8m as possible |  |  | 2020-02-15 0:27:54 | 0 days 04:35:04 |
| 2020-02-15 9:25:18 | FS | Season 10 Lettuce | Std (StVis/IR/NDVI/PRI/CO2/CC) | north-to-south fullfield standard | 0.15 | 2 | 2 | 53 | 2020-02-15 14:11:04 | 0 days 04:45:46 |
| 2020-02-15 19:56:57 | FS | Season 10 Lettuce | 3D | south-to-north fullfield 3d | 0.15 | 3.5 | 2 | 53 | 2020-02-16 4:52:03 | 0 days 08:55:06 |
| 2020-02-16 11:00:44 | FS | Season 10 Lettuce | Std (StVis/IR/NDVI/PRI/CO2/CC) | north-to-south 1ppr standard | 0.15 | 2 |  |  | 2020-02-16 12:19:07 | 0 days 01:18:23 |
| 2020-02-16 12:30:44 | FS | Season 10 Lettuce | IR | Part 1 of a series of test thermal scans for thermal interpolation experiment/<notes> | 0.15 | 2 |  |  | 2020-02-16 12:42:11 | 0 days 00:11:27 |
| 2020-02-16 12:50:43 | FS | Season 10 Lettuce | IR | Part 3 of a series of test thermal scans for thermal interpolation experiment/<notes> | 0.15 | 2 |  |  | 2020-02-16 12:59:22 | 0 days 00:08:39 |
| 2020-02-16 13:00:44 | FS | Season 10 Lettuce | IR | Part 4 of a series of test thermal scans for thermal interpolation experiment/<notes> | 0.15 | 2 |  |  | 2020-02-16 13:10:10 | 0 days 00:09:26 |
| 2020-02-16 20:00:44 | FS | Season 10 Lettuce | 3D | south-to-north fullfield 3d | 0.15 | 3.5 | 2 | 53 | 2020-02-17 4:57:14 | 0 days 08:56:30 |
| 2020-02-17 9:15:13 | FS | Season 10 Lettuce | Std (StVis/IR/NDVI/PRI/CO2/CC) | north-to-south 3ppr standard | 0.15 | 2 |  |  | 2020-02-17 12:58:44 | 0 days 03:43:31 |
| 2020-02-17 13:05:00 | DR | Season 10 Lettuce | DJI Phantom 4 Pro |  |  | 15 | 1 | 54 | 2020-02-17 13:16:00 | 0 days 00:11:00 |
| 2020-02-17 19:59:57 | FS | Season 10 Lettuce | 3D | south-to-north fullfield 3d | 0.15 | 3.5 | 2 | 53 | 2020-02-18 4:54:30 | 0 days 08:54:33 |
| 2020-02-18 9:34:34 | FS | Season 10 Lettuce | Std (StVis/IR/NDVI/PRI/CO2/CC) | north-to-south fullfield standard | 0.15 | 2 | 2 | 53 | 2020-02-18 14:21:07 | 0 days 04:46:33 |
| 2020-02-18 19:30:09 | FS | Season 10 Lettuce | 3D | south-to-north fullfield 3d | 0.15 | 3.5 | 2 | 53 | 2020-02-19 4:25:26 | 0 days 08:55:17 |
| 2020-02-19 20:09:34 | FS | Season 10 Lettuce | PS2 | North-to-South PS2 geometric scan on replicate 1/<notes> | 0.15 | N/A - at or approaching 0.8m as possible |  |  | 2020-02-20 0:46:10 | 0 days 04:36:36 |
| 2020-02-20 14:32:00 | DR | Season 10 Lettuce | DJI Phantom 4 Pro |  |  | 15 | 1 | 54 | 2020-02-20 14:44:00 | 0 days 00:12:00 |
| 2020-02-20 19:56:26 | FS | Season 10 Lettuce | PS2 | North-to-South PS2 geometric scan on replicate 3/<notes> | 0.15 | N/A - at or approaching 0.8m as possible |  |  | 2020-02-21 0:31:03 | 0 days 04:34:37 |
| 2020-02-24 9:14:36 | FS | Season 10 Lettuce | Std (StVis/IR/NDVI/PRI/CO2/CC) | spacing reduced for imaging systems from 0.5m to 0.25m to increase overlap/north-to-south fullfield standard | 0.15 | 2 | 2 | 53 | 2020-02-24 14:03:26 | 0 days 04:48:50 |
| 2020-02-24 14:17:00 | DR | Season 10 Lettuce | DJI Phantom 4 Pro |  |  | 15 | 1 | 54 | 2020-02-24 14:28:00 | 0 days 00:11:00 |
| 2020-02-24 19:37:36 | FS | Season 10 Lettuce | 3D | south-to-north fullfield 3d | 0.15 | 3.5 | 2 | 53 | 2020-02-25 4:32:06 | 0 days 08:54:30 |
| 2020-02-26 14:20:17 | FS | Season 10 Lettuce | Std (StVis/IR/NDVI/PRI/CO2/CC) | north-to-south 1ppr standard | 0.15 | 2 |  |  | 2020-02-26 15:39:27 | 0 days 01:19:10 |
| 2020-02-26 20:02:33 | FS | Season 10 Lettuce | PS2 | North-to-South PS2 geometric scan on replicate 1/<notes> | 0.15 | N/A - at or approaching 0.8m as possible |  |  | 2020-02-27 0:39:30 | 0 days 04:36:57 |
| 2020-02-27 14:37:00 | DR | Season 10 Lettuce | DJI Phantom 4 Pro |  |  | 15 | 1 | 54 | 2020-02-27 14:48:00 | 0 days 00:11:00 |
| 2020-02-27 19:58:35 | FS | Season 10 Lettuce | PS2 | North-to-South PS2 geometric scan on replicate 3/<notes> | 0.15 | N/A - at or approaching 0.8m as possible |  |  | 2020-02-28 0:37:23 | 0 days 04:38:48 |
| 2020-02-28 19:43:29 | FS | Season 10 Lettuce | 3D | south-to-north fullfield 3d | 0.15 | 3.5 | 2 | 53 | 2020-02-29 4:37:46 | 0 days 08:54:17 |
| 2020-02-29 19:46:46 | FS | Season 10 Lettuce | 3D | north-to-south fullfield 3d | 0.15 | 3.5 | 2 | 53 | 2020-03-01 4:44:19 | 0 days 08:57:33 |
| 2020-03-01 9:37:27 | FS | Season 10 Lettuce | Std (StVis/IR/NDVI/PRI/CO2/CC) | no FLIR data - sensor server issue/north-to-south fullfield standard | 0.15 | 2 | 2 | 53 | 2020-03-01 14:21:27 | 0 days 04:44:00 |
| 2020-03-01 18:59:47 | FS | Season 10 Lettuce | 3D | south-to-north fullfield 3d | 0.15 | 3.5 | 2 | 53 | 2020-03-02 3:54:15 | 0 days 08:54:28 |
| 2020-03-02 8:57:36 | FS | Season 10 Lettuce | Std (StVis/IR/NDVI/PRI/CO2/CC) | north-to-south 2ppr standard | 0.15 | 2 |  |  | 2020-03-02 11:30:34 | 0 days 02:32:58 |
| 2020-03-02 13:43:28 | FS | Season 10 Lettuce | Std (StVis/IR/NDVI/PRI/CO2/CC) | north-to-south 1ppr standard/spraypainting of multiple plants and harvest preparations in-progress | 0.15 | 2 |  |  | 2020-03-02 15:05:51 | 0 days 01:22:23 |
| 2020-03-03 8:23:00 | DR | Season 10 Lettuce | DJI Phantom 4 Pro |  |  | 15 | 1 | 54 | 2020-03-03 8:34:00 | 0 days 00:11:00 |
| 2020-03-03 8:45:10 | FS | Season 10 Lettuce | Std (StVis/IR/NDVI/PRI/CO2/CC) | north-to-south fullfield standard/multiple plants spraypainted/harvest in-progress | 0.15 | 2 | 2 | 53 | 2020-03-03 13:27:54 | 0 days 04:42:44 |
| 2020-06-19 14:09:00 | DR | Season 11 Sorghum | DJI Phantom 4 Pro |  |  | 15 | 1 | 54 | 2020-06-19 14:20:00 | 0 days 00:11:00 |
| 2020-06-23 10:36:47 | FS | Season 11 Sorghum | StVis | fullfield rgb | 0 | 3.5 | 2 | 54 | 2020-06-23 14:32:57 | 0 days 03:56:10 |
| 2020-06-23 20:00:29 | FS | Season 11 Sorghum | 3D | fullfield laser | 0 | 3.5 | 2 | 54 | 2020-06-24 5:06:11 | 0 days 09:05:42 |
| 2020-06-24 10:32:13 | FS | Season 11 Sorghum | StVis | fullfield rgb | 0 | 3.5 | 2 | 54 | 2020-06-24 14:35:41 | 0 days 04:03:28 |
| 2020-06-25 10:30:10 | FS | Season 11 Sorghum | StVis | fullfield rgb | 0 | 3.5 | 2 | 54 | 2020-06-25 14:34:15 | 0 days 04:04:05 |
| 2020-06-25 19:55:48 | FS | Season 11 Sorghum | 3D | fullfield laser | 0 | 3.5 | 2 | 54 | 2020-06-26 5:08:10 | 0 days 09:12:22 |
| 2020-06-26 10:39:35 | FS | Season 11 Sorghum | StVis | fullfield rgb | 0 | 3.5 | 2 | 54 | 2020-06-26 14:38:07 | 0 days 03:58:32 |
| 2020-06-26 19:54:55 | FS | Season 11 Sorghum | 3D | fullfield laser | 0 | 3.5 | 2 | 54 | 2020-06-27 5:01:37 | 0 days 09:06:42 |
| 2020-06-30 10:30:46 | FS | Season 11 Sorghum | StVis | fullfield rgb | 0 | 3.5 | 2 | 54 | 2020-06-30 14:29:02 | 0 days 03:58:16 |
| 2020-06-30 20:28:13 | FS | Season 11 Sorghum | 3D | fullfield laser | 0 | 3.5 | 2 | 54 | 2020-07-01 5:34:09 | 0 days 09:05:56 |
| 2020-07-02 10:06:00 | DR | Season 11 Sorghum | DJI Phantom 4 Pro |  |  | 15 | 1 | 54 | 2020-07-02 10:17:00 | 0 days 00:11:00 |
| 2020-07-02 10:32:25 | FS | Season 11 Sorghum | StVis | fullfield rgb | 0 | 3.5 | 2 | 54 | 2020-07-02 14:29:33 | 0 days 03:57:08 |
| 2020-07-03 20:01:34 | FS | Season 11 Sorghum | 3D | fullfield laser | 0.1 | 3.5 | 2 | 54 | 2020-07-04 5:09:24 | 0 days 09:07:50 |
| 2020-07-04 10:30:16 | FS | Season 11 Sorghum | StVis | fullfield rgb | 0.1 | 3.5 | 2 | 54 | 2020-07-04 14:27:44 | 0 days 03:57:28 |
| 2020-07-06 10:26:01 | FS | Season 11 Sorghum | StVis | fullfield rgb | 0.1 | 3.5 | 2 | 54 | 2020-07-06 14:27:47 | 0 days 04:01:46 |
| 2020-07-06 20:27:26 | FS | Season 11 Sorghum | 3D | fullfield laser | 0.1 | 3.5 | 2 | 54 | 2020-07-07 5:32:45 | 0 days 09:05:19 |
| 2020-07-07 10:47:10 | FS | Season 11 Sorghum | StVis | fullfield rgb | 0.1 | 3.5 | 2 | 54 | 2020-07-07 14:43:38 | 0 days 03:56:28 |
| 2020-07-07 20:19:45 | FS | Season 11 Sorghum | 3D | fullfield laser | 0.1 | 3.5 | 2 | 54 | 2020-07-08 5:27:27 | 0 days 09:07:42 |
| 2020-07-08 13:44:00 | DR | Season 11 Sorghum | DJI Phantom 4 Pro |  |  | 15 | 1 | 54 | 2020-07-08 13:55:00 | 0 days 00:11:00 |
| 2020-07-08 20:09:52 | FS | Season 11 Sorghum | 3D | fullfield laser | 0.1 | 3.5 | 2 | 54 | 2020-07-09 5:16:02 | 0 days 09:06:10 |
| 2020-07-10 10:32:31 | FS | Season 11 Sorghum | StVis | fullfield rgb | 0.15 | 3.5 | 2 | 54 | 2020-07-10 14:31:12 | 0 days 03:58:41 |
| 2020-07-10 20:34:40 | FS | Season 11 Sorghum | 3D | fullfield laser - no data due to initialization error | 0.15 | 3.5 | 2 | 54 | 2020-07-11 5:41:37 | 0 days 09:06:57 |
| 2020-07-11 10:33:02 | FS | Season 11 Sorghum | StVis | fullfield rgb | 0.15 | 3.5 | 2 | 54 | 2020-07-11 14:31:22 | 0 days 03:58:20 |
| 2020-07-11 21:40:59 | FS | Season 11 Sorghum | 3D | fullfield laser - no data due to initialization error | 0.15 | 3.5 | 2 | 54 | 2020-07-12 6:48:44 | 0 days 09:07:45 |
| 2020-07-12 10:24:26 | FS | Season 11 Sorghum | StVis | fullfield rgb | 0.15 | 3.5 | 2 | 54 | 2020-07-12 14:22:04 | 0 days 03:57:38 |
| 2020-07-13 10:37:11 | FS | Season 11 Sorghum | StVis | fullfield rgb/cropcircle bad | 0.2 | 3.5 | 2 | 54 | 2020-07-13 14:35:55 | 0 days 03:58:44 |
| 2020-07-13 20:32:14 | FS | Season 11 Sorghum | 3D | fullfield laser | 0.2 | 3.5 | 2 | 54 | 2020-07-14 5:40:22 | 0 days 09:08:08 |
| 2020-07-14 20:30:25 | FS | Season 11 Sorghum | 3D | fullfield laser | 0.2 | 3.5 | 2 | 54 | 2020-07-15 5:36:30 | 0 days 09:06:05 |
| 2020-07-15 13:29:04 | FS | Season 11 Sorghum | StVis | resumption of previous scan | 0.2 | 3.5 | 2 | 54 | 2020-07-15 15:12:45 | 0 days 01:43:41 |
| 2020-07-15 20:32:27 | FS | Season 11 Sorghum | 3D | fullfield laser | 0.2 | 3.5 | 2 | 54 | 2020-07-16 5:39:07 | 0 days 09:06:40 |
| 2020-07-16 10:29:41 | FS | Season 11 Sorghum | StVis | fullfield RGB | 0.2 | 3.5 | 2 | 54 | 2020-07-16 14:25:49 | 0 days 03:56:08 |
| 2020-07-16 14:56:00 | DR | Season 11 Sorghum | DJI Phantom 4 Pro |  |  | 15 | 1 | 54 | 2020-07-16 15:07:00 | 0 days 00:11:00 |
| 2020-07-16 20:30:46 | FS | Season 11 Sorghum | 3D | fullfield laser | 0.35 | 3.5 | 2 | 54 | 2020-07-17 5:36:54 | 0 days 09:06:08 |
| 2020-07-17 10:46:08 | FS | Season 11 Sorghum | StVis | fullfield RGB | 0.35 | 3.5 | 2 | 54 | 2020-07-17 14:43:46 | 0 days 03:57:38 |
| 2020-07-17 20:32:21 | FS | Season 11 Sorghum | 3D | fullfield laser | 0.35 | 3.5 | 2 | 54 | 2020-07-18 5:39:53 | 0 days 09:07:32 |
| 2020-07-20 10:28:19 | FS | Season 11 Sorghum | StVis | fullfield RGB | 0.35 | 3.5 | 2 | 54 | 2020-07-20 14:24:57 | 0 days 03:56:38 |
| 2020-07-20 20:41:17 | FS | Season 11 Sorghum | 3D | fullfield laser | 0.35 | 3.5 | 2 | 54 | 2020-07-21 5:48:54 | 0 days 09:07:37 |
| 2020-07-21 20:13:28 | FS | Season 11 Sorghum | 3D | fullfield laser | 0.45 | 3.5 | 2 | 54 | 2020-07-22 5:18:38 | 0 days 09:05:10 |
| 2020-07-22 10:33:18 | FS | Season 11 Sorghum | StVis | fullfield RGB | 0.45 | 3.5 | 2 | 54 | 2020-07-22 14:36:32 | 0 days 04:03:14 |
| 2020-07-24 10:30:25 | FS | Season 11 Sorghum | StVis | fullfield RGB | 0.5 | 3.5 | 2 | 54 | 2020-07-24 14:31:39 | 0 days 04:01:14 |
| 2020-07-24 16:21:00 | DR | Season 11 Sorghum | DJI Phantom 4 Pro |  |  | 15 | 1 | 54 | 2020-07-24 16:33:00 | 0 days 00:12:00 |
| 2020-07-25 10:21:25 | FS | Season 11 Sorghum | StVis | fullfield RGB | 0.5 | 3.5 | 2 | 54 | 2020-07-25 14:18:37 | 0 days 03:57:12 |
| 2020-07-25 20:26:23 | FS | Season 11 Sorghum | 3D | fullfield laser | 0.5 | 3.5 | 2 | 54 | 2020-07-26 5:33:22 | 0 days 09:06:59 |
| 2020-07-26 10:27:46 | FS | Season 11 Sorghum | StVis | fullfield RGB | 0.5 | 3.5 | 2 | 54 | 2020-07-26 14:24:56 | 0 days 03:57:10 |
| 2020-07-26 20:24:43 | FS | Season 11 Sorghum | 3D | fullfield laser | 0.5 | 3.5 | 2 | 54 | 2020-07-27 5:31:54 | 0 days 09:07:11 |
| 2020-07-27 12:55:30 | FS | Season 11 Sorghum | StVis | manual stop - rgb sensor server restarted and scan resumed from last reentry point | 0.5 | 3.5 | 2 | 54 | 2020-07-27 14:27:47 | 0 days 01:32:17 |
| 2020-07-27 20:18:38 | FS | Season 11 Sorghum | 3D | fullfield laser | 0.5 | 3.5 | 2 | 54 | 2020-07-28 5:25:22 | 0 days 09:06:44 |
| 2020-07-28 13:53:12 | FS | Season 11 Sorghum | IR | fullfield thermal 1ppr | 0.6 | 2 |  |  | 2020-07-28 15:11:58 | 0 days 01:18:46 |
| 2020-07-28 21:14:14 | FS | Season 11 Sorghum | IR | range 28 thermal 1ppr | 0.6 | 2 |  |  | 2020-07-28 21:16:19 | 0 days 00:02:05 |
| 2020-07-28 21:19:26 | FS | Season 11 Sorghum | 3D | fullfield laser | 0.6 | 3.5 | 2 | 54 | 2020-07-29 6:23:56 | 0 days 09:04:30 |
| 2020-07-29 10:29:11 | FS | Season 11 Sorghum | StVis | fullfield RGB | 0.6 | 3.5 | 2 | 54 | 2020-07-29 14:25:06 | 0 days 03:55:55 |
| 2020-07-29 20:29:36 | FS | Season 11 Sorghum | 3D | fullfield laser | 0.6 | 3.5 | 2 | 54 | 2020-07-30 5:32:24 | 0 days 09:02:48 |
| 2020-07-30 20:35:19 | FS | Season 11 Sorghum | 3D | fullfield laser | 0.6 | 3.5 | 2 | 54 | 2020-07-31 5:38:31 | 0 days 09:03:12 |
| 2020-07-31 12:44:34 | FS | Season 11 Sorghum | IR | fullfield thermal 1ppr | 0.6 | 3.5 |  |  | 2020-07-31 14:05:34 | 0 days 01:21:00 |
| 2020-07-31 22:42:23 | FS | Season 11 Sorghum | IR | fullfield thermal 1ppr | 0.6 | 3.5 |  |  | 2020-08-01 0:03:14 | 0 days 01:20:51 |
| 2020-08-01 10:23:46 | FS | Season 11 Sorghum | StVis | fullfield RGB | 0.6 | 3.5 | 2 | 54 | 2020-08-01 14:19:16 | 0 days 03:55:30 |
| 2020-08-01 20:19:14 | FS | Season 11 Sorghum | 3D | fullfield laser | 0.6 | 3.5 | 2 | 54 | 2020-08-02 5:21:58 | 0 days 09:02:44 |
| 2020-08-03 10:20:27 | FS | Season 11 Sorghum | StVis | fullfield RGB | 0.65 | 3.5 | 2 | 54 | 2020-08-03 14:15:40 | 0 days 03:55:13 |
| 2020-08-03 20:36:58 | FS | Season 11 Sorghum | 3D | fullfield laser | 0.65 | 3.5 | 2 | 54 | 2020-08-04 5:39:48 | 0 days 09:02:50 |
| 2020-08-04 11:47:59 | FS | Season 11 Sorghum | IR | fullfield thermal 1ppr | 0.65 | 3.5 |  |  | 2020-08-04 13:09:07 | 0 days 01:21:08 |
| 2020-08-04 22:57:43 | FS | Season 11 Sorghum | IR | fullfield thermal 1ppr | 0.65 | 3.5 |  |  | 2020-08-05 0:20:32 | 0 days 01:22:49 |
| 2020-08-05 11:29:20 | FS | Season 11 Sorghum | StVis | fullfield RGB | 0.65 | 3.5 | 2 | 54 | 2020-08-05 15:32:53 | 0 days 04:03:33 |
| 2020-08-05 20:27:02 | FS | Season 11 Sorghum | 3D | fullfield laser | 0.65 | 3.5 | 2 | 54 | 2020-08-06 5:29:28 | 0 days 09:02:26 |
| 2020-08-06 10:34:50 | FS | Season 11 Sorghum | StVis | fullfield RGB | 0.7 | 3.5 | 2 | 54 | 2020-08-06 14:36:44 | 0 days 04:01:54 |
| 2020-08-06 14:50:00 | DR | Season 11 Sorghum | DJI Phantom 4 Pro |  |  | 15 | 1 | 54 | 2020-08-06 15:01:00 | 0 days 00:11:00 |
| 2020-08-06 20:46:24 | FS | Season 11 Sorghum | 3D | fullfield laser | 0.7 | 3.5 | 2 | 54 | 2020-08-07 5:49:38 | 0 days 09:03:14 |
| 2020-08-07 12:39:19 | FS | Season 11 Sorghum | IR | fullfield thermal 1ppr | 0.7 | 3.5 |  |  | 2020-08-07 13:59:51 | 0 days 01:20:32 |
| 2020-08-07 22:37:11 | FS | Season 11 Sorghum | IR | fullfield thermal 1ppr | 0.7 | 3.5 |  |  | 2020-08-07 23:57:53 | 0 days 01:20:42 |
| 2020-08-08 10:46:11 | FS | Season 11 Sorghum | StVis | fullfield RGB | 0.7 | 3.5 | 2 | 54 | 2020-08-08 14:47:04 | 0 days 04:00:53 |
| 2020-08-08 20:30:16 | FS | Season 11 Sorghum | 3D | fullfield laser | 0.7 | 3.5 | 2 | 54 | 2020-08-09 5:33:46 | 0 days 09:03:30 |
| 2020-08-10 10:29:15 | FS | Season 11 Sorghum | StVis | fullfield RGB | 0.7 | 3.5 | 2 | 54 | 2020-08-10 14:31:31 | 0 days 04:02:16 |
| 2020-08-10 20:07:45 | FS | Season 11 Sorghum | 3D | fullfield laser | 0.7 | 3.5 | 2 | 54 | 2020-08-11 5:09:10 | 0 days 09:01:25 |
| 2020-08-11 13:09:39 | FS | Season 11 Sorghum | IR | fullfield thermal 1ppr | 1 | 3.5 |  |  | 2020-08-11 14:30:50 | 0 days 01:21:11 |
| 2020-08-11 22:53:38 | FS | Season 11 Sorghum | IR | fullfield thermal 1ppr | 1 | 3.5 |  |  | 2020-08-12 0:16:25 | 0 days 01:22:47 |
| 2020-08-12 10:40:23 | FS | Season 11 Sorghum | StVis | fullfield RGB | 1 | 3.5 | 2 | 54 | 2020-08-12 14:40:20 | 0 days 03:59:57 |
| 2020-08-12 20:13:25 | FS | Season 11 Sorghum | 3D | fullfield laser | 1 | 3.5 | 2 | 54 | 2020-08-13 5:16:35 | 0 days 09:03:10 |
| 2020-08-13 10:21:25 | FS | Season 11 Sorghum | StVis | fullfield RGB | 1 | 3.5 | 2 | 54 | 2020-08-13 14:21:09 | 0 days 03:59:44 |
| 2020-08-13 14:38:00 | DR | Season 11 Sorghum | DJI Phantom 4 Pro |  |  | 15 | 1 | 54 | 2020-08-13 14:49:00 | 0 days 00:11:00 |
| 2020-08-13 20:30:07 | FS | Season 11 Sorghum | 3D | fullfield laser | 1 | 3.5 | 2 | 54 | 2020-08-14 5:32:50 | 0 days 09:02:43 |
| 2020-08-14 12:28:11 | FS | Season 11 Sorghum | IR | fullfield thermal 1ppr | 1 | 3.5 |  |  | 2020-08-14 13:48:56 | 0 days 01:20:45 |
| 2020-08-14 22:38:11 | FS | Season 11 Sorghum | IR | fullfield thermal 1ppr | 1 | 3.5 |  |  | 2020-08-14 23:56:26 | 0 days 01:18:15 |
| 2020-08-15 10:31:07 | FS | Season 11 Sorghum | StVis | fullfield RGB | 1 | 3.5 | 2 | 54 | 2020-08-15 14:31:16 | 0 days 04:00:09 |
| 2020-08-15 20:40:33 | FS | Season 11 Sorghum | 3D | fullfield laser | 1 | 3.5 | 2 | 54 | 2020-08-16 5:43:10 | 0 days 09:02:37 |
| 2020-08-18 12:46:38 | FS | Season 11 Sorghum | IR | fullfield thermal 1ppr | 1 | 3.5 |  |  | 2020-08-18 14:13:26 | 0 days 01:26:48 |
| 2020-08-18 23:30:09 | FS | Season 11 Sorghum | IR | fullfield thermal 1ppr | 1 | 3.5 |  |  | 2020-08-19 0:55:21 | 0 days 01:25:12 |
| 2020-08-19 11:36:04 | FS | Season 11 Sorghum | StVis | fullfield RGB | 1 | 3.5 | 2 | 54 | 2020-08-19 15:31:16 | 0 days 03:55:12 |
| 2020-08-20 14:44:16 | FS | Season 11 Sorghum | StVis | resumption of previous scan | 1 | 3.5 | 2 | 54 | 2020-08-20 14:49:11 | 0 days 00:04:55 |
| 2020-08-21 10:20:00 | DR | Season 11 Sorghum | DJI Phantom 4 Pro |  |  | 15 | 1 | 54 | 2020-08-21 10:31:00 | 0 days 00:11:00 |
| 2020-08-21 13:23:13 | FS | Season 11 Sorghum | IR | fullfield thermal 1ppr | 1 | 3.5 |  |  | 2020-08-21 14:43:26 | 0 days 01:20:13 |
| 2020-08-21 22:38:46 | FS | Season 11 Sorghum | IR | fullfield thermal 1ppr | 1 | 3.5 |  |  | 2020-08-21 23:59:33 | 0 days 01:20:47 |
| 2020-08-22 0:07:16 | FS | Season 11 Sorghum | 3D | fullfield laser | 1 | 3.5 | 2 | 54 | 2020-08-22 9:08:57 | 0 days 09:01:41 |
| 2020-08-22 13:27:09 | FS | Season 11 Sorghum | StVis | resumption of previous scan | 1 | 3.5 | 2 | 54 | 2020-08-22 14:42:28 | 0 days 01:15:19 |
| 2020-08-22 19:59:15 | FS | Season 11 Sorghum | 3D | fullfield laser | 1 | 3.5 | 2 | 54 | 2020-08-23 5:01:20 | 0 days 09:02:05 |
| 2020-08-23 14:36:24 | FS | Season 11 Sorghum | StVis | resumption of previous scan | 1 | 3.5 | 2 | 54 | 2020-08-23 15:25:32 | 0 days 00:49:08 |
| 2020-08-24 10:20:12 | FS | Season 11 Sorghum | StVis | fullfield rgb/communication issue caught ~11:55 after 20s of failure/scan paused/server rebooted/scan resumed | 1 | 3.5 | 2 | 54 | 2020-08-24 14:20:51 | 0 days 04:00:39 |
| 2020-08-25 23:35:43 | FS | Season 11 Sorghum | IR | fullfield thermal 1ppr | 1 | 3.5 |  |  | 2020-08-26 1:01:05 | 0 days 01:25:22 |
| 2020-08-27 14:27:00 | DR | Season 11 Sorghum | DJI Phantom 4 Pro |  |  | 15 | 1 | 54 | 2020-08-27 14:38:00 | 0 days 00:11:00 |
| 2020-08-28 12:34:29 | FS | Season 11 Sorghum | IR | fullfield thermal 1ppr | 1 | 3.5 |  |  | 2020-08-28 13:53:40 | 0 days 01:19:11 |
| 2020-08-28 14:02:15 | FS | Season 11 Sorghum | 3D | fullfield laser/crosses sunset | 1 | 3.5 | 2 | 54 | 2020-08-28 23:05:18 | 0 days 09:03:03 |
| 2020-08-28 23:57:20 | FS | Season 11 Sorghum | IR | fullfield thermal 1ppr | 1 | 3.5 |  |  | 2020-08-29 1:18:20 | 0 days 01:21:00 |
| 2020-08-29 1:20:23 | FS | Season 11 Sorghum | 3D | fullfield laser/crosses sunrise | 1 | 3.5 | 2 | 54 | 2020-08-29 10:21:52 | 0 days 09:01:29 |
| 2020-08-31 14:29:48 | FS | Season 11 Sorghum | StVis | resumption of previous scan | 1 | 3.5 | 2 | 54 | 2020-08-31 14:41:28 | 0 days 00:11:40 |
| 2020-08-31 20:35:21 | FS | Season 11 Sorghum | 3D | fullfield laser | 1 | 3.5 | 2 | 54 | 2020-09-01 5:38:06 | 0 days 09:02:45 |
| 2020-09-01 12:19:20 | FS | Season 11 Sorghum | IR | fullfield thermal 1ppr | 1 | 3.5 |  |  | 2020-09-01 13:37:35 | 0 days 01:18:15 |
| 2020-09-01 22:45:03 | FS | Season 11 Sorghum | IR | fullfield thermal 1ppr | 1 | 3.5 |  |  | 2020-09-02 0:07:36 | 0 days 01:22:33 |
| 2020-09-03 15:33:00 | DR | Season 11 Sorghum | DJI Phantom 4 Pro |  |  | 15 | 1 | 54 | 2020-09-03 15:44:00 | 0 days 00:11:00 |
| 2020-09-04 12:50:16 | FS | Season 11 Sorghum | IR | fullfield thermal 1ppr | 1 | 3.5 |  |  | 2020-09-04 14:08:59 | 0 days 01:18:43 |
| 2020-09-04 22:56:27 | FS | Season 11 Sorghum | IR | fullfield thermal 1ppr | 1 | 3.5 |  |  | 2020-09-05 0:18:46 | 0 days 01:22:19 |
| 2020-09-05 10:28:27 | FS | Season 11 Sorghum | StVis | fullfield RGB | 1 | 3.5 | 2 | 54 | 2020-09-05 14:24:14 | 0 days 03:55:47 |
| 2020-09-05 20:01:49 | FS | Season 11 Sorghum | 3D | fullfield laser | 1 | 3.5 | 2 | 54 | 2020-09-06 5:03:23 | 0 days 09:01:34 |
| 2020-09-07 10:36:23 | FS | Season 11 Sorghum | StVis | fullfield RGB | 1 | 3.5 | 2 | 54 | 2020-09-07 14:32:26 | 0 days 03:56:03 |
| 2020-09-07 21:33:18 | FS | Season 11 Sorghum | 3D | fullfield laser | 1 | 3.5 | 2 | 54 | 2020-09-08 6:36:22 | 0 days 09:03:04 |
| 2020-09-09 3:03:08 | FS | Season 11 Sorghum | IR | fullfield thermal 1ppr | 1 | 3.5 |  |  | 2020-09-09 4:28:03 | 0 days 01:24:55 |
| 2020-09-11 12:41:56 | FS | Season 11 Sorghum | IR | fullfield thermal 1ppr | 1 | 3.5 |  |  | 2020-09-11 14:00:06 | 0 days 01:18:10 |
| 2020-09-11 14:27:00 | DR | Season 11 Sorghum | DJI Phantom 4 Pro |  |  | 15 | 1 | 54 | 2020-09-11 14:39:00 | 0 days 00:12:00 |
| 2020-09-11 23:00:47 | FS | Season 11 Sorghum | IR | fullfield thermal 1ppr | 1 | 3.5 |  |  | 2020-09-12 0:25:52 | 0 days 01:25:05 |
| 2020-09-12 10:23:08 | FS | Season 11 Sorghum | StVis | fullfield RGB | 1 | 3.5 | 2 | 54 | 2020-09-12 14:22:33 | 0 days 03:59:25 |
| 2020-09-12 20:45:07 | FS | Season 11 Sorghum | 3D | fullfield laser | 1 | 3.5 | 2 | 54 | 2020-09-13 5:47:13 | 0 days 09:02:06 |
| 2020-09-14 12:57:13 | FS | Season 11 Sorghum | StVis | resumption of prior scan after 10 minute gap | 1 | 3.5 | 2 | 54 | 2020-09-14 14:21:59 | 0 days 01:24:46 |
| 2020-09-15 7:22:55 | FS | Season 11 Sorghum | 3D | fullfield laser/resumption of prior scan | 1 | 3.5 | 2 | 54 | 2020-09-15 7:49:10 | 0 days 00:26:15 |
| 2020-09-15 12:25:47 | FS | Season 11 Sorghum | IR | fullfield thermal 1ppr | 1 | 3.5 |  |  | 2020-09-15 13:46:36 | 0 days 01:20:49 |
| 2020-09-17 15:39:00 | DR | Season 11 Sorghum | DJI Phantom 4 Pro |  |  | 15 | 1 | 54 | 2020-09-17 15:50:00 | 0 days 00:11:00 |
| 2020-09-19 10:28:58 | FS | Season 11 Sorghum | StVis | fullfield RGB | 1 | 3.5 | 2 | 54 | 2020-09-19 14:25:25 | 0 days 03:56:27 |
| 2020-09-19 21:55:49 | FS | Season 11 Sorghum | 3D | fullfield laser | 1 | 3.5 | 2 | 54 | 2020-09-20 6:58:11 | 0 days 09:02:22 |
| 2020-09-21 10:24:03 | FS | Season 11 Sorghum | StVis | fullfield RGB | 1 | 3.5 | 2 | 54 | 2020-09-21 14:18:51 | 0 days 03:54:48 |
| 2020-09-21 20:13:56 | FS | Season 11 Sorghum | 3D | fullfield laser | 1 | 3.5 | 2 | 54 | 2020-09-22 5:19:57 | 0 days 09:06:01 |
| 2020-09-24 15:43:00 | DR | Season 11 Sorghum | DJI Phantom 4 Pro |  |  | 15 | 1 | 54 | 2020-09-24 15:54:00 | 0 days 00:11:00 |
| 2020-09-26 11:10:45 | FS | Season 11 Sorghum | StVis | fullfield RGB | 1 | 3.5 | 2 | 54 | 2020-09-26 15:06:33 | 0 days 03:55:48 |
| 2020-09-26 20:28:35 | FS | Season 11 Sorghum | 3D | fullfield laser | 1 | 3.5 | 2 | 54 | 2020-09-27 5:30:51 | 0 days 09:02:16 |
| 2020-09-28 10:29:10 | FS | Season 11 Sorghum | StVis | fullfield RGB | 1 | 3.5 | 2 | 54 | 2020-09-28 14:24:31 | 0 days 03:55:21 |
| 2020-09-28 20:07:22 | FS | Season 11 Sorghum | 3D | fullfield laser | 1 | 3.5 | 2 | 54 | 2020-09-29 5:09:53 | 0 days 09:02:31 |
| 2020-10-01 14:34:00 | DR | Season 11 Sorghum | DJI Phantom 4 Pro |  |  | 15 | 1 | 54 | 2020-10-01 14:45:00 | 0 days 00:11:00 |
| 2020-10-03 10:25:39 | FS | Season 11 Sorghum | StVis | fullfield RGB | 1 | 3.5 | 2 | 54 | 2020-10-03 14:21:20 | 0 days 03:55:41 |
| 2020-10-03 21:19:38 | FS | Season 11 Sorghum | 3D | fullfield laser | 1 | 3.5 | 2 | 54 | 2020-10-04 6:21:59 | 0 days 09:02:21 |
| 2020-10-05 10:29:56 | FS | Season 11 Sorghum | StVis | fullfield RGB | 1 | 3.5 | 2 | 54 | 2020-10-05 14:24:49 | 0 days 03:54:53 |
| 2020-10-05 19:53:47 | FS | Season 11 Sorghum | 3D | fullfield laser | 1 | 3.5 | 2 | 54 | 2020-10-06 4:56:17 | 0 days 09:02:30 |
| 2020-10-07 20:26:37 | FS | Season 11 Sorghum | 3D | fullfield laser | 1 | 3.5 | 2 | 54 | 2020-10-08 5:28:28 | 0 days 09:01:51 |
| 2020-10-09 13:16:00 | DR | Season 11 Sorghum | DJI Phantom 4 Pro |  |  | 15 | 1 | 54 | 2020-10-09 13:27:00 | 0 days 00:11:00 |
| 2020-10-10 10:22:24 | FS | Season 11 Sorghum | StVis | fullfield RGB | 1 | 3.5 | 2 | 54 | 2020-10-10 14:18:33 | 0 days 03:56:09 |
| 2020-10-10 20:46:27 | FS | Season 11 Sorghum | 3D | fullfield laser | 1 | 3.5 | 2 | 54 | 2020-10-11 5:49:00 | 0 days 09:02:33 |
| 2020-10-12 10:18:10 | FS | Season 11 Sorghum | StVis | fullfield RGB | 1 | 3.5 | 2 | 54 | 2020-10-12 14:14:00 | 0 days 03:55:50 |
| 2020-10-12 20:21:10 | FS | Season 11 Sorghum | 3D | fullfield laser | 1 | 3.5 | 2 | 54 | 2020-10-13 5:23:29 | 0 days 09:02:19 |
| 2020-10-16 13:22:00 | DR | Season 11 Sorghum | DJI Phantom 4 Pro |  |  | 15 | 1 | 54 | 2020-10-16 13:35:00 | 0 days 00:13:00 |
| 2020-10-17 20:14:50 | FS | Season 11 Sorghum | 3D | fullfield laser | 1 | 3.5 | 2 | 54 | 2020-10-18 5:17:26 | 0 days 09:02:36 |
| 2020-10-18 10:23:21 | FS | Season 11 Sorghum | StVis | fullfield RGB | 1 | 3.5 | 2 | 54 | 2020-10-18 14:18:53 | 0 days 03:55:32 |
| 2020-10-19 10:23:58 | FS | Season 11 Sorghum | StVis | Fullfield RGB | 1 | 3.5 | 2 | 54 | 2020-10-19 14:19:02 | 0 days 03:55:04 |
| 2020-10-19 19:57:05 | FS | Season 11 Sorghum | 3D | fullfield laser | 1 | 3.5 | 2 | 54 | 2020-10-20 4:59:27 | 0 days 09:02:22 |
| 2020-10-23 13:31:00 | DR | Season 11 Sorghum | DJI Phantom 4 Pro |  |  | 15 | 1 | 54 | 2020-10-23 13:44:00 | 0 days 00:13:00 |
| 2020-10-24 10:20:29 | FS | Season 11 Sorghum | StVis | fullfield RGB | 1 | 3.5 | 2 | 54 | 2020-10-24 14:16:42 | 0 days 03:56:13 |
| 2020-10-24 20:15:17 | FS | Season 11 Sorghum | 3D | fullfield 3D | 1 | 3.5 | 2 | 54 | 2020-10-25 5:17:38 | 0 days 09:02:21 |
| 2020-10-28 10:29:24 | FS | Season 11 Sorghum | StVis | fullfield rgb | 1 | 3.5 | 2 | 54 | 2020-10-28 14:24:51 | 0 days 03:55:27 |
| 2020-10-28 18:34:15 | FS | Season 11 Sorghum | 3D | fullfield laser | 1 | 3.5 | 2 | 54 | 2020-10-29 3:36:21 | 0 days 09:02:06 |
| 2020-10-30 13:53:00 | DR | Season 11 Sorghum | DJI Phantom 4 Pro |  |  | 15 | 1 | 54 | 2020-10-30 14:06:00 | 0 days 00:13:00 |
| 2020-10-31 11:08:14 | FS | Season 11 Sorghum | StVis | fullfield rgb | 1 | 3.5 | 2 | 54 | 2020-10-31 15:03:15 | 0 days 03:55:01 |
| 2020-10-31 20:28:12 | FS | Season 11 Sorghum | 3D | fullfield laser | 1 | 3.5 | 2 | 54 | 2020-11-01 5:30:06 | 0 days 09:01:54 |
| 2020-11-06 12:11:00 | DR | Season 11 Sorghum | DJI Phantom 4 Pro |  |  | 15 | 1 | 54 | 2020-11-06 12:24:00 | 0 days 00:13:00 |
|  | FS | Season 10 Lettuce | 3D | south-to-north fullfield 3D | 0.05 | 3.5 | 2 | 53 | 2020-01-06 3:35:11 |  |

[**Supplementary Table 3**](https://docs.google.com/document/d/1b-jKFdFwnQ65CANT0oN1dD-hPOgK-ls4/edit#bookmark=id.1fob9te). Container information including GitHub and DockerHub container repository web links.

| **Sensor pipeline** | **Data type** | **Sequence order** | **Container name** | **GitHub repository link** | **DockerHub repository link** | **Field scanalyzer specific** |
| --- | --- | --- | --- | --- | --- | --- |
| RGB | 2D image | 1 | rgb_bin_to_tif | <https://github.com/phytooracle/rgb_bin_to_tif> | <https://hub.docker.com/repository/docker/phytooracle/rgb_bin_to_tif> | Y |
|  |  | 2 | rgb_flir_collect_gps | <https://github.com/phytooracle/rgb_flir_collect_gps> | <https://hub.docker.com/repository/docker/phytooracle/rgb_flir_collect_gps> | N |
|  |  | 3 | megastitch | <https://github.com/ariyanzri/MegaStitch> | <https://hub.docker.com/r/ariyanzarei/geo-correction> | N |
|  |  | 4 | rgb_flir_edit_gps | <https://github.com/phytooracle/rgb_flir_edit_gps> | <https://hub.docker.com/repository/docker/phytooracle/rgb_flir_edit_gps> | N |
|  |  | 5 | rgb_flir_plot_clip_geojson | <https://github.com/phytooracle/rgb_flir_plot_clip_geojson> | <https://hub.docker.com/repository/docker/phytooracle/rgb_flir_plot_clip_geojson> | N |
|  |  | 6 | gdal | <https://github.com/OSGeo/gdal> | <https://hub.docker.com/r/osgeo/gdal> | N |
|  |  | 7 | rgb_flir_plant_detection | <https://github.com/phytooracle/rgb_flir_plant_detection> | <https://hub.docker.com/repository/docker/phytooracle/rgb_flir_plant_detection> | N |
| Thermal |  | 1 | flir_bin_to_tif_s10 | <https://github.com/phytooracle/flir_bin_to_tif_s10> | <https://hub.docker.com/repository/docker/phytooracle/flir_bin_to_tif_s10> | Y |
|  |  | 2 | rgb_flir_collect_gps | <https://github.com/phytooracle/rgb_flir_collect_gps> |  | N |
|  |  | 3 | megastitch | <https://github.com/ariyanzri/MegaStitch> | <https://hub.docker.com/r/ariyanzarei/geo-correction> | N |
|  |  | 4 | rgb_flir_edit_gps | <https://github.com/phytooracle/rgb_flir_edit_gps> | <https://hub.docker.com/repository/docker/phytooracle/rgb_flir_edit_gps> | N |
|  |  | 5 | flir_field_stitch | <https://github.com/phytooracle/flir_field_stitch> | <https://hub.docker.com/repository/docker/phytooracle/flir_field_stitch> | N |
|  |  | 6 | rgb_flir_plot_clip_geojson | <https://github.com/phytooracle/rgb_flir_plot_clip_geojson> | <https://hub.docker.com/repository/docker/phytooracle/rgb_flir_plot_clip_geojson> | N |
|  |  | 7 | flir_plant_temp | <https://github.com/phytooracle/flir_plant_temp> | <https://hub.docker.com/repository/docker/phytooracle/flir_plant_temp> | N |
|  |  | 8 | flir_meantemp | <https://github.com/phytooracle/flir_meantemp> | <https://hub.docker.com/repository/docker/phytooracle/flir_meantemp> | N |
| PSII |  | 1 | psii_bin_to_tif | <https://github.com/phytooracle/psii_bin_to_tif> | <https://hub.docker.com/repository/docker/phytooracle/psii_bin_to_tif> | N |
|  |  | 2 | rgb_flir_plot_clip_geojson | <https://github.com/phytooracle/rgb_flir_plot_clip_geojson> | <https://hub.docker.com/repository/docker/phytooracle/rgb_flir_plot_clip_geojson> | N |
|  |  | 3 | psii_segmentation | <https://github.com/phytooracle/psii_segmentation> | <https://hub.docker.com/repository/docker/phytooracle/psii_segmentation> | N |
|  |  | 4 | psii_fluorescence_aggregation | <https://github.com/phytooracle/psii_fluorescence_aggregation> | <https://hub.docker.com/repository/docker/phytooracle/psii_fluorescence_aggregation> | N |
| 3D | 3D point cloud | 1 | 3d_preprocessing | <https://github.com/phytooracle/3d_preprocessing> | <https://hub.docker.com/repository/docker/phytooracle/3d_preprocessing> | Y |
|  |  | 2 | 3d_landmark_selection | <https://github.com/phytooracle/3d_landmark_selection> | <https://hub.docker.com/repository/docker/phytooracle/3d_landmark_selection> | N |
|  |  | 3 | 3d_postprocessing | <https://github.com/phytooracle/3d_postprocessing> | <https://hub.docker.com/repository/docker/phytooracle/3d_postprocessing> | Y |
|  |  | 4 | 3d_crop_individiual_plants | <https://github.com/phytooracle/3d_crop_individual_plants> | <https://hub.docker.com/repository/docker/phytooracle/3d_crop_individual_plants> | N |
|  |  | 5 | 3d_individual_plant_registration | <https://github.com/phytooracle/3d_individual_plant_registration> | <https://hub.docker.com/repository/docker/phytooracle/3d_individual_plant_registration> | N |
|  |  | 6 | 3d_heatmap_detection | <https://github.com/phytooracle/3d_heatmap_detection> | <https://hub.docker.com/repository/docker/phytooracle/heatmap_ml_based_cropping> | N |
|  |  | 7 | 3d_single_plant_soil_segmentation | <https://github.com/phytooracle/PhytoOracle_Lettuce_Soil_Annotator> | <https://hub.docker.com/repository/docker/phytooracle/dgcnn_single_plant_soil_segmentation_pipeline> | N |
|  |  | 8 | 3d_neighbor_removal | <https://github.com/phytooracle/3d_neighbor_removal> | <https://hub.docker.com/repository/docker/phytooracle/3d_neighbor_removal> | N |
|  |  | 9 | 3d_segmentation_dashboard_assets | <https://github.com/phytooracle/3d_segmentation_dashboard_assets> | <https://hub.docker.com/repository/docker/phytooracle/3d_segmentation_dashboard_assets> | N |

[**Supplementary Table 4**](https://docs.google.com/document/d/1b-jKFdFwnQ65CANT0oN1dD-hPOgK-ls4/edit#bookmark=id.3znysh7). List of Python libraries and version numbers used within PhytoOracle containers.

| **Library** | **Version** |
| --- | --- |
| pdal |  |
| liblas-bin |  |
| GDAL | 3.0.4 |
| numpy | 1.19.1 |
| matplotlib | 3.2.1 |
| pandas | 1.0.3 |
| liblas |  |
| geopandas |  |
| pyproj |  |
| utm |  |
| tifffile |  |
| opencv-python |  |
| tqdm |  |
| detecto |  |
| imagecodecs |  |
| scikit-learn |  |
| terrautils |  |
| scikit-build |  |
| cmake |  |
| laspy |  |
| Cctools |  |
| Makeflow |  |
| setuptools |  |
| ExifRead | 2.3.1 |
| gpsphoto | 2.2.3 |
| imageio | 2.8.0 |
| network | 2.4 |
| piexif | 1.1.3 |
| Pillow | 7.1.2 |
| pytz | 2020.1 |
| PyWavelets | 1.1.1 |
| scikit-image | 0.17.2 |
| scipy | 1.4.1 |
| six | 1.14.0 |
| libgl1-mesa-dev |  |
| Open3d |  |
| tensorflow |  |
| keras |  |
